# Proteomic changes in cerebrospinal fluid from primary central nervous system lymphoma patients are associated with protein ectodomain shedding

**DOI:** 10.18632/oncotarget.22654

**Published:** 2017-11-24

**Authors:** Daniel Michael Waldera-Lupa, Omid Etemad-Parishanzadeh, Mareike Brocksieper, Nina Kirchgaessler, Sabine Seidel, Thomas Kowalski, Manuel Montesinos-Rongen, Martina Deckert, Uwe Schlegel, Kai Stühler

**Affiliations:** ^1^ Molecular Proteomics Laboratory, Institute of Molecular Medicine, Universitaetsklinikum Düsseldorf, Düsseldorf, Germany; ^2^ Department of Neurology, Knappschaftskrankenhaus, Ruhr-University Bochum, Bochum, Germany; ^3^ Institute of Neuropathology, University of Cologne, Cologne, Germany; ^4^ Biologisch-Medizinisches Forschungszentrum, Heinrich-Heine-University Düsseldorf, Düsseldorf, Germany

**Keywords:** primary central nervous system lymphoma, proteomics, cerebrospinal fluid, ectodomain shedding, protein secretion

## Abstract

Primary central nervous system lymphomas (PCNSLs) are mature B-cell lymphomas confined to the central nervous system (CNS). Blood-brain barrier (BBB) dysfunction drastically alters the cerebrospinal fluid (CSF) proteome in PCNSL patients. To reveal the interaction of PCNSL tumors with CNS structures and the vasculature, we conducted a whole-proteome analysis of CSF from PCNSL patients (*n* = 17 at initial diagnosis) and tumor-free controls (*n* = 10) using label-free quantitative mass spectrometry. We identified 601 proteins in the CSF proteome using a one-step approach without further prefractionation, and quantified 438 proteins in detail using the Hi-N method. An immunoassay revealed that 70% of the patients in our unselected PCNSL patient cohort had BBB dysfunction. Correlation analysis indicated that 127 (30%) of the quantified proteins were likely increased in PCSNL patients due to BBB dysfunction. After the exclusion of these proteins, 66 were found to differ in abundance (fold-change > 2.0, *p* < 0.05) between PCNSL and control CSF proteomes, and most of those were associated with the CNS. These data also provide the first evidence that proteomic changes in CSF from PCNSL patients are mainly associated with protein ectodomain shedding, and that shedding of human leukocyte antigen class 2 proteins is a mechanism of tumor-cell immune evasion.

## INTRODUCTION

Primary central nervous system lymphomas (PCNSLs) are mature B-cell lymphomas of the diffuse large B-cell lymphoma (DLBCL) type that are confined to the central nervous system (CNS) [1, 2]. PCNSLs account for about 3% of primary CNS tumors, and occur more frequently in patients with immunodeficiency (for instance, due to HIV infection or posttransplant medical immunosuppression) [1, 2]. PCNSLs disseminate within the brain, spinal cord, leptomeninges and cerebrospinal fluid (CSF), as well as the vitreous and chorioretina in about 10% of patients (which is considered a manifestation of CNS lymphoma), and carry a less favorable prognosis than systemic DLBCL [3, 4]. As PCNSLs are usually in close proximity to the ventricular system, CSF is a possible matrix for the investigation of the tumor-environment interaction [5].

Proteins in normal CSF are mainly derived from three different sources: plasma, neural tissue and cells within the CSF [6]. Proteins from neural tissue and tumor cells can be released either passively by tissue leakage due to apoptotic/necrotic processes, or actively by classical or unconventional secretion [7] involving ectodomain shedding [8] or vesicular transport [9]. Recently, bioanalytical approaches have demonstrated that CSF has a complex proteome with more than 2800 proteins [10–14]. The CSF of PCNSL patients has been reported to contain increased levels of cytokines such as interleukin (IL)6, IL-10, CXCL-12 and CXCL-13 [15–20], as well as the ectodomains of transmembrane proteins (sCD27 and sIL-2R) [15, 18, 21, 22].

Here, we used label-free liquid chromatography-mass spectrometry (LC-MS) to detect differences in the CSF proteomes of PCNSL patients and tumor-free controls, and to investigate the interaction of this lymphoma with its microenvironment.

## RESULTS

PCNSLs occur most frequently in close proximity to the ventricular system. Therefore, we analyzed CSF from PCNSL patients and tumor-free controls to determine the proteomic signature reflecting the interaction of PCNSL tumors with CNS structures and the vasculature. In total, 17 PCNSL patients and 10 age- and sex-matched tumor-free individuals were analyzed with a quantitative proteomic approach (Table 1; for diagnoses, see Supplementary Table 1A).

**Table 1:** Demographic data of participating individuals

	Tumor-free control	PCNSL
Individuals	10	17
Male	5	8
Female	5	9
Age ± SD	67.0 ± 5.5	63.9 ± 9.1

The ages are shown as the mean values of the groups with the corresponding standard deviations. The PCNSL group included patients treated with steroids prior to the extraction of CSF (10 individuals) and patients not treated with steroids (7 individuals).

### Analysis of blood-brain barrier (BBB) dysfunction in PCNSL patients

**Figure 1:**
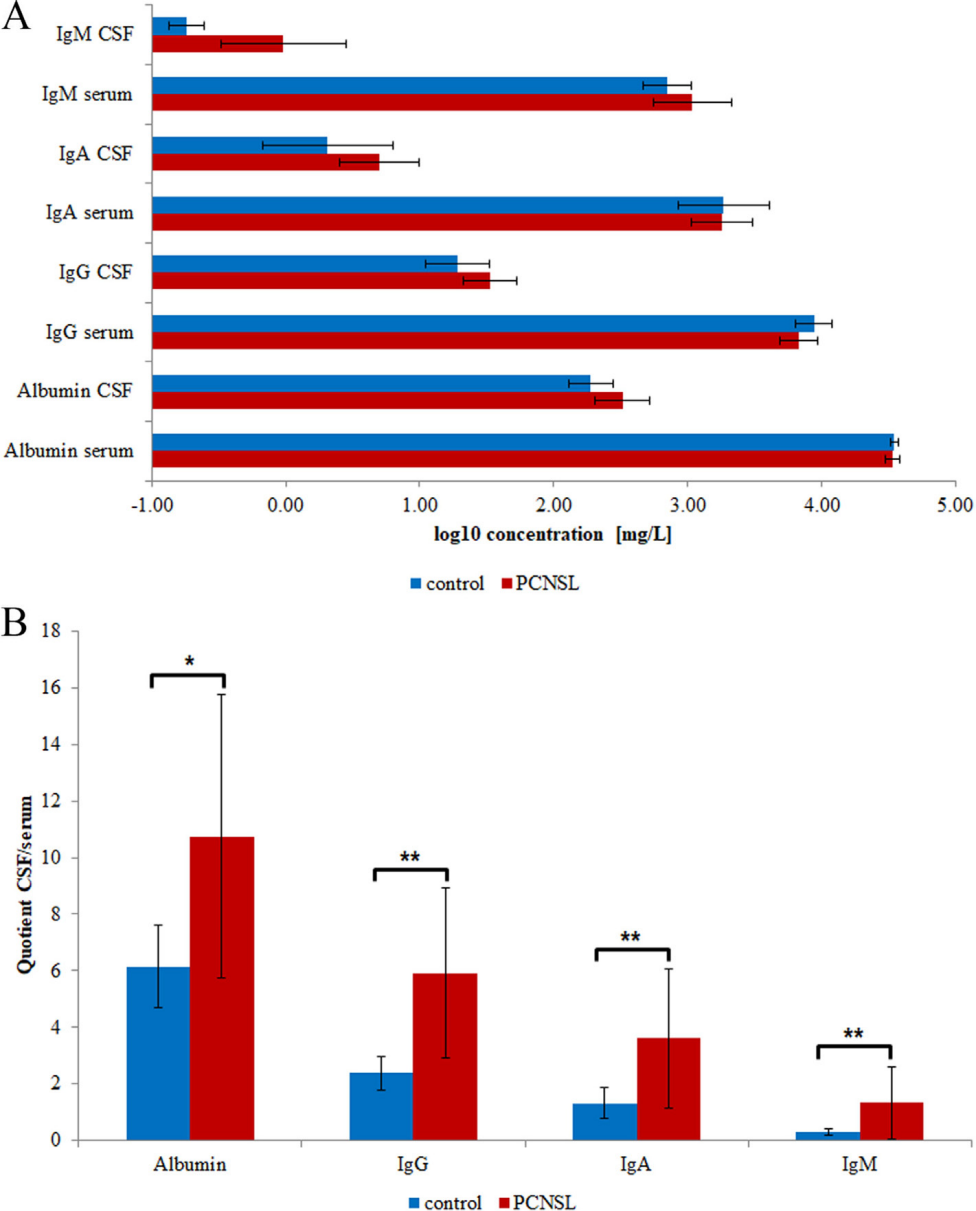
Clinical data of participating individuals The mean values and corresponding standard deviations are shown. (**A**) Concentrations of albumin, IgG, IgA and IgM in CSF and serum. (**B**) CSF/serum concentration quotients of albumin, IgG, IgA and IgM. Significance levels: *p* < 0.05 (^*^), *p* < 0.01 (^**^).

PCNSL is frequently associated with BBB dysfunction, which allows plasma proteins to leak into the CSF, significantly changing its proteomic signature. To control for the extent of BBB dysfunction, we used commercial immunoassays to determine the concentrations of albumin, IgG, IgA and IgM in CSF and serum (Figure 1A, Supplementary Table 1A). We calculated the CSF/serum quotients of albumin, IgG, IgA and IgM to determine whether the elevated plasma protein concentrations in CSF resulted from BBB dysfunction [23]. The age-dependent concentration quotients revealed that the albumin, IgG, IgA and IgM levels in CSF were significantly elevated in 12 of the 17 PCNSL patients and in one of the control patients (Figure 1B, Supplementary Table 1B). Thus, we confirmed that 70% of the analyzed PCNSL patients had BBB dysfunction.

### Proteomic signature of CSF from PCNSL patients

**Figure 2:**
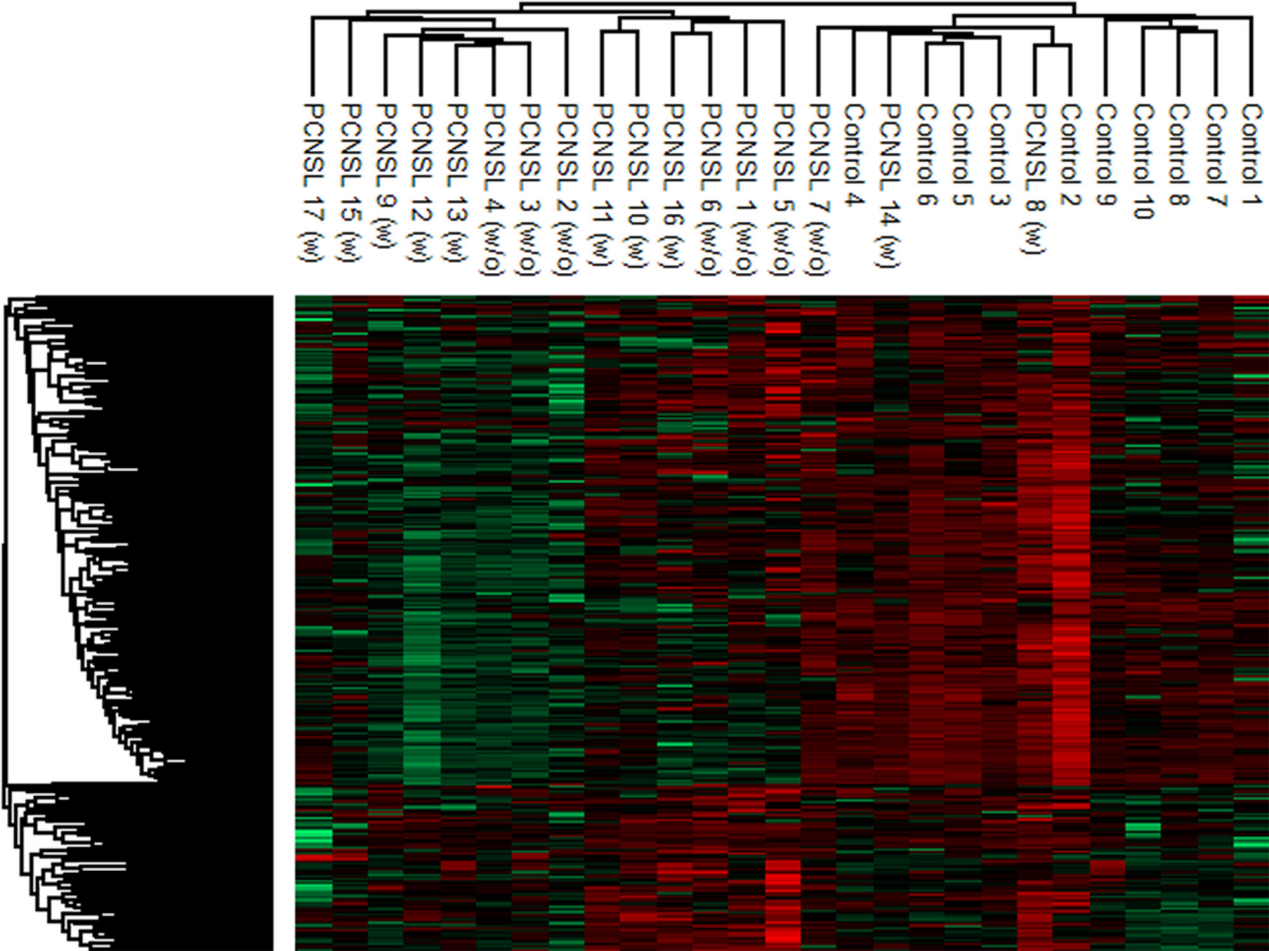
Hierarchical cluster analysis of the CSF proteome in PCNSL and control patients For the comparison, only proteins (306 proteins) not correlating with the albumin concentration were considered. The comparable proteomic signatures of PCNSL patients treated (10 patients) or untreated (7 patients) with steroids led to the decision to include all PCNSL patients in one group for further data interrogation.

**Figure 3:**
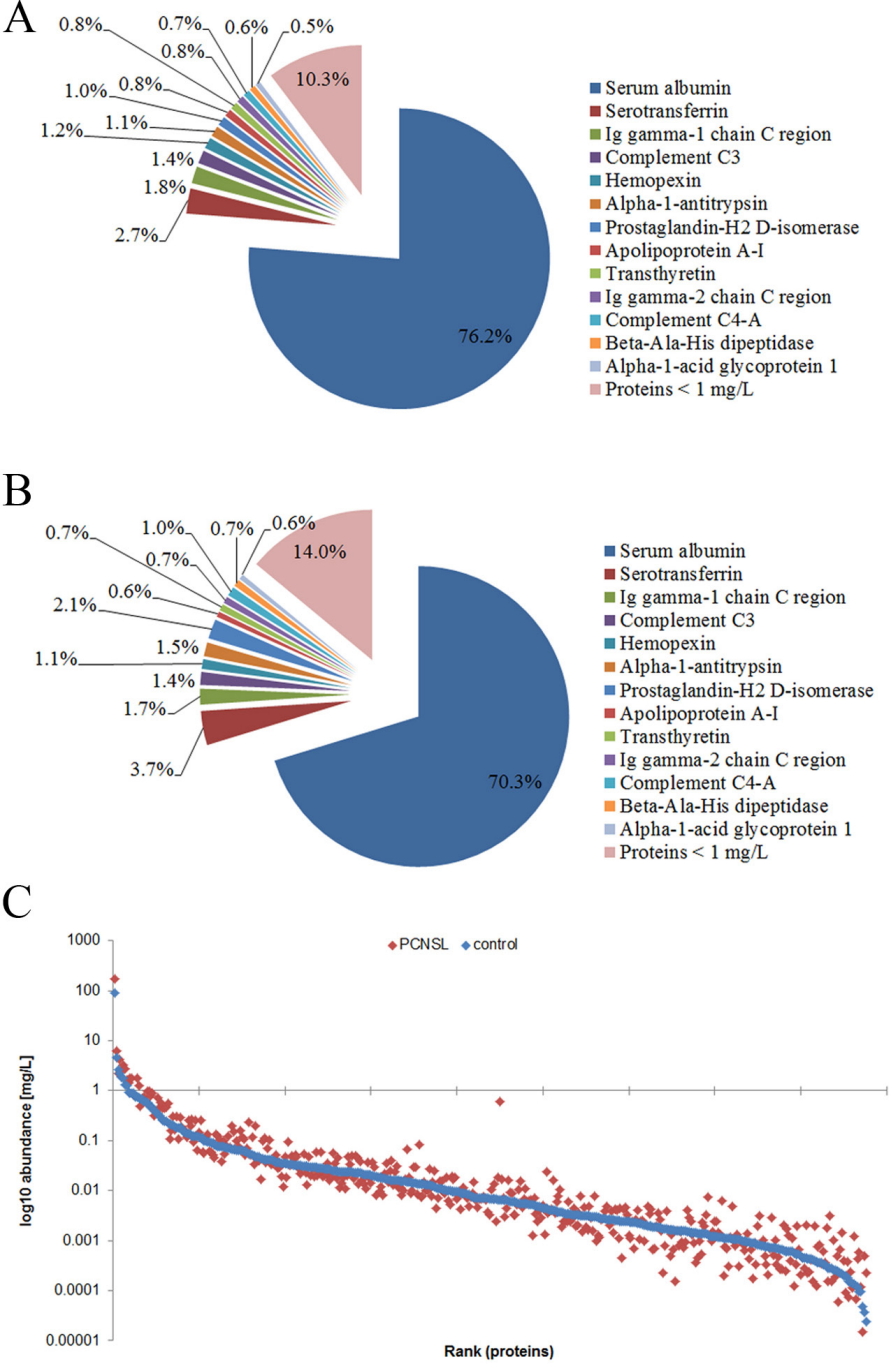
Characterization of the PCNSL CSF proteome Altogether, 601 and 438 proteins were identified and quantified in the CSF, respectively. A/B. Distribution of quantified proteins in PCNSL patients (**A**) and tumor-free controls (**B**) in percentages. Proteins with concentrations >10^-5^ μg/μL are shown separately, whereas proteins < 10^-5^ μg/μL are summed. (**C**) Abundance range of quantified proteins. Abundances are shown in log10 scale (mean concentration of each group; blue: control group, red: PCNSL patients).

We next used quantitative LC-electrospray ionization (ESI)-MS/MS to compare the CSF proteomic signatures of PCNSL patients (with and without steroid treatment) and tumor-free patients. The CSF proteomic signatures were comparable in PCNSL patients with and without steroid treatment; thus, all the PCNSL patients were grouped together for further data analysis (Figure 2). Using label-free MS, we identified a total of 601 proteins in the CSF, and we quantified 438 of them for which sufficient peptide information (*n* ≥ 2) was available. Detailed quantification of these 438 proteins revealed a group of 13 bona fide plasma proteins (serum albumin, serotransferrin, immunoglobulin heavy constant gamma 1, complement C3, hemopexin, alpha-1-antitrypsin, prostaglandin-H2 D-isomerase, apolipoprotein A-I, transthyretin, immunoglobulin heavy constant gamma 2, complement C4-A, beta-Ala-His dipeptidase and alpha-1-acid glycoprotein 1) with concentrations above 1 mg/L, comprising 90% and 86% of the total protein amounts in PCNSL patients and tumor-free patients, respectively (Figure 3A and 3B). Albumin was the most abundant protein (mean concentration of 174.6 ± 113.7 mg/L in PCNSL patients), with an average proportion of around 76%. The protein abundances were distributed in a dynamic range of approximately five orders of magnitude for both groups, with the highest concentration for albumin (174.6 ± 113.7 mg/L, PCNSL; 88.7 ± 37.4 mg/L, control) and the lowest for selenoprotein M (1.5 × 10^−5^ ± 2.3 × 10^−5^ mg/L, PCNSL; 4.8 × 10^−5^ ± 4.5 × 10^−5^ mg/L, control) (Figure 3C). For four proteins (albumin, IgG, IgA and IgM), the concentrations could be confirmed with commercial immunoassays. These concentrations correlated significantly with the detailed protein concentrations calculated by MS with the Hi-N method (Supplementary Figure 1), demonstrating the accuracy of this method.

**Figure 4:**
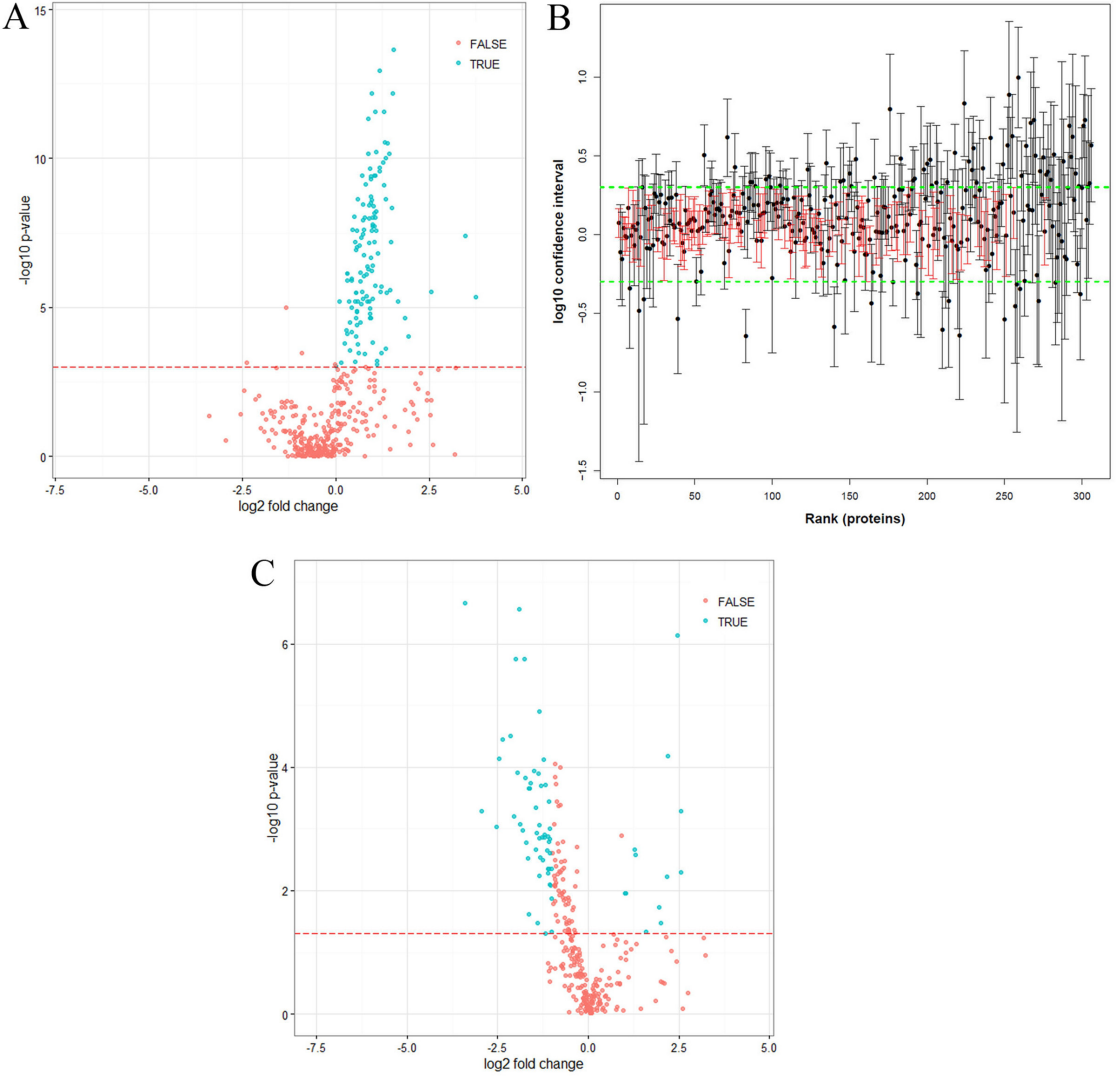
Statistical analysis of quantified proteins (**A**) Volcano plot of proteins significantly correlating with CSF albumin, measured by LC-MS (Pearson correlation, *p*-values corrected with Benjamini-Hochberg correction, Supplementary Table 2). The red line indicates a *p*-value of *p* = 0.001. Proteins marked as “true” (*p* < 0.001) correlated significantly with CSF albumin (positive fold-change, more abundant in PCNSL patients; negative fold-change, more abundant in the control group). (**B**) Distributions of confidence intervals of proteins not correlating with albumin (Supplementary Table 4). The green line indicates the upper and lower limits calculated from the technical variance (three times the standard deviation). Unchanged proteins (104) are indicated in red. Proteins were ranked according to their abundances (from high to low). (**C**) Volcano plot of proteins not correlating with albumin (306 proteins) (Supplementary Table 5). The red line indicates a *p*-value of *p* = 0.05. Proteins marked as “true” (*p* < 0.05, fold-change > 2) differed significantly in abundance between the control and PCNSL patients, whereas proteins marked with “false” did not differ significantly (positive fold-change, more abundant in PCNSL patients; negative fold-change, more abundant in the control group).

As we confirmed that a high proportion of the PCNSL group had BBB dysfunction, we sought to evaluate which proteins had likely leaked into the CSF. To this end, we determined the correlation between the CSF concentration of each identified protein and the CSF albumin concentration. The concentrations of 127 proteins (30% of the proteins quantified) significantly correlated with the CSF albumin concentration, representing the group of BBB leakage proteins (Figure 4A, Supplementary Table 2). The UniProt tissue annotation database indicated that at least 89 of these proteins have been experimentally detected in one of four plasma-associated tissues (72 plasma proteins, *p* = 5.7 × 10^−53^; 75 liver proteins, *p* = 1.4 × 10^−26^; 7 serum proteins, *p* = 4.1 × 10^−4^; and 17 blood proteins, *p* = 4.7 × 10^−4^) (Supplementary Figure 2).

After the removal of the BBB leakage proteins, a total of 306 proteins were classified as the “PCNSL CSF proteome” (Supplementary Table 3). Again, by means of the UniProt tissue annotation database, we discovered that this group of 306 proteins exhibited significant enrichment of 185 brain-derived proteins (*p* = 1.9 × 10^−2^). However, 64 proteins were experimentally proven to be abundant in plasma and liver, confirming that CSF is an ultrafiltrate of blood plasma (69 plasma proteins, *p* = 3.5 × 10^−15^; and 105 liver proteins, *p* = 1.3 × 10^−4^) (Supplementary Figure 3). Interestingly, 104 proteins exhibited a stable abundance and did not differ significantly between the PCNSL and control groups; these can be regarded as the “CSF core proteome” (Figure 4B, Supplementary Table 4). Differential analysis of the CSF proteome (306 proteins; Supplementary Table 5) revealed that 66 proteins differed significantly (*p* < 0.05, fold-change > 2.0) between the PCNSL and control CSF proteomes, of which 12 proteins were more abundant in the PCNSL CSF proteome and 54 proteins were more abundant in the control CSF proteome (Table 2, Figure 4C).

**Table 2:** Differentially abundant proteins in PCNSL patients and tumor-free controls

UniProt accession	Gene name	*p*-value	Fold change	Concentration control [mg/L]	Concentration PCNSL [mg/L]	Tissue proteome	Tissue RNA	Secretion prediction
P04040	CAT	4.99E-03	5.9	3.10E-04	1.81E-03	X	X	Extra.
Q9Y279	VSIG4	5.15E-04	5.8	1.31E-03	7.62E-03		X	Extra./SP/TM
Q86VB7-2	CD163	7.23E-07	5.5	4.37E-03	2.39E-02		X	Extra./SP/Sec./TM
Q14956	GPNMB	6.68E-05	4.6	4.45E-04	2.04E-03	X	X	SP/TM
Q9Y6R7	FCGBP	5.95E-03	4.5	1.51E-02	6.80E-02			Extra./SP/Sec.
Q9NZK5	CECR1	3.36E-02	4.0	2.55E-04	1.02E-03			Extra./SP/Sec.
P0DJI8	SAA1	1.88E-02	3.8	7.36E-04	2.83E-03			Extra./SP/Sec.
P18428	LBP	4.60E-02	3.0	2.23E-04	6.69E-04			Extra./SP/Sec.
P02747	C1QC	2.62E-03	2.5	1.61E-02	3.95E-02			Extra./SP/Sec.
P22897	MRC1	2.13E-03	2.4	1.38E-03	3.32E-03			SP/TM
P02746	C1QB	1.12E-02	2.1	1.73E-02	3.55E-02		X	Extra./SP/Sec.
P01911	HLA-DRB1	1.09E-02	2.0	3.71E-04	7.51E-04		X	Extra./SP/TM
Q6UXD5-5	SEZ6L2	4.64E-02	-2.0	3.79E-03	1.88E-03			SP/TM
Q9UMF0	ICAM5	4.44E-03	-2.0	2.21E-03	1.09E-03			SP/TM
P55285	CDH6	1.33E-02	-2.0	1.60E-03	7.88E-04			Extra./SP/TM
Q16849	PTPRN	8.22E-03	-2.1	4.95E-03	2.39E-03		X	SP/TM
Q86UX2	ITIH5	1.47E-03	-2.1	3.16E-03	1.50E-03			Extra./SP/Sec.
Q9NX62	IMPAD1	2.43E-03	-2.1	2.09E-03	9.92E-04			TM
Q8TAG5-2	VSTM2A	7.93E-03	-2.1	6.96E-03	3.31E-03			Extra./SP/Sec.
Q92859	NEO1	9.98E-04	-2.1	2.41E-02	1.15E-02			SP/TM
Q53EL9	SEZ6	3.60E-04	-2.1	6.45E-03	3.06E-03			Extra./SP/TM
Q92520	FAM3C	1.63E-03	-2.1	2.39E-02	1.12E-02			Extra./SP/Sec.
Q96GW7	BCAN	4.50E-03	-2.1	1.77E-02	8.25E-03	X		Extra./SP/Sec.
P47972	NPTX2	5.14E-03	-2.2	7.11E-04	3.30E-04			Extra./SP/Sec.
Q9NYQ8	FAT2	1.34E-03	-2.2	9.03E-03	4.18E-03			Extra./SP/TM
Q16769	QPCT	4.37E-03	-2.2	1.72E-03	7.91E-04			Extra./SP/Sec.
P07686	HEXB	2.24E-03	-2.2	9.44E-04	4.29E-04			Extra./SP
Q8WZA1	POMGNT1	4.93E-02	-2.3	2.50E-03	1.11E-03			TM
Q9Y646	CPQ	1.91E-04	-2.3	2.91E-03	1.28E-03			Extra./SP/Sec.
Q9H3G5	CPVL	1.38E-03	-2.3	7.55E-04	3.29E-04			Extra./SP
P54764	EPHA4	1.24E-03	-2.3	3.04E-02	1.31E-02			SP/TM
O00468	AGRN	7.54E-05	-2.3	1.89E-02	8.08E-03		X	Extra./SP/Sec.
Q9NYX4	CALY	3.21E-03	-2.4	4.97E-04	2.10E-04			TM
P60174	TPI1	1.35E-03	-2.4	2.90E-03	1.18E-03	X	X	Extra.
Q9Y287	ITM2B	1.99E-04	-2.5	1.62E-03	6.58E-04		X	Extra./Sec./TM
P42785-2	PRCP	2.90E-03	-2.5	6.23E-04	2.48E-04		X	Extra./SP
O75787	ATP6AP2	1.24E-05	-2.5	6.77E-04	2.67E-04		X	Extra./SP/TM
Q9UPU3	SORCS3	1.43E-03	-2.6	1.15E-02	4.49E-03			SP/TM
Q96B86-4	RGMA	8.69E-04	-2.6	1.39E-03	5.43E-04			Extra./SP/GPI
Q9Y2I2	NTNG1	5.67E-03	-2.6	6.80E-04	2.64E-04			SP/Sec./GPI
P78509	RELN	1.28E-04	-2.6	4.43E-02	1.70E-02			Extra./SP/Sec.
Q9ULF5	SLC39A10	3.38E-02	-2.6	8.68E-04	3.29E-04			SP/TM
P22748	CA4	1.15E-03	-2.7	4.10E-04	1.54E-04			Extra./SP/GPI
P23470	PTPRG	4.45E-04	-2.7	6.70E-03	2.47E-03			Extra./SP/TM
O94910	ADGRL1	2.13E-03	-2.7	1.89E-03	6.89E-04			SP/TM
P23284	PPIB	1.15E-04	-2.8	1.92E-03	6.75E-04	X	X	Extra./SP
Q9P2S2	NRXN2	1.83E-04	-3.0	3.56E-02	1.18E-02			SP/TM
Q86UN3	RTN4RL2	2.22E-04	-3.1	2.98E-03	9.72E-04			Extra./SP/GPI
Q9UHL4	DPP7	2.42E-02	-3.1	3.82E-04	1.23E-04			Extra./SP/Sec.
Q9H2E6-2	SEMA6A	2.21E-04	-3.1	1.42E-03	4.52E-04			SP/TM
Q8WWX9	SELM	3.01E-03	-3.2	4.80E-05	1.52E-05			SP
Q99674-5	CGREF1	1.65E-03	-3.3	4.39E-03	1.34E-03			Extra./SP/Sec.
Q6NW40	RGMB	1.46E-04	-3.3	1.65E-03	4.97E-04			SP/GPI
P22304	IDS	1.77E-06	-3.4	2.31E-03	6.85E-04		X	SP
P40925-3	MDH1	1.06E-03	-3.5	1.05E-03	3.02E-04	X	X	Extra.
Q9HAT2	SIAE	8.45E-04	-3.7	7.47E-04	2.04E-04			Extra./SP/Sec.
P09417	QDPR	2.71E-07	-3.7	1.12E-03	3.01E-04		X	Extra.
Q9Y5I4	PCDHAC2	1.23E-04	-3.9	1.43E-03	3.69E-04			SP/TM
O15335	CHAD	1.77E-06	-4.0	2.40E-04	6.02E-05			Extra./SP/Sec.
O60241	ADGRB2	6.29E-04	-4.1	5.19E-03	1.25E-03			SP/TM
Q9BZR6	RTN4R	3.12E-05	-4.5	5.60E-04	1.26E-04			Extra./SP/GPI
Q8IV08	PLD3	3.49E-05	-5.2	9.09E-04	1.75E-04		X	Extra./TM
Q6MZW2	FSTL4	7.29E-05	-5.5	2.46E-03	4.48E-04			Extra./SP/Sec.
O14917	PCDH17	9.30E-04	-5.8	1.14E-03	1.95E-04			SP/TM
P10586	PTPRF	5.14E-04	-7.8	1.74E-03	2.25E-04			Extra./SP/TM
Q96FE5	LINGO1	2.19E-07	-10.5	1.61E-03	1.54E-04			SP/TM

Only proteins without a significant correlation with CSF albumin were considered as altered by the tumors. Column headers: fold-change, more abundant in PCNSL patients (positive), more abundant in the control group (negative); Concentration control/PCNSL, mean concentration of each group; Tissue proteome/RNA, proteins identified in proteome and/or RNA discovery of tumor tissue. Secretion prediction abbreviations: Extra., extracellular (GO); SP, signal peptide (UniProt); Sec., secreted (UniProt); TM, transmembrane with or without extracellular domain (UniProt); GPI, glycosylphosphatidylinositol anchor for membrane binding (UniProt).

### Origin of the altered CSF proteome in PCNSL patients

**Figure 5:**
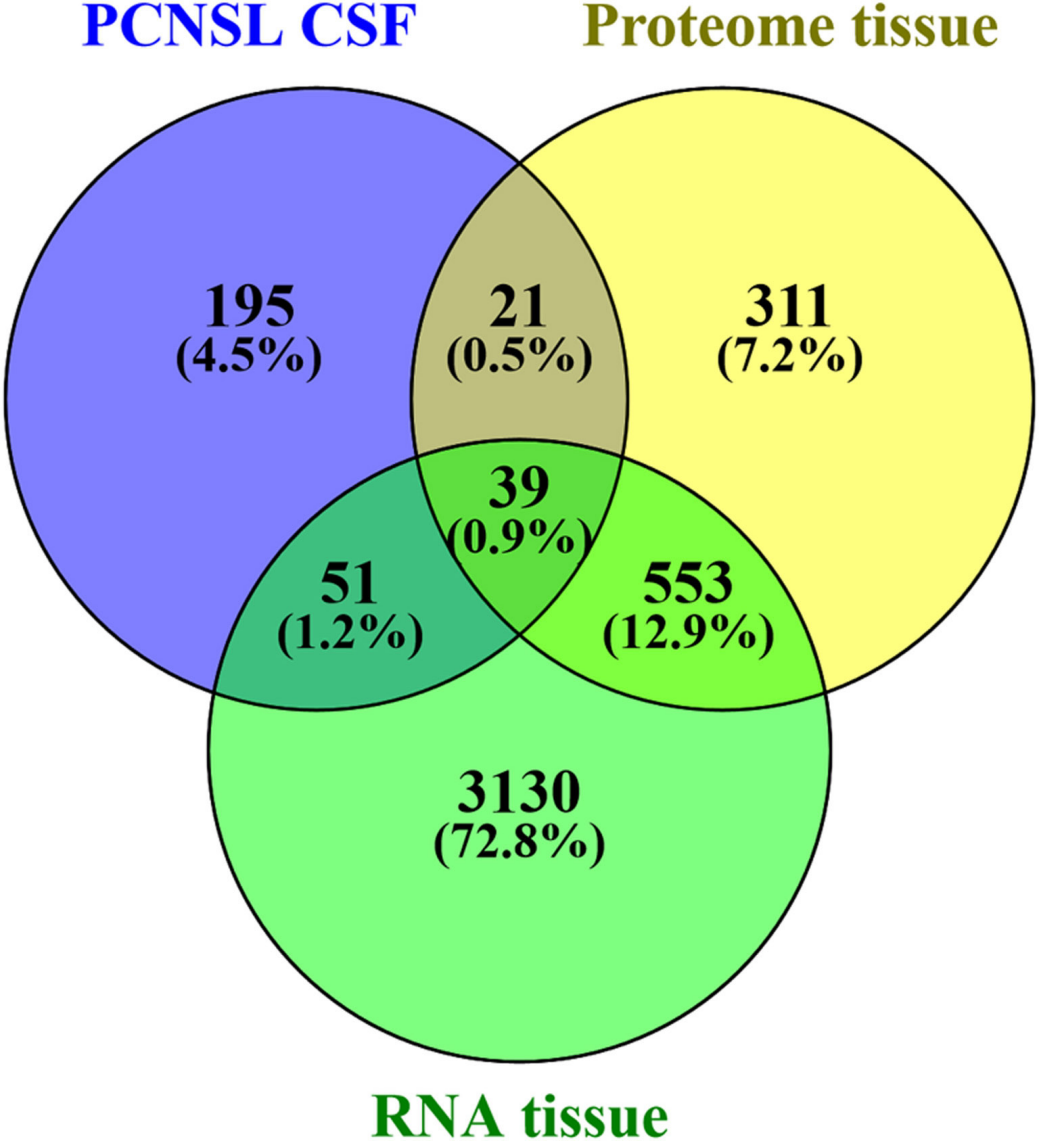
Comparison of differentially abundant CSF protein data and tumor tissue data The tumor tissue proteome (930 proteins, 1% false discovery rate, ≥ 2 peptides per protein) consisted of 6 independent PCNSL patients (unpublished mass spectrometry data), and the transcriptome study consisted of 21 independent PCNSL patients (3773 genes, occurring in at least 75% of the patients, as published by Montesinos-Rongen in 2008 [24]).

**Figure 6:**
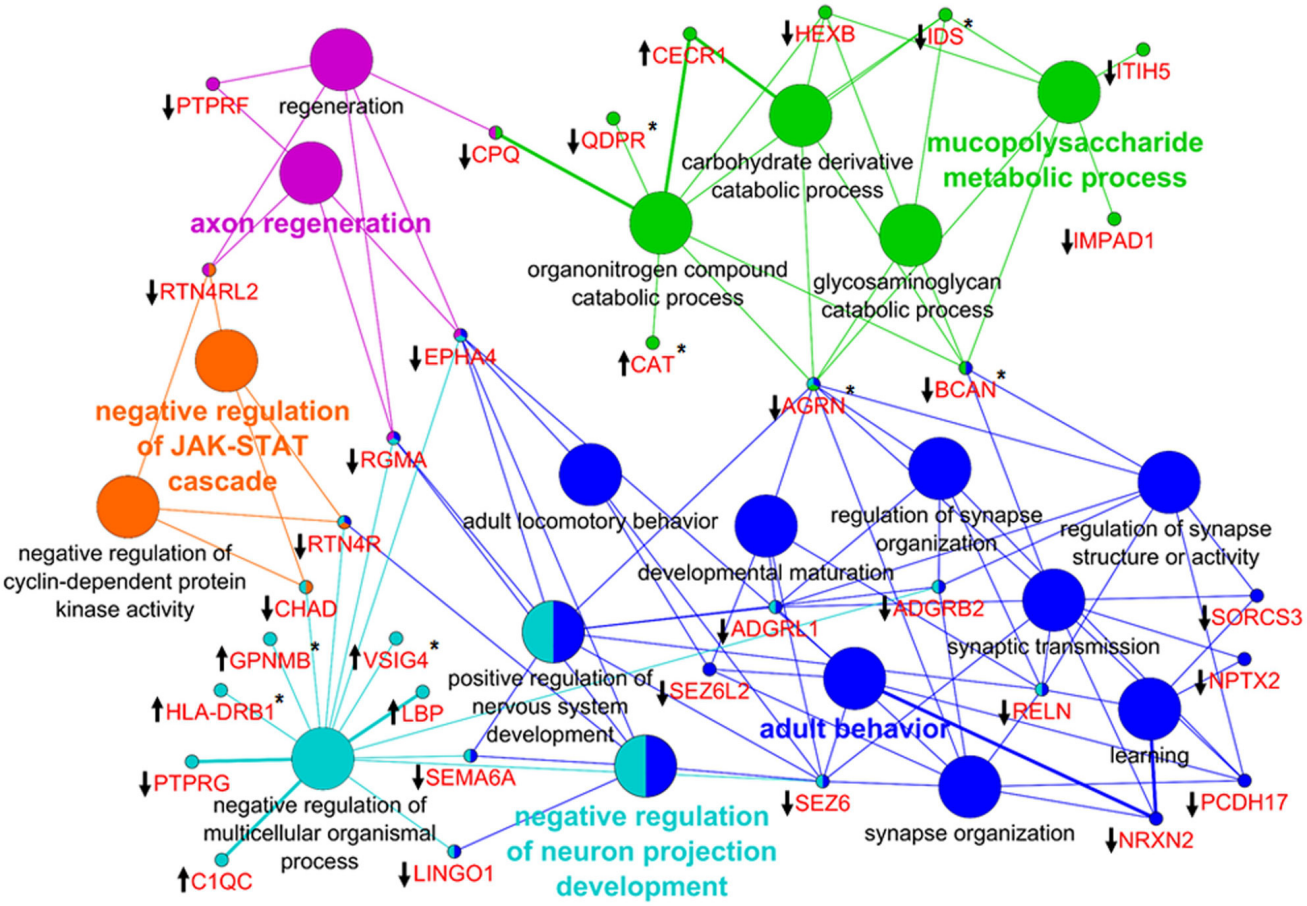
Network enrichment analysis of differentially abundant proteins In total, 66 proteins were used to generate the network of GO biological processes. Thirty-three proteins (50%) were successfully mapped to 21 unique biological processes. The proteins (gene names) associated with the GO terms are highlighted in red. The direction of alteration is indicated by an arrow, and the occurrence at the proteome or mRNA level is indicated by an asterisk. The color code indicates the group affiliation. The term of each group with the highest significance is shown in bold letters.

To explore the interaction of PCNSL with CNS structures and the vasculature, we investigated the origin of the proteomic changes observed in the CSF of PCNSL patients. First, we analyzed whether PCNSL tumor cells contributed directly to the alterations in the CSF proteome. We compared our results with tumor tissue proteome data (930 proteins, unpublished data) and transcritpome data (3773 mRNA transcripts) [24] (Figure 5). Since only proteins that were more abundant in the CSF of PCNSL patients were likely to have originated from PCNSL cells, we considered the 12 candidate proteins that were more abundant (fold-change > 2.0, *p* < 0.05) in this group. Of these 12 candidate proteins, 6 were identified in PCNSL tissue by LC-MS (2 proteins) and/or cDNA-array analysis (6 transcripts). Second, we performed a bioinformatic analysis of all the proteins that differed in abundance between the PCNSL and control proteomes, to reveal the associated biological processes. In a detailed network enrichment analysis of the 66 altered proteins (Figure 6, Supplementary Table 6), 33 proteins (50%) were successfully assigned to 21 significantly enriched (*p* < 0.05) biological processes. Twenty-six assigned proteins (79%) that were more abundant in the control group were mainly involved in CNS-associated processes. In summary, our comparative approach suggested that the highest portion of the observed changes in the proteomic signature of PCNSL CSF likely originated indirectly from the CNS enviroment, rather than from the B-cell lymphoma.

### Protein ecotodomain shedding contributes to changes in the proteomic CSF signature of PCNSL patients

As proteins from the CNS appeared to contribute significantly to the proteomic signature of CSF from PCNSL patients, we investigated whether the differentially abundant proteins were released actively (by protein secretion) or inactively (by tissue leakage) from the tumor tissue or CNS. We first determined whether the altered proteins were released via classical secretion or ectodomain shedding, as both processes are predicable by sequence analysis. UniProt database interrogation of the 66 differentially abundant proteins revealed that 26 proteins (39.4%) contained a signal peptide for classical secretion, while 36 proteins (54.5%) were membrane-associated, of which 30 proteins contained a transmembrane domain and 6 had a glycosylphosphatidylinositol anchor for membrane binding.

**Figure 7:**
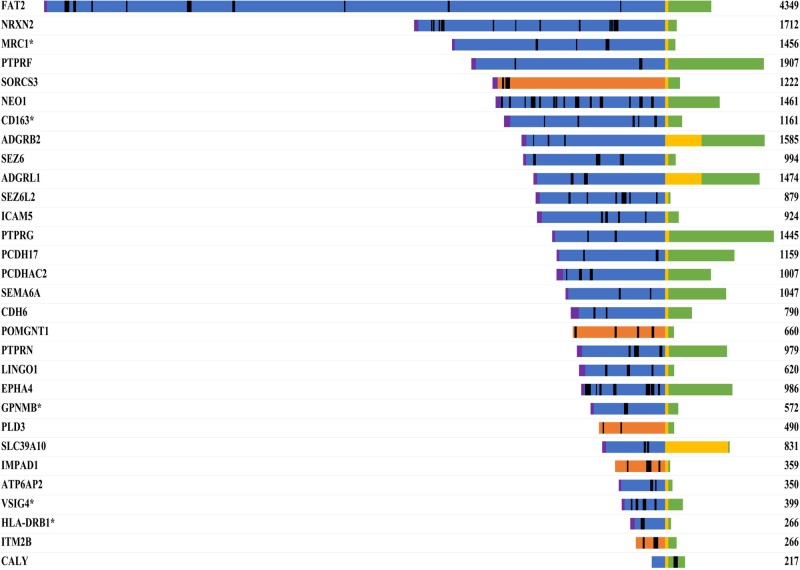
Sequence mapping of identified peptides onto the specific amino acid sequences of transmembrane proteins Proteins that were more abundant in the CSF of PCNSL patients are marked with an asterisk. The number represents the length of the protein (amino acids). Color code: blue, extracellular; yellow, transmembrane region; green, cytoplasmic; orange, luminal; magenta, signal peptide; black, identified peptides.

As 36 of the differentially abundant proteins were membrane-associated, we analyzed whether these proteins were potentially released into the CSF via ectodomain shedding. We mapped the peptides identified by MS for each transmembrane protein onto the specific amino acid sequence (Figure 7). For 29 of the 30 transmembrane proteins, we identified peptides exclusively from the extracellular or luminal domain, suggesting that these proteins may have been released by ectodomain shedding. Our bioinformatic analysis revealed that 62 of the 66 differentially abundant proteins were likely present in the CSF due to classical secretion or ectodomain shedding.

## DISCUSSION

In this study, we characterized the proteomic signature of CSF from PCNSL patients. With our quantitative proteomic approach, we confirmed that PCNSL is associated with BBB dysfunction. Specifically, by comparing the plasma and CSF concentrations of different plasma proteins (albumin, IgG, IgA and IgM), we identified BBB dysfunction in 12 of 17 PCNSL patients. Contamination from unfiltered plasma proteins can hamper the detailed characterization of the CSF proteome [25–27]. By performing correlation analysis with the albumin concentration [27, 28], we identified 127 proteins as contaminants likely to have leaked into CSF due to BBB dysfunction. After excluding these 127 proteins, we obtained the CSF proteome of PCNSL patients, comprising 306 proteins. More than half of the proteins in this proteome (185 proteins, 60%) likely originated in brain tissue, whereas the rest (64 proteins) were assigned to plasma and liver, reflecting that CSF is an ultrafiltrate of blood plasma. Quantitative analysis of the CSF proteome from PCNSL patients and tumor-free controls revealed 66 proteins with differential abundance and 104 proteins with stable abundance between the groups.

In a previous proteome-wide study of PCNSL patients and tumor-free controls, around 500 proteins were identified in the CSF, 76 of which differed in abundance between the groups [29]. The protein data in this publication of Roy and colleagues were not corrected for BBB dysfunction, which likely explains the small overlap of three candidate proteins (C1QC, BCAN, CPQ). Furthermore, we showed for the first time that treatment of PCNSL patients with steroids had no significant impact on the proteomic signature of the CSF at the time of analysis. This observation was surprising, as we expected a massive influence from apoptotic tumor cells upon steroid treatment. Therefore, we speculate that the extended time between treatment and CSF withdrawal diminished the effects of steroid treatment.

Further, by comparing our CSF data with proteome and transcriptome data from whole-cell lysates of PCNSL tissue, we demonstrated that the verifiable direct contribution of tumor cells to the PCNSL CSF proteome was quite low. Only 6 candidate proteins (of the 12 proteins in CSF that were more abundant in PCNSL patients than in controls) were also identified in tumor tissue. However, secreted proteins may be present at low concentrations in tumor tissue but be enriched in the extracellular space. Thus, we cannot exclude the possibility that most of the suggested biomarkers of PCNSL (such as IL-6 [18], IL-10 [19] and CXCL-13 [17]) and soluble receptor proteins (such as sCD27 [15, 21] and sIL-2R [18]) were more abundant in PCNSL patients because they had originated in PCNSL tumor tissue. Our proteomic approach, with a detection limit of 1.5 × 10^−5^ mg/L for CSF analysis, did not allow us to identify such biomarkers in tissues and CSF. Even in large-scale CSF proteome studies, in which protein identification yielded 2630 proteins [10], 2875 proteins [11], 2513 proteins [12] and 2615 proteins [13], the group of cytokines was underrepresented. Despite extensive prefractionation protocols, only sCD27 was identified [11]. This suggests that the limit of detection of the applied LC-MS/MS approach was still too high to identify such protein classes. Thus, targeted approaches such as selected reaction monitoring or antibody-based assays (e.g., enzyme-linked immunosorbent assays) need to be considered.

It is noteworthy that most of the 66 PCNSL-associated proteins could have been actively released, either by classical protein secretion (26 proteins) or by protein ectodomain shedding (36 proteins). Altogether, 36 proteins (55%) with transmembrane domains or glycosylphosphatidylinositol anchors were detected in the CSF, and most of the peptides from transmembrane proteins were from the extracellular or luminal domain, suggesting that PCNSL is associated with altered ectodomain shedding [8, 30]. Twelve of the 36 candidate proteins (Figure 7, Supplementary Table 7) have already been reported to be released by ectodomain shedding, among which CD163 and transmembrane glycoprotein NMB (GPNMB) are known in the context of tumor biology. The shed form of CD163 is upregulated in a large range of inflammatory diseases and Hodgkin lymphoma [31], is induced by anti-inflammatory factors such as IL-6 and IL-10 [32], and is known to be upregulated in PCNSL [18, 19]. CD163 undergoes ectodomain shedding by membrane-bound metalloproteinases of the ADAM family (‘a disintegrin and metalloproteinase;’ e.g., ADAM17), yielding a soluble form (sCD163) of the receptor [33, 34]. The shedding of CD163 can be inhibited by tissue inhibitor of metalloproteinase 3 (TIMP3) [35]. In our study, sCD163 was more abundant in the CSF of PCNSL patients than of control patients, and thus may have been released by tumor cells.

The next candidate protein that has been reported to be upregulated in various cancer types (e.g., melanoma [36, 37], glioma [38] and breast cancer [39]) is GPNMB. GPNMB levels were also found to be higher in CSF from PCNSL patients than from control patients, and therefore likely originated from tumor cells as well. Glioblastoma multiforme patients with increased mRNA and protein levels of GPNMB were reported to have a significantly higher risk of death [38]. Rose and coworkers demonstrated that ADAM10 cleaves GPNMB, releasing a soluble form of the extracellular domain with angiogenic properties [40]. The soluble form of GPNMB produced by ectodomain shedding can induce the expression of matrix metalloproteinase 3 (MMP3) [41], which can shed another transmembrane protein identified in our study, intercellular adhesion molecule 5, releasing a soluble form [42, 43]. Intercellular adhesion molecule 5 was found to be reduced in the CSF of PCNSL patients.

Another candidate protein for ectodomain shedding is human leukocyte antigen (HLA)-DRB1. HLA-DRB1 is part of HLA class II, also known as the major histocompatibility complex (MHC) antigen MHC-II complex. HLA-DRB1 is loaded with peptides derived from antigens that have been endocytosed by antigen-presenting cells. Based on our identification of the ectodomain of HLA-DRB1 in the CSF of PCNSL patients, we suggest an alternative mechanism of cancer-associated immune evasion, which, to our knowledge, has not been described so far. The combined loss of HLA class I and HLA class II antigens is known to occur at a high frequency in PCNSL, thus allowing immune escape [44]. Until now, the loss or aberrant expression of HLA on the cell surface has mostly been linked to cytosolic retention [45] or incorrect loading of the antigen-binding groove [46]. Here, we suggest that HLA class II proteins may be reduced on the cell surface by a proteolytic mechanism, which has been already described for the HLA class I protein HLA-A2 [47]. The ectodomain of HLA-A2 is known to be shed by alpha secretase and further processed by PS1/gamma secretase. Although a vesicular process for MHC II secretion has already been described [48], we speculate that ectodomain shedding may be an alternative process to reduce HLA class II proteins on the cell surface, thus allowing immune evasion.

As we found changes in the abundance of ectodomains from transmembrane proteins in the CSF of PCNSL patients, we suggest that PCNSL tumor cells actively release MMPs or TIMPs, thus altering the shedding of tumor cells or cells in the surrounding CNS environment. Transcriptome data from PCNSL patients [24] support this hypothesis and confirm that members of the ADAM, MMP and TIMP family are broadly expressed in PCNSL tissue. So far, we can only speculate that these shed proteins are involved in PCNSL tumor biology and represent targets for therapeutic interventions.

A combination of different targeted and prefractionation approaches will be necessary to fully exploit the *in vivo* secretome of PCNSL tissue and reveal the tumor-environment interaction in greater detail. Nevertheless, we suggest that PCNSL influences its environment, leading to ectodomain shedding. It appears that only a few proteins originating from tumor tissue are available for future development as biomarkers to distinguish PCNSL from other diseases, and that proteins from the CNS environment must be considered as surrogate markers.

## MATERIALS AND METHODS

### Patients, clinical data and CSF collection

All CSF samples were obtained from the Department of Neurology, Knappschaftskrankenhaus Bochum (Bochum, Germany) with patients’ informed consent. CSF was collected by a standard operation procedure. Briefly, CSF was collected by lumbar puncture at ambient room temperature, and the first 10 drops were discarded to avoid blood contamination. CSF was immediately centrifuged at 500× *g* at 4°C for 10 min to precipitate cell debris. Afterwards, supernatants were aliquoted and stored at 80°C. The whole procedure was performed within 30 min. For the LC-MS study, CSF samples from 27 patients (10 tumor-free controls; 17 PCNSL patients, 7 untreated and 10 treated with steroids) were included (Table 1; for detailed information see Supplementary Table 1A). Patients were selected according to age (control: mean age 67.0 ± 5.5 years; PCNSL: mean age 63.9 ± 9.1 years) and gender (control: 5 male and 5 female; PCNSL: 8 male and 9 female). The concentrations of albumin, IgG, IgA and IgM in serum and CSF were determined by the Knappschaftskrankenhaus Bochum via turbidity measurement (Roche Cobas 6000/Tina-quant, Roche, Mannheim, Germany) or a Nephelometer BN II (Siemens Healthineers, Erlangen, Germany) according to the manufacturers’ instructions.

### Preparation of CSF samples

The CSF protein concentration was determined by a Pierce 660 nm Protein Assay as described in the manufacturer's protocol (Thermo Fisher Scientific, Rockford, IL, USA). For protein digestion, 10 μg protein was diluted to a total volume of 50 μL and mixed with 150 μL SMART digest buffer (Thermo Fisher Scientific, Bremen, Germany). Next, the mixture was transferred to a SMART digest tube containing immobilized trypsin (Thermo Fisher Scientific, Bremen, Germany). Digestion was performed on a tabletop shaker with three consecutive steps: 1200 rpm at 70°C for 60 minutes, 1200 rpm at 80°C for 15 minutes, and 1200 rpm at 90°C for 15 minutes. Proteolysis was stopped by the addition of 200 μL 0.1% trifluoroacetic acid (TFA). For detailed quantification, isotopically labeled (heavy arginine or lysine) synthesized peptides of CD14 (Thermo Fisher Scientific, Ulm, Germany) were spiked into the digested samples in a concentration range of 0.312 to 320 fmol/mL. Subsequently, samples were desalted and purified by solid-phase extraction (SOLAμ HRP SPE Plate, Thermo Fisher Scientific, Bremen, Germany) according to the manufacturer's instructions. The solvent was completely removed with a vacuum concentrator (Eppendorf Concentrator 5301, Eppendorf, Hamburg, Germany) and stored at 80°C. Each sample was reconstituted in 100 μL TFA prior to LC-MS analysis.

### LC-MS analysis of CSF samples

For each LC-MS run, 500 ng sample was analyzed with a nano-high-performance liquid chromatography (HPLC)/ESI-MS system composed of an RSLCnano U3000 HPLC and a QExactive plus mass spectrometer (Thermo Fisher Scientific, Bremen, Germany) equipped with a nano-electrospray ion source. Each sample was loaded onto a trapping column (Acclaim PepMap C_18_, 2 cm × 100 *μ*m × 3 *μ*m particle size, 100 Å pore, Thermo Fisher Scientific, Bremen, Germany) and desalted with 0.1% TFA for 10 min. Peptides were eluted from the trapping column, separated by an analytical column (Acclaim PepMap RSLC C_18_; 25 cm × 75 *μ*m × 2 *μ*m particle size, 100 Å pore; Thermo Fisher Scientific, Bremen, Germany) at a constant flow rate of 300 nL/min for 120 minutes, and sprayed into the MS. The mobile phase for chromatography consisted of 0.1% formic acid in water, and 84% acetonitrile and 0.1% formic acid in water. The parameters for QExactive plus were as follows: positive mode; mass range of 350–2000 *m*/*z* with a resolution of 70.000 (MS1) or 200–2000 *m*/*z* with a resolution of 17.500 (MS2); spray voltage, 1.4 kV; ion transfer tube temperature, 250°C; collision gas, helium; collision gas pressure, 1.3 mTorr; normalized collision energy for MS/MS, 30%; and isolation of +2, +3, +4 monoisotopic precursors with a width of 2.0 Da. TOP10 data-dependent acquisition with activated dynamic exclusion (repeat count 1, duration 100 ms) was applied.

### Identification and quantification of the CSF proteome

For protein identification, Proteome Discoverer (version 2.1.0.81, Thermo Fisher Scientific, Bremen, Germany) and the MS Amanda search engine were considered. MS/MS-spectra were searched against the UniProtKB/Swiss-Prot database (human; including isoforms; date 04/12/2016). The following search parameters were applied: enzyme, trypsin (full); maximum missed cleavage sites, 2; precursor mass tolerance, 10 ppm; fragment mass tolerance, 0.01 Da; and oxidation of M and deamidation of N and Q as dynamic modifications. The false discovery rate was set to 1% (*p* ≤ 0.01). In the case of identified peptides shared among two proteins, these were combined and reported as one protein group. Label-free quantification was performed with Progenesis QI for Proteomics (Version 2.0, Nonlinear Dynamics, Waters Corporation, Newcastle upon Tyne, UK).

For detailed calculation of protein concentrations, we first determined the endogenous concentration of CD14 using isotopically labeled reference peptides (FPAIQNLALR^*^ and LTVGAAQVPAQLLVGALR^*^). Next, we applied the Hi-N method of Progenesis QI for Proteomics (using the two most abundant peptides) to determine the concentrations of proteins in CSF. Briefly, the Hi-N method considers all peptides of a protein for quantification. If more than two peptides are available, Hi-N includes the two most abundant unique (non-conflicting) peptides to calculate the protein concentration. CD14 with a known concentration of 7.9 fmol was applied as single point calibrator, and the signal intensities of the proteins were adjusted to the CD14 concentration for detailed quantification.

### Statistical analysis of protein concentrations

Statistical analysis of protein concentrations from clinical routine analysis (CSF/serum ratios of albumin, IgG, IgA and IgM) was carried out with the Wilcoxon-Mann-Whitney-Test. As a test for blood contamination due to BBB dysfunction, correlation analysis was performed between the concentrations of candidate proteins and CSF albumin (both determined by LC-MS); Pearson correlation was used without any grouping, and *p*-values were adjusted by Benjamini-Hochberg correction. Proteins with a significance threshold of *p* ≤ 0.001 and a positive fold-change (higher abundance in the PCNSL group) were considered as contamination from BBB dysfunction. The stability of protein abundance was determined as described [49]. Therefore, protein abundances were logarithmized (log10), and the mean difference between the groups (‘mean control’ – ‘mean PCNSL’) was calculated for each protein. Afterwards, the ‘two one-sided test’ for equivalence was applied to assess the similarity of the groups (H_1_: |‘mean control’ – ‘mean PCNSL’| < equivalence range (ε)) [49, 50]. The three-fold standard deviation (ε = 0.3) was used as the equivalence range. Proteins with significant *p*-values (*p* ≤ 0.05) were marked as unchanged. For the determination of differentially abundant proteins, LC-MS data were statistically analyzed by analysis of variance, with a *p*-value ≤0.05 and a fold-change > 2.0 as the threshold. Furthermore, only proteins present in 66% of the samples per group and with a minimum of two unique peptides were considered for further analysis.

### Bioinformatic analysis of the CSF proteome

Tissue-specific enrichment analyses were carried out with DAVID and the UniProt tissue annotation database (UP_tissue, date 02/09/2016). The entire identified CSF proteome was used as a background list. Bonferroni correction of *p*-values was applied, and enrichments with *p*-values > 0.05 were discarded.

Network enrichment analysis was performed with Cytoscape environment and a ClueGo plug-in [51]. The following parameters were applied for network analyses: Ontology source, Gene Ontology biological processes (date 02/09/2016); statistical test, enrichment/depletion (two-sided hypergeometric test); *p*-value correction, Benjamini-Hochberg; *p*-value restriction, *p* ≤ 0.01; Gene Ontology (GO) term restriction, ‘min level = 3’, ‘max level = 12’; number of genes, 3; min percentage = 4.0; use GO term fusion; use GO term grouping; GO term connection restriction, kappa score ≥ 0.4; sharing group percentage, 50. The entire identified CSF proteome was used as a reference set.

## SUPPLEMENTARY MATERIALS FIGURES AND TABLES













## Abbreviations

ADAM: a disintegrin and metalloproteinase
BBB: blood-brain barrier
CNS: central nervous system
CSF: cerebrospinal fluid
DLBCL: diffuse large B-cell lymphoma
ESI: electrospray ionization
Extra: extracellular
GO: Gene Ontology
GPI: Glycosylphosphatidylinositol
GPNMB: transmembrane glycoprotein NMB
HLA: human leukocyte antigen
LC-MS: liquid chromatography mass spectrometry
MHC: major histocompatibility complex
MMP: matrix metalloproteinase
MS: mass spectrometry
PCNSL: primary central nervous system lymphoma
Sec: secreted
SP: signal peptide
TFA: trifluoroacetic acid
TIMP: tissue inhibitor of metalloproteinase
TM: transmembrane
