# Supplementary material for: Proteomic changes in cerebrospinal fluid from primary central nervous system lymphoma patients are associated with protein ectodomain shedding
Source: Oncotarget. 2017 Nov 24;8(66):110118–32. doi: 10.18632/oncotarget.22654 (PMC5746369; doi:10.18632/oncotarget.22654)
Supplement: Supplementary file 1 [file oncotarget-08-110118-s001.pdf]

Proteomic changes in cerebrospinal fluid from primary central nervous system lymphoma patients are associated with protein ectodomain shedding

SUPPLEMENTARY MATERIALS

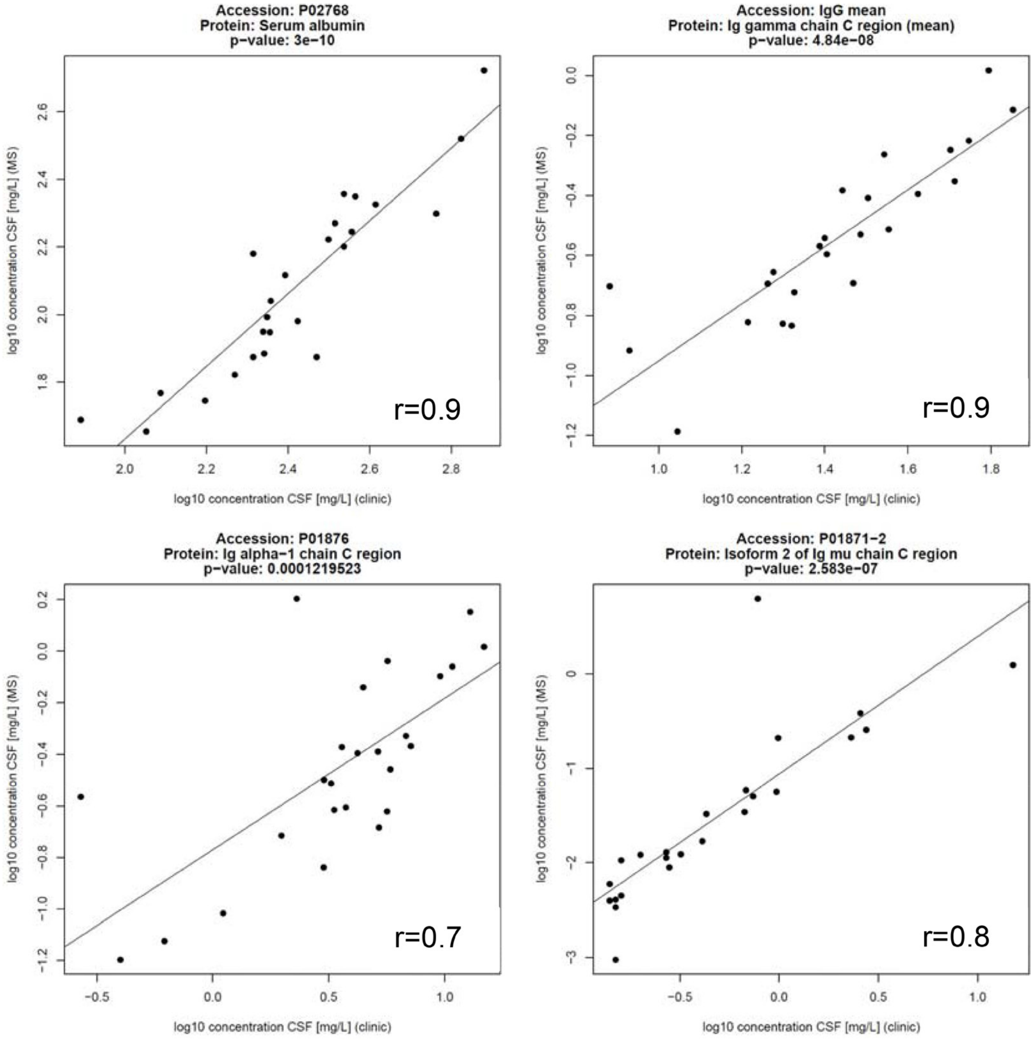

Supplementary Figure 1: Correlation of clinically determined concentrations of albumin, IgG, IgA and IgM with the concentrations obtained by MS. For each protein, the adjusted *p*-value (Benjamini-Hochberg corrected) and Pearson's correlation coefficient (r) are shown.

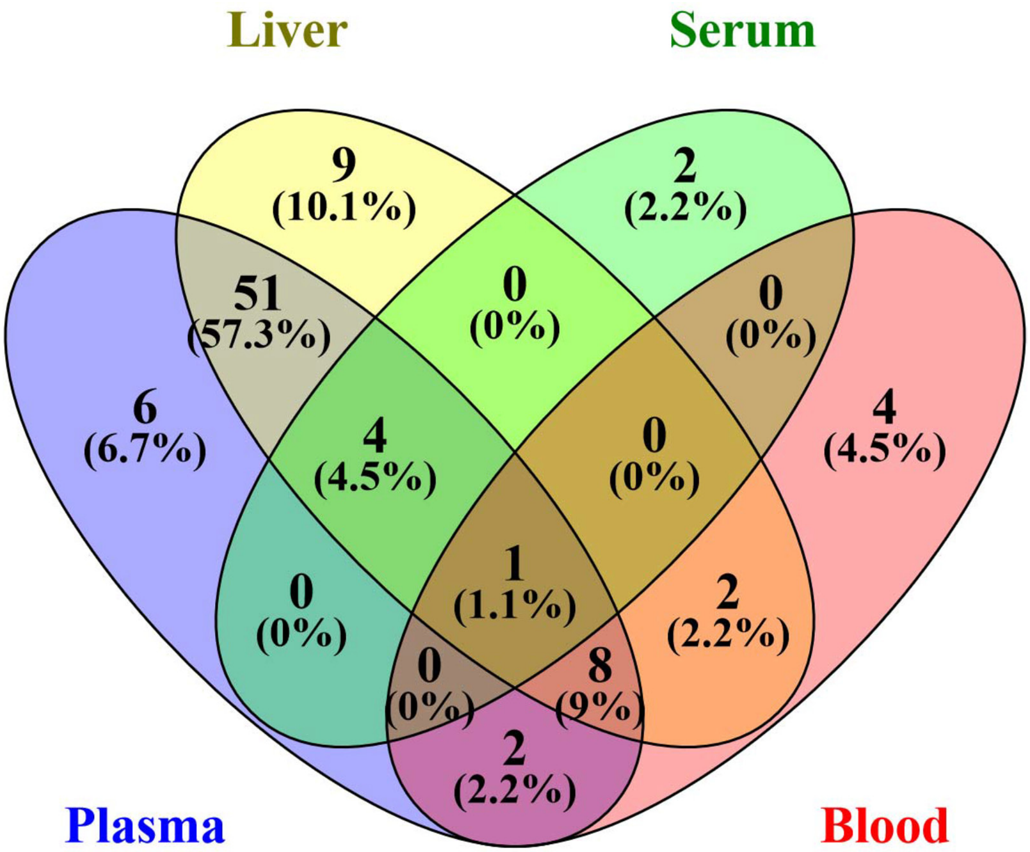

Supplementary Figure 2: Enrichment analysis of proteins significantly correlating (*p* < 0.001) with CSF albumin. In total, 127 proteins were used for enrichment analysis against a tissue-specific database (UniProt tissue database, UP_tissue).

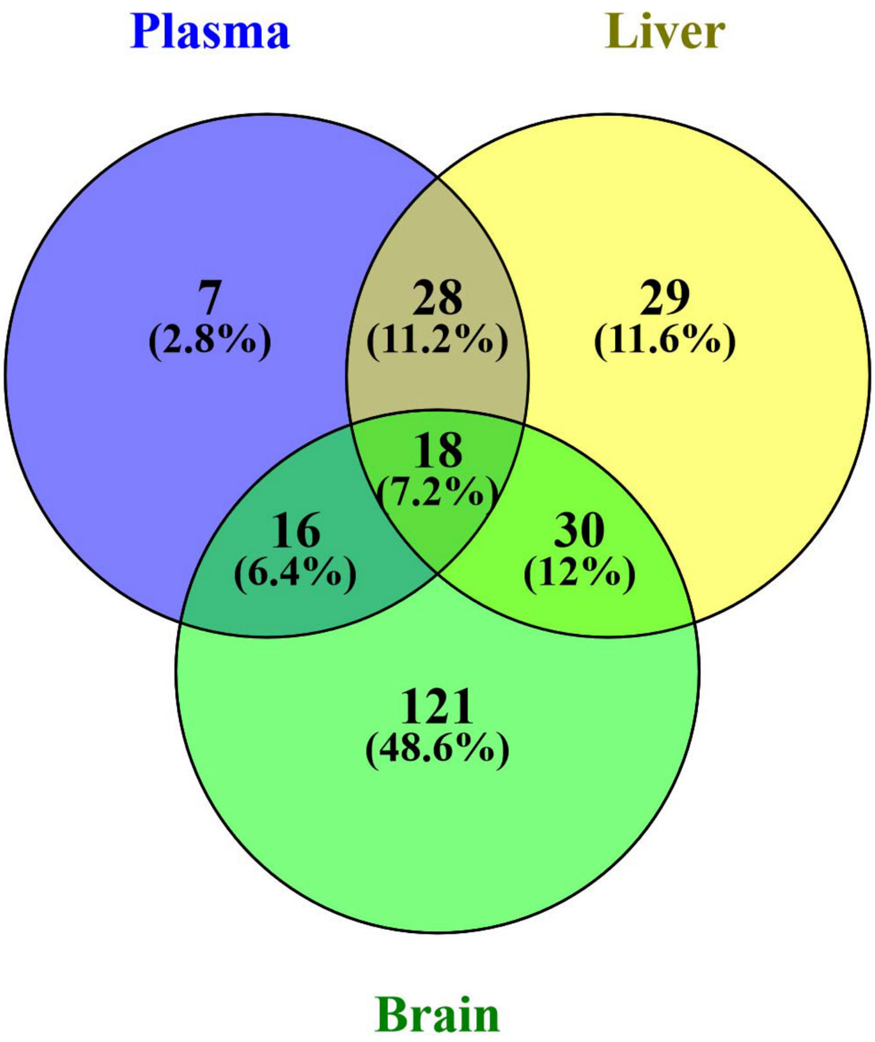

Supplementary Figure 3: Enrichment analysis of proteins not correlating (*p* > 0.001) with CSF albumin. In total, 306 proteins were used for enrichment analysis against a tissue-specific database (UniProt tissue database, UP_tissue).

Supplementary Table 1A: Data of participating individuals

Type	Steroids	Age	Albumin serum [g/L]	Albumin CSF [mg/L]	IgG serum [g/L]	IgG CSF [mg/L]	IgA serum [g/L]	IgA CSF [mg/L]	IgM serum [g/L]	IgM CSF [mg/L]	Diagnosis
Control 1	no	71		78.0		8.5		0.6		0.2	Dementia (Alzheimer's disease)
Control 2	no	62	33.8	219.6	7.6	18.9	2.4	3.2	0.4	0.2	Pseudodemence
Control 3	no	72	35.7	327.6	8.9	27.7	3.2	4.5	0.6	0.3	Headache
Control 4	no	72	33.7	122.4	5.7	11.1	0.7	0.4	0.9	0.3	Dementia (Alzheimer's disease)
Control 5	no	65	34.5	218.4	13.3	42.1	4.3	6.8	1.4	0.3	Incomplete oculomotor palsy
Control 6	no	66	39.3	226.8	9.9	25.4	2.1	5.2	0.7	0.2	Headache
Control 7	no	73	30.9	157.2	13.9	30.6	4.3	5.2	0.8	0.1	Recurrent loss of consciousness/Postural orthostatic tachycardia syndrome
Control 8	no	54	36.3	223.2	5.9	7.6	0.4	0.3	0.4	0.1	Discreet sensitive polyneuropathy
Control 9	no	69	35.4	228.0	8.3	18.3	1.5	2.0	1.0	0.2	Pseudodemence
Control 10	no	66		206.4		29.4		3.8		0.2	Primary progressive aphasia
PCNSL 1	no	74	31.1	344.4	7.1	34.9	1.2	3.6	0.6	2.8	PCNSL (initial diagnosis, without steroids, before therapy)
PCNSL 2	no	73	34.8	112.8	11.6	19.9	1.9	1.1	1.6	0.2	PCNSL (initial diagnosis, without steroids, before therapy)
PCNSL 3	no	65	36.5	295.2	7.3	21.2	3.4	5.8	0.9	0.7	PCNSL (initial diagnosis, without steroids, before therapy)
PCNSL 4	no	70	42.2	265.2		16.4	1.2	3.3	0.6	0.3	PCNSL (initial diagnosis, without steroids, before therapy)
PCNSL 5	no	70	32.5	367.2	11.2	62.3	4.1	2.3	1.3	2.6	PCNSL (initial diagnosis, without steroids, before therapy)
PCNSL 6	no	58	38.8	759.6	5.5		1.7	12.9	0.6		PCNSL (initial diagnosis, without steroids, before therapy)
PCNSL 7	no	44	30.6	344.4	3.2	35.8	1.1	4.2	1.2	1.0	PCNSL (initial diagnosis, without steroids, before therapy)
PCNSL 8	yes	79	26.3	206.4	5.1	25.1	2.6	5.7	3.3	1.0	PCNSL (initial diagnosis, with steroids, before therapy)
PCNSL 9	yes	54	33.6	579.6	6.1	51.6	0.8	5.7	1.2	2.3	PCNSL (initial diagnosis, with steroids, before therapy)
PCNSL 10	yes	78	36.7	411.6	8.8	55.8	2.9	14.8	0.7	0.4	PCNSL (initial diagnosis, with steroids, before therapy)
PCNSL 11	yes	60	39.2	360.0	6.5	31.9	1.2	3.0	1.1	0.8	PCNSL (initial diagnosis, with steroids, before therapy)
PCNSL 12	yes	63	32.3	247.2	5.3	24.4	3.3	9.6	0.3	0.7	PCNSL (initial diagnosis, with steroids, before therapy)
PCNSL 13	yes	57									PCNSL (initial diagnosis, with steroids, before therapy)
PCNSL 14	yes	55	32.1	186.0	7.6	20.9	1.6	3.0	1.7	0.4	PCNSL (initial diagnosis, with steroids, before therapy)
PCNSL 15	yes	58	33.3	315.6	8.0	50.4	3.3	10.8	1.2	0.7	PCNSL (initial diagnosis, with steroids, before therapy)
PCNSL 16	yes	66	30.3	667.2	5.8	71.3	0.8	7.2	4.5	15.1	PCNSL (initial diagnosis, with steroids, before therapy)
PCNSL 17	yes	62									PCNSL (initial diagnosis, with steroids, before therapy)

Supplementary Table 1B: Blood-brain barrier dysfunction: CSF/serum quotients of serum albumin, IgG, IgA and IgM

Type	Steroids	Age	QAlb	QIgG	QIgA	QIgM	Comment
Control 1	no	71					insufficient data
Control 2	no	62	6.5	2.5	1.4	0.4	
Control 3	no	72	9.2	3.1	1.4	0.5	BBB dysfunction
Control 4	no	72	3.6	2.0	0.6	0.3	
Control 5	no	65	6.3	3.2	1.6	0.2	
Control 6	no	66	5.8	2.6	2.5	0.2	
Control 7	no	73	5.1	2.2	1.2	0.2	
Control 8	no	54	6.1	1.3	0.6	0.3	
Control 9	no	69	6.4	2.2	1.3	0.2	
Control 10	no	66					insufficient data
PCNSL 1	no	74	11.1	4.9	3.0	4.6	BBB dysfunction
PCNSL 2	no	73	3.2	1.7	0.6	0.1	
PCNSL 3	no	65	8.1	2.9	1.7	0.8	
PCNSL 4	no	70	6.3		2.8	0.5	
PCNSL 5	no	70	11.3	5.6	0.6	2.0	BBB dysfunction
PCNSL 6	no	58	19.6		7.6		BBB dysfunction
PCNSL 7	no	44	11.3	11.2	3.8	0.8	BBB dysfunction
PCNSL 8	yes	79	7.8	4.9	2.2	0.3	
PCNSL 9	yes	54	17.3	8.4	6.8	1.9	BBB dysfunction
PCNSL 10	yes	78	11.2	6.4	5.1	0.6	BBB dysfunction
PCNSL 11	yes	60	9.2	4.9	2.5	0.7	BBB dysfunction
PCNSL 12	yes	63	7.7	4.6	2.9	2.3	
PCNSL 13	yes	57					insufficient data
PCNSL 14	yes	55	5.8	2.8	1.9	0.2	
PCNSL 15	yes	58	9.5	6.3	3.3	0.6	BBB dysfunction
PCNSL 16	yes	66	22.0	12.3	9.4	3.3	BBB dysfunction
PCNSL 17	yes	62					insufficient data

Supplementary Table 2: Serum albumin associated proteins. See Supplementary_Table_2

Supplementary Table 3: Classified PCNSL CSF proteome (306 proteins). See Supplementary_ Table_3

Supplementary Table 4: CSF core proteome. See Supplementary_Table_4

Supplementary Table 5: Differential analysis of non-albumin correlated proteins. See Supplementary_Table_5

Supplementary Table 6: Network enrichment analysis of 66 significantly altered proteins. See Supplementary_Table_6

Supplementary Table 7: Published membrane proteins which undergo ectodomain shedding

Protein	Comment	Literature
CD163	Shed by ADAM17 forming soluble sCD163	Droste et al., 1999; Etzerodt et al., 2014; Matsushita et al., 2002 [2, 3, 10]
GPNMB	ADAM10 cleaves GPNMB releasing a soluble form of the extracellular domain	Rose et al., 2010 [13]
ICAM5	MMP3 sheds ICAM5 forming a soluble form	Conant et al., 2010; Niedringhaus et al., 2012 [1, 11]
PTPRF	PTPRF is a potential substrate of ADAM17	Ruhe et al., 2006 [14]
PTPRG	Possibly shed by ADAM-family members forming a soluble extracellular domain	Vezzalini et al., 2007; Primo et al., 2008 [12, 17]
PTPRN	Possibly shed by ADAM-family members forming a soluble extracellular domain	Vezzalini et al., 2007; Primo et al., 2008 [12, 17]
SORCS3	Shed by ADAM17 forming soluble SORCS3	Hermey et al., 2006 [5]
ITM2B	ITM2B is first cleaved by furin and than shed by ADAM10 forming BRI2C	Kim et al., 2008; Martin et al., 2009; Tsachaki et al., 2011 [6, 9, 16]
SEZ6L2	Shed by BACE2 forming soluble SEZ6L2	Stutzer et al., 2013 [15]
SEZ6	Shed by BACE1 forming soulbe SEZ6	Kuhn et al., 2012 [8]
EPHA4	Soluble form identified	Gatto et al., 2014 [4]
ATP6AP2	Furin sheds ATP6AP2 forming a soluble form	Kinouchi et al., 2013 [7]

REFERENCES

1. Conant K, Wang Y, Szklarczyk A, Dudak A, Mattson MP, Lim ST. Matrix metalloproteinase-dependent shedding of intercellular adhesion molecule-5 occurs with long-term potentiation. Neuroscience. 2010; 166:508–521. https://doi.org/10.1016/j.neuroscience.2009.12.061.
2. Droste A, Sorg C, Hogger P. Shedding of CD163, a novel regulatory mechanism for a member of the scavenger receptor cysteine-rich family. Biochem Biophys Res Comm. 1999; 256:110-113. https://doi.org/10.1006/bbrc.1999.0294.
3. Etzerodt A, Rasmussen MR, Svendsen P, Chalaris A, Schwarz J, Galea I, Møller HJ, Moestrup SK. Structural basis for inflammation-driven shedding of CD163 ectodomain and tumor necrosis factor-alpha in macrophages. J Biol Chem. 2014; 289:778-788. https://doi.org/10.1074/jbc.M113.520213.
4. Gatto G, Morales D, Kania A, Klein R. EphA4 receptor shedding regulates spinal motor axon guidance. Curr Biol. 2014; 24:2355–2365. https://doi.org/10.1016/j.cub.2014.08.028.
5. Hermey G, Sjogaard SS, Petersen CM, Nykjaer A, Gliemann J. Tumour necrosis factor alpha-converting enzyme mediates ectodomain shedding of Vps10p-domain receptor family members. Biochem J. 2006; 395:285–293. https://doi.org/10.1042/BJ20051364.
6. Kim J, Miller VM, Levites Y, West KJ, Zwizinski CW, Moore BD, Troendle FJ, Bann M, Verbeeck C, Price RW, Smithson L, Sonoda L, Wagg K, et al. BRI2 (ITM2b) inhibits Abeta deposition *in vivo*. J Neurosci. 2008; 28:6030–6036. https://doi.org/10.1523/JNEUROSCI.0891-08.2008.
7. Kinouchi K, Ichihara A, Sano M, Sun-Wada GH, Wada Y, Ochi H, Fukuda T, Bokuda K, Kurosawa H, Yoshida N, Takeda S, Fukuda K, Itoh H. The role of individual domains and the significance of shedding of ATP6AP2/(pro)renin receptor in vacuolar H(+)-ATPase biogenesis. PLoS One. 2013; 8:e78603. https://doi.org/10.1371/journal.pone.0078603.
8. Kuhn PH, Koroniak K, Hogl S, Colombo A, Zeitschel U, Willem M, Volbracht C, Schepers U, Imhof A, Hoffmeister A, Haass C, Roßner S, Bräse S, Lichtenthaler SF. Secretome protein enrichment identifies physiological BACE1 protease substrates in neurons. EMBO J. 2012; 31:3157–3168. https://doi.org/10.1038/emboj.2012.173.
9. Martin L, Fluhrer R, Haass C. Substrate requirements for SPPL2b-dependent regulated intramembrane proteolysis. J Biol Chem. 2009; 284:5662–5670. https://doi.org/10.1074/jbc.M807485200.
10. Matsushita N, Kashiwagi M, Wait R, Nagayoshi R, Nakamura M, Matsuda T, Hogger P, Guyre PM, Nagase H, Matsuyama T. Elevated levels of soluble CD163 in sera and fluids from rheumatoid arthritis patients and inhibition of the shedding of CD163 by TIMP-3. Clin Exp Immunol. 2002; 130:156–161.
11. Niedringhaus M, Chen X, Dzakpasu R, Conant K. MMPs and soluble ICAM-5 increase neuronal excitability within *in vitro* networks of hippocampal neurons. PLoS One. 2012; 7:e42631. https://doi.org/10.1371/journal.pone.0042631.
12. Primo ME, Klinke S, Sica MP, Goldbaum FA, Jakoncic J, Poskus E, Ermácora MR. Structure of the mature ectodomain of the human receptor-type protein-tyrosine phosphatase IA-2. J Biol Chem. 2008; 283:4674–4681. https://doi.org/10.1074/jbc.M708144200.
13. Rose AA, Annis MG, Dong Z, Pepin F, Hallett M, Park M, Siegel PM. ADAM10 releases a soluble form of the GPNMB/Osteoactivin extracellular domain with angiogenic properties. PLoS One. 2010; 5:e12093. Https://doi.org/10.1371/journal.pone.0012093.

14. Ruhe JE, Streit S, Hart S, Ullrich A. EGFR signaling leads to downregulation of PTP-LAR via TACE-mediated proteolytic processing. Cell Sig. 2006; 18:1515–1527. https://doi.org/10.1016/j.cellsig.2005.12.003.

15. Stutzer I, Selevsek N, Esterhazy D, Schmidt A, Aebersold R, Stoffel M. Systematic proteomic analysis identifies beta-site amyloid precursor protein cleaving enzyme 2 and 1 (BACE2 and BACE1) substrates in pancreatic beta-cells. J Biol Chem. 2013; 288:10536–10547. https://doi.org/10.1074/jbc.M112.444703.

16. Tsachaki M, Serlidaki D, Fetani A, Zarkou V, Rozani I, Ghiso J, Efthimiopoulos S. Glycosylation of BRI2 on asparagine 170 is involved in its trafficking to the cell surface but not in its processing by furin or ADAM10. Glycobiology. 2011; 21:1382–1388. https://doi.org/10.1093/glycob/cwr097.

17. Vezzalini M, Mombello A, Menestrina F, Mafficini A, Della Peruta M, van Niekerk C, Barbareschi M, Scarpa A, Sorio C. Expression of transmembrane protein tyrosine phosphatase gamma (PTPgamma) in normal and neoplastic human tissues. Histopathology. 2007; 50:615–628. https://doi.org/10.1111/j.1365-1559.2007.02661.x.
