# Supplementary material for: Proteomic changes in cerebrospinal fluid from primary central nervous system lymphoma patients are associated with protein ectodomain shedding
Source: Oncotarget. 2017 Nov 24;8(66):110118–32. doi: 10.18632/oncotarget.22654 (PMC5746369; doi:10.18632/oncotarget.22654)
Supplement: Supplementary file 2 [file oncotarget-08-110118-s002.docx]

**Supplementary Table 2: Serum albumin associated proteins. Correlation analysis of CSF proteins with CSF serum albumin.**

| **UniProt Accession** | **Gene** | **Protein Name** | **log(fold change)** | **p-Value** | **r²** |
| --- | --- | --- | --- | --- | --- |
| P19823 | ITIH2 | Inter-alpha-trypsin inhibitor heavy chain H2 (ITI heavy chain H2) (ITI-HC2) (Inter-alpha-inhibitor heavy chain 2) (Inter-alpha-trypsin inhibitor complex component II) (Serum-derived hyaluronan-associated protein) (SHAP) | 1.56 | 2.26E-14 | 0.9 |
| P00748 | F12 | Coagulation factor XII (EC 3.4.21.38) (Hageman factor) (HAF) [Cleaved into: Coagulation factor XIIa heavy chain; Beta-factor XIIa part 1; Coagulation factor XIIa light chain (Beta-factor XIIa part 2)] | 1.18 | 1.13E-13 | 0.9 |
| P19827 | ITIH1 | Inter-alpha-trypsin inhibitor heavy chain H1 (ITI heavy chain H1) (ITI-HC1) (Inter-alpha-inhibitor heavy chain 1) (Inter-alpha-trypsin inhibitor complex component III) (Serum-derived hyaluronan-associated protein) (SHAP) | 1.52 | 6.34E-13 | 0.9 |
| P08697 | SERPINF2 | Alpha-2-antiplasmin (Alpha-2-AP) (Alpha-2-plasmin inhibitor) (Alpha-2-PI) (Serpin F2) | 0.96 | 6.48E-13 | 0.9 |
| P02647 | APOA1 | Apolipoprotein A-I (Apo-AI) (ApoA-I) (Apolipoprotein A1) [Cleaved into: Proapolipoprotein A-I (ProapoA-I); Truncated apolipoprotein A-I (Apolipoprotein A-I(1-242))] | 1.29 | 2.65E-12 | 0.9 |
| P01042-2 | KNG1 | Kininogen-1 (Alpha-2-thiol proteinase inhibitor) (Fitzgerald factor) (High molecular weight kininogen) (HMWK) (Williams-Fitzgerald-Flaujeac factor) [Cleaved into: Kininogen-1 heavy chain; T-kinin (Ile-Ser-Bradykinin); Bradykinin (Kallidin I); Lysyl-bradykinin (Kallidin II); Kininogen-1 light chain; Low molecular weight growth-promoting factor] | 1.07 | 2.65E-12 | 0.9 |
| P04217 | A1BG | Alpha-1B-glycoprotein (Alpha-1-B glycoprotein) | 0.88 | 4.48E-12 | 0.9 |
| P43652 | AFM | Afamin (Alpha-albumin) (Alpha-Alb) | 1.33 | 2.86E-11 | 0.9 |
| P02652 | APOA2 | Apolipoprotein A-II (Apo-AII) (ApoA-II) (Apolipoprotein A2) [Cleaved into: Proapolipoprotein A-II (ProapoA-II); Truncated apolipoprotein A-II (Apolipoprotein A-II(1-76))] | 1.38 | 3.08E-11 | 0.9 |
| P00747 | PLG | Plasminogen (EC 3.4.21.7) [Cleaved into: Plasmin heavy chain A; Activation peptide; Angiostatin; Plasmin heavy chain A, short form; Plasmin light chain B] | 1.09 | 5.96E-11 | 0.9 |
| O95445 | APOM | Apolipoprotein M (Apo-M) (ApoM) (Protein G3a) | 1.44 | 6.99E-11 | 0.9 |
| P01024 | C3 | Complement C3 (C3 and PZP-like alpha-2-macroglobulin domain-containing protein 1) [Cleaved into: Complement C3 beta chain; C3-beta-c (C3bc); Complement C3 alpha chain; C3a anaphylatoxin; Acylation stimulating protein (ASP) (C3adesArg); Complement C3b alpha' chain; Complement C3c alpha' chain fragment 1; Complement C3dg fragment; Complement C3g fragment; Complement C3d fragment; Complement C3f fragment; Complement C3c alpha' chain fragment 2] | 0.88 | 6.99E-11 | 0.9 |
| P01717 | IGLV3-25 | Immunoglobulin lambda variable 3-25 (Ig lambda chain V-IV region Hil) | 1.35 | 1.00E-10 | 0.9 |
| P02655 | APOC2 | Apolipoprotein C-II (Apo-CII) (ApoC-II) (Apolipoprotein C2) [Cleaved into: Proapolipoprotein C-II (ProapoC-II)] | 1.28 | 1.38E-10 | 0.9 |
| Q14624 | ITIH4 | Inter-alpha-trypsin inhibitor heavy chain H4 (ITI heavy chain H4) (ITI-HC4) (Inter-alpha-inhibitor heavy chain 4) (Inter-alpha-trypsin inhibitor family heavy chain-related protein) (IHRP) (Plasma kallikrein sensitive glycoprotein 120) (Gp120) (PK-120) [Cleaved into: 70 kDa inter-alpha-trypsin inhibitor heavy chain H4; 35 kDa inter-alpha-trypsin inhibitor heavy chain H4] | 1.19 | 2.01E-10 | 0.9 |
| P01625 | IGKV4-1 | Immunoglobulin kappa variable 4-1 | 0.92 | 2.17E-10 | 0.9 |
| P04004 | VTN | Vitronectin (VN) (S-protein) (Serum-spreading factor) (V75) [Cleaved into: Vitronectin V65 subunit; Vitronectin V10 subunit; Somatomedin-B] | 1.17 | 2.66E-10 | 0.8 |
| P02790 | HPX | Hemopexin (Beta-1B-glycoprotein) | 1.03 | 3.67E-10 | 0.8 |
| P32004 | L1CAM | Neural cell adhesion molecule L1 (N-CAM-L1) (NCAM-L1) (CD antigen CD171) | 0.71 | 3.67E-10 | 0.8 |
| P05543 | SERPINA7 | Thyroxine-binding globulin (Serpin A7) (T4-binding globulin) | 1.05 | 3.84E-10 | 0.8 |
| P00450 | CP | Ceruloplasmin (EC 1.16.3.1) (Ferroxidase) | 0.95 | 3.90E-10 | 0.8 |
| P19652 | ORM2 | Alpha-1-acid glycoprotein 2 (AGP 2) (Orosomucoid-2) (OMD 2) | 0.94 | 5.00E-10 | 0.8 |
| P02774-3 | GC | Vitamin D-binding protein (DBP) (VDB) (Gc protein-derived macrophage activating factor) (Gc-MAF) (GcMAF) (Gc-globulin) (Group-specific component) (Gc) (Vitamin D-binding protein-macrophage activating factor) (DBP-maf) | 0.80 | 7.53E-10 | 0.8 |
| Q96IY4 | CPB2 | Carboxypeptidase B2 (EC 3.4.17.20) (Carboxypeptidase U) (CPU) (Plasma carboxypeptidase B) (pCPB) (Thrombin-activable fibrinolysis inhibitor) (TAFI) | 1.29 | 7.82E-10 | 0.8 |
| P01610 | IGKV1-17 | Immunoglobulin kappa variable 1-17 | 0.95 | 1.96E-09 | 0.8 |
| P02654 | APOC1 | Apolipoprotein C-I (Apo-CI) (ApoC-I) (Apolipoprotein C1) [Cleaved into: Truncated apolipoprotein C-I] | 1.33 | 2.39E-09 | 0.8 |
| Q96PD5-2 | PGLYRP2 | N-acetylmuramoyl-L-alanine amidase (EC 3.5.1.28) (Peptidoglycan recognition protein 2) (Peptidoglycan recognition protein long) (PGRP-L) | 0.60 | 2.39E-09 | 0.8 |
| P01857 | IGHG1 | Immunoglobulin heavy constant gamma 1 (Ig gamma-1 chain C region) (Ig gamma-1 chain C region EU) (Ig gamma-1 chain C region KOL) (Ig gamma-1 chain C region NIE) | 0.95 | 2.43E-09 | 0.8 |
| P07357 | C8A | Complement component C8 alpha chain (Complement component 8 subunit alpha) | 0.93 | 3.11E-09 | 0.8 |
| P01622 | IGLC2 | Immunoglobulin lambda constant 2 | 0.74 | 3.60E-09 | 0.8 |
| P13671 | C6 | Complement component C6 | 0.90 | 3.71E-09 | 0.8 |
| P80748 | IGLV3-21 | Immunoglobulin lambda variable 3-21 (Ig lambda chain V-III region LOI) (Ig lambda chain V-V region DEL) (Ig lambda chain V-VII region MOT) | 1.02 | 4.08E-09 | 0.8 |
| P27169 | PON1 | Serum paraoxonase/arylesterase 1 (PON 1) (EC 3.1.1.2) (EC 3.1.1.81) (EC 3.1.8.1) (Aromatic esterase 1) (A-esterase 1) (K-45) (Serum aryldialkylphosphatase 1) | 1.49 | 4.72E-09 | 0.8 |
| P01031 | C5 | Complement C5 (C3 and PZP-like alpha-2-macroglobulin domain-containing protein 4) [Cleaved into: Complement C5 beta chain; Complement C5 alpha chain; C5a anaphylatoxin; Complement C5 alpha' chain] | 1.08 | 5.95E-09 | 0.8 |
| P06681 | C2 | Complement C2 (EC 3.4.21.43) (C3/C5 convertase) [Cleaved into: Complement C2b fragment; Complement C2a fragment] | 1.03 | 6.78E-09 | 0.8 |
| P01764 | IGHV3-23 | Immunoglobulin heavy variable 3-23 (Ig heavy chain V-III region LAY) (Ig heavy chain V-III region POM) (Ig heavy chain V-III region TEI) (Ig heavy chain V-III region TIL) (Ig heavy chain V-III region TUR) (Ig heavy chain V-III region VH26) (Ig heavy chain V-III region WAS) (Ig heavy chain V-III region ZAP) | 0.49 | 8.90E-09 | 0.8 |
| P05156 | CFI | Complement factor I (EC 3.4.21.45) (C3B/C4B inactivator) [Cleaved into: Complement factor I heavy chain; Complement factor I light chain] | 0.77 | 9.43E-09 | 0.8 |
| P01834 | IGKC | Immunoglobulin kappa constant (Ig kappa chain C region) (Ig kappa chain C region AG) (Ig kappa chain C region CUM) (Ig kappa chain C region EU) (Ig kappa chain C region OU) (Ig kappa chain C region ROY) (Ig kappa chain C region TI) | 1.05 | 9.43E-09 | 0.8 |
| P02766 | TTR | Transthyretin (ATTR) (Prealbumin) (TBPA) | 0.98 | 9.43E-09 | 0.8 |
| P0CG05 | IGLC3 | Immunoglobulin lambda constant 3 | 0.91 | 1.03E-08 | 0.8 |
| P01611 | IGKV1D-12 | Immunoglobulin kappa variable 1D-12 (Ig kappa chain V-I region Wes) | 0.72 | 1.19E-08 | 0.8 |
| P04208 | IGLV1-47 | Immunoglobulin lambda variable 1-47 | 0.99 | 1.60E-08 | 0.8 |
| P02749 | APOH | Beta-2-glycoprotein 1 (APC inhibitor) (Activated protein C-binding protein) (Anticardiolipin cofactor) (Apolipoprotein H) (Apo-H) (Beta-2-glycoprotein I) (B2GPI) (Beta(2)GPI) | 1.04 | 1.85E-08 | 0.8 |
| P29622 | SERPINA4 | Kallistatin (Kallikrein inhibitor) (Peptidase inhibitor 4) (PI-4) (Serpin A4) | 0.99 | 1.85E-08 | 0.8 |
| P08603 | CFH | Complement factor H (H factor 1) | 0.76 | 2.48E-08 | 0.8 |
| P04196 | HRG | Histidine-rich glycoprotein (Histidine-proline-rich glycoprotein) (HPRG) | 0.46 | 2.51E-08 | 0.8 |
| Q9NZP8 | C1RL | Complement C1r subcomponent-like protein (C1r-LP) (C1r-like protein) (EC 3.4.21.-) (C1r-like serine protease analog protein) (CLSPa) | 0.97 | 2.59E-08 | 0.8 |
| P00751 | CFB | Complement factor B (EC 3.4.21.47) (C3/C5 convertase) (Glycine-rich beta glycoprotein) (GBG) (PBF2) (Properdin factor B) [Cleaved into: Complement factor B Ba fragment; Complement factor B Bb fragment] | 0.56 | 2.59E-08 | 0.8 |
| P06309 | IGKV2D-28 | Immunoglobulin kappa variable 2D-28 | 1.08 | 2.69E-08 | 0.8 |
| P05090 | APOD | Apolipoprotein D (Apo-D) (ApoD) | 0.73 | 3.45E-08 | 0.8 |
| P01591 | JCHAIN | Immunoglobulin J chain (Joining chain of multimeric IgA and IgM) | 3.47 | 4.05E-08 | 0.8 |
| P07477 | PRSS1 | Trypsin-1 (EC 3.4.21.4) (Beta-trypsin) (Cationic trypsinogen) (Serine protease 1) (Trypsin I) [Cleaved into: Alpha-trypsin chain 1; Alpha-trypsin chain 2] | 0.69 | 4.58E-08 | 0.7 |
| P02656 | APOC3 | Apolipoprotein C-III (Apo-CIII) (ApoC-III) (Apolipoprotein C3) | 1.48 | 6.41E-08 | 0.7 |
| P08185 | SERPINA6 | Corticosteroid-binding globulin (CBG) (Serpin A6) (Transcortin) | 1.04 | 6.41E-08 | 0.7 |
| P08294 | SOD3 | Extracellular superoxide dismutase [Cu-Zn] (EC-SOD) (EC 1.15.1.1) | 0.52 | 6.41E-08 | 0.7 |
| P03952 | KLKB1 | Plasma kallikrein (EC 3.4.21.34) (Fletcher factor) (Kininogenin) (Plasma prekallikrein) (PKK) [Cleaved into: Plasma kallikrein heavy chain; Plasma kallikrein light chain] | 0.90 | 6.83E-08 | 0.7 |
| P07360 | C8G | Complement component C8 gamma chain | 0.64 | 8.21E-08 | 0.7 |
| P00746 | CFD | Complement factor D (EC 3.4.21.46) (Adipsin) (C3 convertase activator) (Properdin factor D) | 0.57 | 9.64E-08 | 0.7 |
| P02760 | AMBP | Protein AMBP [Cleaved into: Alpha-1-microglobulin (Protein HC) (Alpha-1 microglycoprotein) (Complex-forming glycoprotein heterogeneous in charge); Inter-alpha-trypsin inhibitor light chain (ITI-LC) (Bikunin) (EDC1) (HI-30) (Uronic-acid-rich protein); Trypstatin] | 0.54 | 1.14E-07 | 0.7 |
| P00734 | F2 | Prothrombin (EC 3.4.21.5) (Coagulation factor II) [Cleaved into: Activation peptide fragment 1; Activation peptide fragment 2; Thrombin light chain; Thrombin heavy chain] | 1.14 | 1.57E-07 | 0.7 |
| B9A064 | IGLL5 | Immunoglobulin lambda-like polypeptide 5 (G lambda-1) (Germline immunoglobulin lambda 1) | 0.76 | 1.78E-07 | 0.7 |
| P01859 | IGHG2 | Immunoglobulin heavy constant gamma 2 (Ig gamma-2 chain C region) (Ig gamma-2 chain C region DOT) (Ig gamma-2 chain C region TIL) (Ig gamma-2 chain C region ZIE) | 0.95 | 2.09E-07 | 0.7 |
| P01766 | IGHV3-13 | Immunoglobulin heavy variable 3-13 (Ig heavy chain V-III region BRO) | 0.99 | 3.95E-07 | 0.7 |
| P05546 | SERPIND1 | Heparin cofactor 2 (Heparin cofactor II) (HC-II) (Protease inhibitor leuserpin-2) (HLS2) (Serpin D1) | 0.86 | 4.35E-07 | 0.7 |
| Q14520 | HABP2 | Hyaluronan-binding protein 2 (EC 3.4.21.-) (Factor VII-activating protease) (Factor seven-activating protease) (FSAP) (Hepatocyte growth factor activator-like protein) (Plasma hyaluronan-binding protein) [Cleaved into: Hyaluronan-binding protein 2 50 kDa heavy chain; Hyaluronan-binding protein 2 50 kDa heavy chain alternate form; Hyaluronan-binding protein 2 27 kDa light chain; Hyaluronan-binding protein 2 27 kDa light chain alternate form] | 0.87 | 5.09E-07 | 0.7 |
| P01011 | SERPINA3 | Alpha-1-antichymotrypsin (ACT) (Cell growth-inhibiting gene 24/25 protein) (Serpin A3) [Cleaved into: Alpha-1-antichymotrypsin His-Pro-less] | 0.96 | 5.81E-07 | 0.7 |
| P30086 | PEBP1 | Phosphatidylethanolamine-binding protein 1 (PEBP-1) (HCNPpp) (Neuropolypeptide h3) (Prostatic-binding protein) (Raf kinase inhibitor protein) (RKIP) [Cleaved into: Hippocampal cholinergic neurostimulating peptide (HCNP)] | 0.65 | 6.85E-07 | 0.7 |
| P49908 | SELENOP | Selenoprotein P (SeP) | 0.31 | 7.36E-07 | 0.7 |
| P01008 | SERPINC1 | Antithrombin-III (ATIII) (Serpin C1) | 0.31 | 7.36E-07 | 0.7 |
| P02763 | ORM1 | Alpha-1-acid glycoprotein 1 (AGP 1) (Orosomucoid-1) (OMD 1) | 0.82 | 7.56E-07 | 0.7 |
| P02679 | FGG | Fibrinogen gamma chain | 0.76 | 8.65E-07 | 0.7 |
| P02787 | TF | Serotransferrin (Transferrin) (Beta-1 metal-binding globulin) (Siderophilin) | 0.42 | 1.08E-06 | 0.7 |
| P55058 | PLTP | Phospholipid transfer protein (Lipid transfer protein II) | 0.43 | 1.24E-06 | 0.7 |
| P43251-2 | BTD | Biotinidase (Biotinase) (EC 3.5.1.12) | 0.31 | 1.27E-06 | 0.7 |
| P02675 | FGB | Fibrinogen beta chain [Cleaved into: Fibrinopeptide B; Fibrinogen beta chain] | 0.67 | 1.31E-06 | 0.7 |
| P00740 | F9 | Coagulation factor IX (EC 3.4.21.22) (Christmas factor) (Plasma thromboplastin component) (PTC) [Cleaved into: Coagulation factor IXa light chain; Coagulation factor IXa heavy chain] | 1.07 | 1.82E-06 | 0.7 |
| P01701 | IGLV1-51 | Immunoglobulin lambda variable 1-51 (Ig lambda chain V-I region BL2) (Ig lambda chain V-I region EPS) (Ig lambda chain V-I region NEW) (Ig lambda chain V-I region NIG-64) | 1.21 | 1.95E-06 | 0.7 |
| P61626 | LYZ | Lysozyme C (EC 3.2.1.17) (1,4-beta-N-acetylmuramidase C) | 1.46 | 2.58E-06 | 0.6 |
| P04114 | APOB | Apolipoprotein B-100 (Apo B-100) [Cleaved into: Apolipoprotein B-48 (Apo B-48)] | 2.56 | 2.96E-06 | 0.6 |
| P25311 | AZGP1 | Zinc-alpha-2-glycoprotein (Zn-alpha-2-GP) (Zn-alpha-2-glycoprotein) | 1.20 | 2.96E-06 | 0.6 |
| P01602 | IGKV1-5 | Immunoglobulin kappa variable 1-5 (Ig kappa chain V-I region CAR) (Ig kappa chain V-I region EU) (Ig kappa chain V-I region HK102) (Ig kappa chain V-I region Kue) | 0.71 | 3.08E-06 | 0.6 |
| P07358 | C8B | Complement component C8 beta chain (Complement component 8 subunit beta) | 1.38 | 3.14E-06 | 0.6 |
| P02765 | AHSG | Alpha-2-HS-glycoprotein (Alpha-2-Z-globulin) (Ba-alpha-2-glycoprotein) (Fetuin-A) [Cleaved into: Alpha-2-HS-glycoprotein chain A; Alpha-2-HS-glycoprotein chain B] | 0.55 | 3.25E-06 | 0.6 |
| Q99497 | PARK7 | Protein DJ-1 (DJ-1) (Oncogene DJ1) (Parkinson disease protein 7) (Parkinsonism-associated deglycase) (Protein deglycase DJ-1) (EC 3.1.2.-) (EC 3.5.1.124) | 0.55 | 3.28E-06 | 0.6 |
| P04433 | IGKV3-11 | Immunoglobulin kappa variable 3-11 (Ig kappa chain V-III region VG) | 0.82 | 4.11E-06 | 0.6 |
| O75636 | FCN3 | Ficolin-3 (Collagen/fibrinogen domain-containing lectin 3 p35) (Collagen/fibrinogen domain-containing protein 3) (Hakata antigen) | 3.74 | 4.48E-06 | 0.6 |
| P01871-2 | IGHM | Immunoglobulin heavy constant mu (Ig mu chain C region) (Ig mu chain C region GAL) (Ig mu chain C region OU) | 6.51 | 4.49E-06 | 0.6 |
| P62937 | PPIA | Peptidyl-prolyl cis-trans isomerase A (PPIase A) (EC 5.2.1.8) (Cyclophilin A) (Cyclosporin A-binding protein) (Rotamase A) [Cleaved into: Peptidyl-prolyl cis-trans isomerase A, N-terminally processed] | 0.98 | 5.04E-06 | 0.6 |
| P01023 | A2M | Alpha-2-macroglobulin (Alpha-2-M) (C3 and PZP-like alpha-2-macroglobulin domain-containing protein 5) | 0.72 | 5.09E-06 | 0.6 |
| P02748 | C9 | Complement component C9 [Cleaved into: Complement component C9a; Complement component C9b] | 0.60 | 6.12E-06 | 0.6 |
| P13796 | LCP1 | Plastin-2 (L-plastin) (LC64P) (Lymphocyte cytosolic protein 1) (LCP-1) | 1.67 | 6.12E-06 | 0.6 |
| P07225 | PROS1 | Vitamin K-dependent protein S | 0.35 | 6.12E-06 | 0.6 |
| P01598 | IGKV1-5 | Immunoglobulin kappa variable 1-5 | 0.52 | 6.12E-06 | 0.6 |
| P51884 | LUM | Lumican (Keratan sulfate proteoglycan lumican) (KSPG lumican) | 0.11 | 6.40E-06 | 0.6 |
| P02753 | RBP4 | Retinol-binding protein 4 (Plasma retinol-binding protein) (PRBP) (RBP) [Cleaved into: Plasma retinol-binding protein(1-182); Plasma retinol-binding protein(1-181); Plasma retinol-binding protein(1-179); Plasma retinol-binding protein(1-176)] | 0.84 | 6.40E-06 | 0.6 |
| P02671 | FGA | Fibrinogen alpha chain [Cleaved into: Fibrinopeptide A; Fibrinogen alpha chain] | 0.89 | 6.51E-06 | 0.6 |
| Q06481 | APLP2 | Amyloid-like protein 2 (APLP-2) (APPH) (Amyloid protein homolog) (CDEI box-binding protein) (CDEBP) | 0.69 | 7.64E-06 | 0.6 |
| P19021-5 | PAM | Peptidyl-glycine alpha-amidating monooxygenase (PAM) [Includes: Peptidylglycine alpha-hydroxylating monooxygenase (PHM) (EC 1.14.17.3); Peptidyl-alpha-hydroxyglycine alpha-amidating lyase (EC 4.3.2.5) (Peptidylamidoglycolate lyase) (PAL)] | 0.42 | 1.00E-05 | 0.6 |
| P02750 | LRG1 | Leucine-rich alpha-2-glycoprotein (LRG) | 0.91 | 1.10E-05 | 0.6 |
| O14791-2 | APOL1 | Apolipoprotein L1 (Apolipoprotein L) (Apo-L) (ApoL) (Apolipoprotein L-I) (ApoL-I) | 0.57 | 1.35E-05 | 0.6 |
| P10643 | C7 | Complement component C7 | 0.57 | 1.39E-05 | 0.6 |
| P06331 | IGHV4-34 | Immunoglobulin heavy variable 4-34 (Ig heavy chain V-II region ARH-77) | 0.93 | 1.58E-05 | 0.6 |
| P01876 | IGHA1 | Immunoglobulin heavy constant alpha 1 (Ig alpha-1 chain C region) (Ig alpha-1 chain C region BUR) (Ig alpha-1 chain C region TRO) | 0.94 | 2.25E-05 | 0.6 |
| P06316 | IGLV1-51 | Immunoglobulin lambda variable 1-51 | 0.92 | 2.25E-05 | 0.6 |
| P0C0L5 | C4B; C4B_2 | Complement C4-B (Basic complement C4) (C3 and PZP-like alpha-2-macroglobulin domain-containing protein 3) [Cleaved into: Complement C4 beta chain; Complement C4-B alpha chain; C4a anaphylatoxin; C4b-B; C4d-B; Complement C4 gamma chain] | 0.54 | 2.26E-05 | 0.6 |
| Q06033 | ITIH3 | Inter-alpha-trypsin inhibitor heavy chain H3 (ITI heavy chain H3) (ITI-HC3) (Inter-alpha-inhibitor heavy chain 3) (Serum-derived hyaluronan-associated protein) (SHAP) | 1.86 | 2.26E-05 | 0.6 |
| P09871 | C1S | Complement C1s subcomponent (EC 3.4.21.42) (C1 esterase) (Complement component 1 subcomponent s) [Cleaved into: Complement C1s subcomponent heavy chain; Complement C1s subcomponent light chain] | 0.39 | 3.16E-05 | 0.6 |
| Q9UBR2 | CTSZ | Cathepsin Z (EC 3.4.18.1) (Cathepsin P) (Cathepsin X) | 0.65 | 3.16E-05 | 0.6 |
| P24592 | IGFBP6 | Insulin-like growth factor-binding protein 6 (IBP-6) (IGF-binding protein 6) (IGFBP-6) | 0.57 | 3.90E-05 | 0.5 |
| Q9UBX5 | FBLN5 | Fibulin-5 (FIBL-5) (Developmental arteries and neural crest EGF-like protein) (Dance) (Urine p50 protein) (UP50) | 0.28 | 5.95E-05 | 0.5 |
| P01009 | SERPINA1 | Alpha-1-antitrypsin (Alpha-1 protease inhibitor) (Alpha-1-antiproteinase) (Serpin A1) [Cleaved into: Short peptide from AAT (SPAAT)] | 0.44 | 6.77E-05 | 0.5 |
| P06396 | GSN | Gelsolin (AGEL) (Actin-depolymerizing factor) (ADF) (Brevin) | 0.30 | 7.83E-05 | 0.5 |
| P04211 | IGLV7-43 | Immunoglobulin lambda variable 7-43 (Ig lambda chain V region 4A) | 1.95 | 9.32E-05 | 0.5 |
| P01621 | IGKV3-20 | Immunoglobulin kappa variable 3-20 | 0.54 | 9.48E-05 | 0.5 |
| P01860 | IGHG3 | Immunoglobulin heavy constant gamma 3 (HDC) (Heavy chain disease protein) (Ig gamma-3 chain C region) | 1.00 | 0.000150533 | 0.5 |
| Q6UXB8 | PI16 | Peptidase inhibitor 16 (PI-16) (Cysteine-rich secretory protein 9) (CRISP-9) (PSP94-binding protein) | 0.25 | 0.000167419 | 0.5 |
| P00742 | F10 | Coagulation factor X (EC 3.4.21.6) (Stuart factor) (Stuart-Prower factor) [Cleaved into: Factor X light chain; Factor X heavy chain; Activated factor Xa heavy chain] | 0.58 | 0.000175985 | 0.5 |
| P02042 | HBD | Hemoglobin subunit delta (Delta-globin) (Hemoglobin delta chain) | 1.34 | 0.000235756 | 0.5 |
| P04075 | ALDOA | Fructose-bisphosphate aldolase A (EC 4.1.2.13) (Lung cancer antigen NY-LU-1) (Muscle-type aldolase) | 0.38 | 0.000276068 | 0.5 |
| O75882 | ATRN | Attractin (DPPT-L) (Mahogany homolog) | 0.60 | 0.000330908 | 0.5 |
| P18669 | PGAM1 | Phosphoglycerate mutase 1 (EC 5.4.2.11) (EC 5.4.2.4) (BPG-dependent PGAM 1) (Phosphoglycerate mutase isozyme B) (PGAM-B) | 1.23 | 0.000339535 | 0.5 |
| P36222 | CHI3L1 | Chitinase-3-like protein 1 (39 kDa synovial protein) (Cartilage glycoprotein 39) (CGP-39) (GP-39) (hCGP-39) (YKL-40) | 0.79 | 0.000365911 | 0.5 |
| P01719 | IGLV3-21 | Immunoglobulin lambda variable 3-21 | 1.11 | 0.000623062 | 0.4 |
| P01714 | IGLV3-19 | Immunoglobulin lambda variable 3-19 (Ig lambda chain V-III region SH) | 0.52 | 0.000664705 | 0.4 |
| Q13822-3 | ENPP2 | Ectonucleotide pyrophosphatase/phosphodiesterase family member 2 (E-NPP 2) (EC 3.1.4.39) (Autotaxin) (Extracellular lysophospholipase D) (LysoPLD) | 0.14 | 0.000732628 | 0.4 |
| P08571 | CD14 | Monocyte differentiation antigen CD14 (Myeloid cell-specific leucine-rich glycoprotein) (CD antigen CD14) [Cleaved into: Monocyte differentiation antigen CD14, urinary form; Monocyte differentiation antigen CD14, membrane-bound form] | 1.11 | 0.000819827 | 0.4 |
| P02751 | FN1 | Fibronectin (FN) (Cold-insoluble globulin) (CIG) [Cleaved into: Anastellin; Ugl-Y1; Ugl-Y2; Ugl-Y3] | 0.01 | 0.00092891 | 0.4 |
| P42785-2 | PRCP | Lysosomal Pro-X carboxypeptidase (EC 3.4.16.2) (Angiotensinase C) (Lysosomal carboxypeptidase C) (Proline carboxypeptidase) (Prolylcarboxypeptidase) (PRCP) | -1.33 | 9.92E-06 | 0.6 |
| O75503 | CLN5 | Ceroid-lipofuscinosis neuronal protein 5 (Protein CLN5) [Cleaved into: Ceroid-lipofuscinosis neuronal protein 5, secreted form] | -0.90 | 0.000338734 | 0.5 |
| Q8IV08 | PLD3 | Phospholipase D3 (PLD 3) (EC 3.1.4.4) (Choline phosphatase 3) (HindIII K4L homolog) (Hu-K4) (Phosphatidylcholine-hydrolyzing phospholipase D3) | -2.37 | 0.000696706 | 0.4 |
| P55291 | CDH15 | Cadherin-15 (Cadherin-14) (Muscle cadherin) (M-cadherin) | -0.02 | 0.00083735 | 0.4 |
| P19320 | VCAM1 | Vascular cell adhesion protein 1 (V-CAM 1) (VCAM-1) (INCAM-100) (CD antigen CD106) | 0.80 | 0.001025013 | 0.4 |
| Q9P2S2 | NRXN2 | Neurexin-2 (Neurexin II-alpha) (Neurexin-2-alpha) | -1.59 | 0.001112416 | 0.4 |
| P30043 | BLVRB | Flavin reductase (NADPH) (FR) (EC 1.5.1.30) (Biliverdin reductase B) (BVR-B) (EC 1.3.1.24) (Biliverdin-IX beta-reductase) (Green heme-binding protein) (GHBP) (NADPH-dependent diaphorase) (NADPH-flavin reductase) (FLR) | 3.22 | 0.001112416 | 0.4 |
| P62736 | ACTA2 | Actin, aortic smooth muscle (Alpha-actin-2) (Cell growth-inhibiting gene 46 protein) | 0.87 | 0.001122885 | 0.4 |
| P02144 | MB | Myoglobin | 2.74 | 0.001212736 | 0.4 |
| P98160 | HSPG2 | Basement membrane-specific heparan sulfate proteoglycan core protein (HSPG) (Perlecan) (PLC) [Cleaved into: Endorepellin; LG3 peptide] | 0.04 | 0.001237381 | 0.4 |
| O60462 | NRP2 | Neuropilin-2 (Vascular endothelial cell growth factor 165 receptor 2) | 0.51 | 0.001237381 | 0.4 |
| Q15582 | TGFBI | Transforming growth factor-beta-induced protein ig-h3 (Beta ig-h3) (Kerato-epithelin) (RGD-containing collagen-associated protein) (RGD-CAP) | 0.47 | 0.001440973 | 0.4 |
| P04003 | C4BPA | C4b-binding protein alpha chain (C4bp) (Proline-rich protein) (PRP) | 2.29 | 0.001570867 | 0.4 |
| P60709 | ACTB | Actin, cytoplasmic 1 (Beta-actin) [Cleaved into: Actin, cytoplasmic 1, N-terminally processed] | 1.03 | 0.001615039 | 0.4 |
| Q99983 | OMD | Osteomodulin (Keratan sulfate proteoglycan osteomodulin) (KSPG osteomodulin) (Osteoadherin) (OSAD) | 0.22 | 0.001726598 | 0.4 |
| Q9BTY2 | FUCA2 | Plasma alpha-L-fucosidase (EC 3.2.1.51) (Alpha-L-fucoside fucohydrolase 2) (Alpha-L-fucosidase 2) | 0.32 | 0.002012675 | 0.4 |
| P01708 | IGLV2-11 | Immunoglobulin lambda variable 2-11 | 0.14 | 0.002331944 | 0.4 |
| P02746 | C1QB | Complement C1q subcomponent subunit B | 1.04 | 0.00268895 | 0.4 |
| Q14515 | SPARCL1 | SPARC-like protein 1 (High endothelial venule protein) (Hevin) (MAST 9) | 0.10 | 0.00268895 | 0.4 |
| P08253 | MMP2 | 72 kDa type IV collagenase (EC 3.4.24.24) (72 kDa gelatinase) (Gelatinase A) (Matrix metalloproteinase-2) (MMP-2) (TBE-1) [Cleaved into: PEX] | -0.05 | 0.002806635 | 0.4 |
| P22352 | GPX3 | Glutathione peroxidase 3 (GPx-3) (GSHPx-3) (EC 1.11.1.9) (Extracellular glutathione peroxidase) (Plasma glutathione peroxidase) (GPx-P) (GSHPx-P) | 0.91 | 0.002830035 | 0.4 |
| O14786 | NRP1 | Neuropilin-1 (Vascular endothelial cell growth factor 165 receptor) (CD antigen CD304) | 0.03 | 0.003256055 | 0.4 |
| P06727 | APOA4 | Apolipoprotein A-IV (Apo-AIV) (ApoA-IV) (Apolipoprotein A4) | 0.16 | 0.003256055 | 0.4 |
| P09972 | ALDOC | Fructose-bisphosphate aldolase C (EC 4.1.2.13) (Brain-type aldolase) | 0.18 | 0.003256055 | 0.4 |
| P00736 | C1R | Complement C1r subcomponent (EC 3.4.21.41) (Complement component 1 subcomponent r) [Cleaved into: Complement C1r subcomponent heavy chain; Complement C1r subcomponent light chain] | 0.41 | 0.003256055 | 0.4 |
| P35858-2 | IGFALS | Insulin-like growth factor-binding protein complex acid labile subunit (ALS) | 2.15 | 0.003555055 | 0.3 |
| P01861 | IGHG4 | Immunoglobulin heavy constant gamma 4 (Ig gamma-4 chain C region) | 0.25 | 0.003790341 | 0.3 |
| Q92752 | TNR | Tenascin-R (TN-R) (Janusin) (Restrictin) | 0.05 | 0.004417004 | 0.3 |
| P83593 | IGKV4-1 | Immunoglobulin kappa variable 4-1 | 1.03 | 0.004518921 | 0.3 |
| Q14112 | NID2 | Nidogen-2 (NID-2) (Osteonidogen) | -0.02 | 0.004698038 | 0.3 |
| P17936-2 | IGFBP3 | Insulin-like growth factor-binding protein 3 (IBP-3) (IGF-binding protein 3) (IGFBP-3) | 0.90 | 0.004764092 | 0.3 |
| P48740-2 | MASP1 | Mannan-binding lectin serine protease 1 (EC 3.4.21.-) (Complement factor MASP-3) (Complement-activating component of Ra-reactive factor) (Mannose-binding lectin-associated serine protease 1) (MASP-1) (Mannose-binding protein-associated serine protease) (Ra-reactive factor serine protease p100) (RaRF) (Serine protease 5) [Cleaved into: Mannan-binding lectin serine protease 1 heavy chain; Mannan-binding lectin serine protease 1 light chain] | 0.10 | 0.005008028 | 0.3 |
| P36955 | SERPINF1 | Pigment epithelium-derived factor (PEDF) (Cell proliferation-inducing gene 35 protein) (EPC-1) (Serpin F1) | 0.04 | 0.005255083 | 0.3 |
| Q14956 | GPNMB | Transmembrane glycoprotein NMB (Transmembrane glycoprotein HGFIN) | 2.20 | 0.005395704 | 0.3 |
| P55283 | CDH4 | Cadherin-4 (Retinal cadherin) (R-CAD) (R-cadherin) | 0.32 | 0.00550396 | 0.3 |
| Q15113 | PCOLCE | Procollagen C-endopeptidase enhancer 1 (Procollagen COOH-terminal proteinase enhancer 1) (PCPE-1) (Procollagen C-proteinase enhancer 1) (Type 1 procollagen C-proteinase enhancer protein) (Type I procollagen COOH-terminal proteinase enhancer) | -0.03 | 0.006144236 | 0.3 |
| Q6MZW2 | FSTL4 | Follistatin-related protein 4 (Follistatin-like protein 4) | -2.45 | 0.006197284 | 0.3 |
| P02747 | C1QC | Complement C1q subcomponent subunit C | 1.30 | 0.006370533 | 0.3 |
| P01033 | TIMP1 | Metalloproteinase inhibitor 1 (Erythroid-potentiating activity) (EPA) (Fibroblast collagenase inhibitor) (Collagenase inhibitor) (Tissue inhibitor of metalloproteinases 1) (TIMP-1) | 1.04 | 0.006889352 | 0.3 |
| P0C0L4 | C4A | Complement C4-A (Acidic complement C4) (C3 and PZP-like alpha-2-macroglobulin domain-containing protein 2) [Cleaved into: Complement C4 beta chain; Complement C4-A alpha chain; C4a anaphylatoxin; C4b-A; C4d-A; Complement C4 gamma chain] | 0.38 | 0.007696294 | 0.3 |
| Q86VB7-2 | CD163 | Scavenger receptor cysteine-rich type 1 protein M130 (Hemoglobin scavenger receptor) (CD antigen CD163) [Cleaved into: Soluble CD163 (sCD163)] | 2.45 | 0.007894535 | 0.3 |
| O60241 | ADGRB2 | Adhesion G protein-coupled receptor B2 (Brain-specific angiogenesis inhibitor 2) | -2.05 | 0.009370713 | 0.3 |
| P22897 | MRC1 | Macrophage mannose receptor 1 (MMR) (C-type lectin domain family 13 member D) (C-type lectin domain family 13 member D-like) (Human mannose receptor) (hMR) (Macrophage mannose receptor 1-like protein 1) (CD antigen CD206) | 1.27 | 0.011739791 | 0.3 |
| Q9BZR6 | RTN4R | Reticulon-4 receptor (Nogo receptor) (NgR) (Nogo-66 receptor) | -2.16 | 0.011945604 | 0.3 |
| P00739-2 | HPR | Haptoglobin-related protein | 2.44 | 0.013573547 | 0.3 |
| P04040 | CAT | Catalase (EC 1.11.1.6) | 2.55 | 0.013573547 | 0.3 |
| Q9Y287 | ITM2B | Integral membrane protein 2B (Immature BRI2) (imBRI2) (Protein E25B) (Transmembrane protein BRI) (Bri) [Cleaved into: BRI2, membrane form (Mature BRI2) (mBRI2); BRI2 intracellular domain (BRI2 ICD); BRI2C, soluble form; Bri23 peptide (Bri2-23) (ABri23) (C-terminal peptide) (P23 peptide)] | -1.30 | 0.014213287 | 0.3 |
| Q9H3G5 | CPVL | Probable serine carboxypeptidase CPVL (EC 3.4.16.-) (Carboxypeptidase, vitellogenic-like) (Vitellogenic carboxypeptidase-like protein) (VCP-like protein) (hVLP) | -1.20 | 0.014962854 | 0.3 |
| O94910 | ADGRL1 | Adhesion G protein-coupled receptor L1 (Calcium-independent alpha-latrotoxin receptor 1) (CIRL-1) (Latrophilin-1) (Lectomedin-2) | -1.46 | 0.015023584 | 0.3 |
| P00915 | CA1 | Carbonic anhydrase 1 (EC 4.2.1.1) (Carbonate dehydratase I) (Carbonic anhydrase B) (CAB) (Carbonic anhydrase I) (CA-I) | 2.01 | 0.015092454 | 0.3 |
| P52799 | EFNB2 | Ephrin-B2 (EPH-related receptor tyrosine kinase ligand 5) (LERK-5) (HTK ligand) (HTK-L) | 0.05 | 0.015605128 | 0.3 |
| Q96KN2 | CNDP1 | Beta-Ala-His dipeptidase (EC 3.4.13.20) (CNDP dipeptidase 1) (Carnosine dipeptidase 1) (Glutamate carboxypeptidase-like protein 2) (Serum carnosinase) | 0.63 | 0.016037151 | 0.3 |
| Q08380 | LGALS3BP | Galectin-3-binding protein (Basement membrane autoantigen p105) (Lectin galactoside-binding soluble 3-binding protein) (Mac-2-binding protein) (MAC2BP) (Mac-2 BP) (Tumor-associated antigen 90K) | 0.20 | 0.016160755 | 0.3 |
| O94769 | ECM2 | Extracellular matrix protein 2 (Matrix glycoprotein SC1/ECM2) | 0.23 | 0.016417142 | 0.3 |
| P78509 | RELN | Reelin (EC 3.4.21.-) | -1.38 | 0.016535642 | 0.3 |
| P02461 | COL3A1 | Collagen alpha-1(III) chain | 0.15 | 0.01658048 | 0.3 |
| P63104 | YWHAZ | 14-3-3 protein zeta/delta (Protein kinase C inhibitor protein 1) (KCIP-1) | 1.18 | 0.01658048 | 0.3 |
| P69905 | HBA1; HBA2 | Hemoglobin subunit alpha (Alpha-globin) (Hemoglobin alpha chain) | 2.06 | 0.018080671 | 0.3 |
| P16035 | TIMP2 | Metalloproteinase inhibitor 2 (CSC-21K) (Tissue inhibitor of metalloproteinases 2) (TIMP-2) | 0.15 | 0.018201454 | 0.3 |
| P20774 | OGN | Mimecan (Osteoglycin) (Osteoinductive factor) (OIF) | -0.12 | 0.0200021 | 0.2 |
| P47972 | NPTX2 | Neuronal pentraxin-2 (NP2) (Neuronal pentraxin II) (NP-II) | -1.11 | 0.021400837 | 0.2 |
| Q86UX2 | ITIH5 | Inter-alpha-trypsin inhibitor heavy chain H5 (ITI heavy chain H5) (ITI-HC5) (Inter-alpha-inhibitor heavy chain 5) | -1.07 | 0.021400837 | 0.2 |
| P23470 | PTPRG | Receptor-type tyrosine-protein phosphatase gamma (Protein-tyrosine phosphatase gamma) (R-PTP-gamma) (EC 3.1.3.48) | -1.44 | 0.022120542 | 0.2 |
| Q9Y646 | CPQ | Carboxypeptidase Q (EC 3.4.17.-) (Lysosomal dipeptidase) (Plasma glutamate carboxypeptidase) | -1.19 | 0.02309587 | 0.2 |
| Q96B86-4 | RGMA | Repulsive guidance molecule A (RGM domain family member A) | -1.36 | 0.023153305 | 0.2 |
| Q12841 | FSTL1 | Follistatin-related protein 1 (Follistatin-like protein 1) | 0.47 | 0.023153305 | 0.2 |
| P13473-3 | LAMP2 | Lysosome-associated membrane glycoprotein 2 (LAMP-2) (Lysosome-associated membrane protein 2) (CD107 antigen-like family member B) (LGP-96) (CD antigen CD107b) | -0.70 | 0.024013392 | 0.2 |
| Q13421-2 | MSLN | Mesothelin (CAK1 antigen) (Pre-pro-megakaryocyte-potentiating factor) [Cleaved into: Megakaryocyte-potentiating factor (MPF); Mesothelin, cleaved form] | 0.95 | 0.025644932 | 0.3 |
| P04278 | SHBG | Sex hormone-binding globulin (SHBG) (Sex steroid-binding protein) (SBP) (Testis-specific androgen-binding protein) (ABP) (Testosterone-estradiol-binding globulin) (TeBG) (Testosterone-estrogen-binding globulin) | 1.85 | 0.027096964 | 0.2 |
| P13611 | VCAN | Versican core protein (Chondroitin sulfate proteoglycan core protein 2) (Chondroitin sulfate proteoglycan 2) (Glial hyaluronate-binding protein) (GHAP) (Large fibroblast proteoglycan) (PG-M) | -0.02 | 0.02858787 | 0.2 |
| P22304 | IDS | Iduronate 2-sulfatase (EC 3.1.6.13) (Alpha-L-iduronate sulfate sulfatase) (Idursulfase) [Cleaved into: Iduronate 2-sulfatase 42 kDa chain; Iduronate 2-sulfatase 14 kDa chain] | -1.75 | 0.029191638 | 0.2 |
| Q9ULB1-3 | NRXN1 | Neurexin-1 (Neurexin I-alpha) (Neurexin-1-alpha) | 0.07 | 0.029522432 | 0.2 |
| Q9UHL4 | DPP7 | Dipeptidyl peptidase 2 (EC 3.4.14.2) (Dipeptidyl aminopeptidase II) (Dipeptidyl peptidase 7) (Dipeptidyl peptidase II) (DPP II) (Quiescent cell proline dipeptidase) | -1.64 | 0.030799122 | 0.2 |
| O14498 | ISLR | Immunoglobulin superfamily containing leucine-rich repeat protein | 0.07 | 0.031411556 | 0.2 |
| Q8TEU8 | WFIKKN2 | WAP, Kazal, immunoglobulin, Kunitz and NTR domain-containing protein 2 (Growth and differentiation factor-associated serum protein 1) (GASP-1) (hGASP-1) (WAP, follistatin, immunoglobulin, Kunitz and NTR domain-containing-related protein) (WFIKKN-related protein) | 0.59 | 0.031411556 | 0.2 |
| P12111 | COL6A3 | Collagen alpha-3(VI) chain | 0.39 | 0.031565236 | 0.2 |
| P68871 | HBB | Hemoglobin subunit beta (Beta-globin) (Hemoglobin beta chain) [Cleaved into: LVV-hemorphin-7; Spinorphin] | 2.09 | 0.035002055 | 0.2 |
| Q9Y5I4 | PCDHAC2 | Protocadherin alpha-C2 (PCDH-alpha-C2) | -1.95 | 0.036161089 | 0.2 |
| P27797 | CALR | Calreticulin (CRP55) (Calregulin) (Endoplasmic reticulum resident protein 60) (ERp60) (HACBP) (grp60) | -0.08 | 0.036161089 | 0.2 |
| Q6NW40 | RGMB | RGM domain family member B (DRG11-responsive axonal guidance and outgrowth of neurite) (DRAGON) | -1.73 | 0.036161089 | 0.2 |
| O14773 | TPP1 | Tripeptidyl-peptidase 1 (TPP-1) (EC 3.4.14.9) (Cell growth-inhibiting gene 1 protein) (Lysosomal pepstatin-insensitive protease) (LPIC) (Tripeptidyl aminopeptidase) (Tripeptidyl-peptidase I) (TPP-I) | -0.39 | 0.036386864 | 0.2 |
| P02745 | C1QA | Complement C1q subcomponent subunit A | 0.73 | 0.036575472 | 0.2 |
| P23142 | FBLN1 | Fibulin-1 (FIBL-1) | 0.24 | 0.037762451 | 0.2 |
| P01019 | AGT | Angiotensinogen (Serpin A8) [Cleaved into: Angiotensin-1 (Angiotensin 1-10) (Angiotensin I) (Ang I); Angiotensin-2 (Angiotensin 1-8) (Angiotensin II) (Ang II); Angiotensin-3 (Angiotensin 2-8) (Angiotensin III) (Ang III) (Des-Asp[1]-angiotensin II); Angiotensin-4 (Angiotensin 3-8) (Angiotensin IV) (Ang IV); Angiotensin 1-9; Angiotensin 1-7; Angiotensin 1-5; Angiotensin 1-4] | 0.09 | 0.038266328 | 0.2 |
| O14917 | PCDH17 | Protocadherin-17 (Protocadherin-68) | -2.54 | 0.039536909 | 0.2 |
| Q9Y279 | VSIG4 | V-set and immunoglobulin domain-containing protein 4 (Protein Z39Ig) | 2.54 | 0.040295281 | 0.2 |
| A6NJ16 | IGHV4OR15-8 | Putative V-set and immunoglobulin domain-containing-like protein IGHV4OR15-8 | 0.79 | 0.042163284 | 0.2 |
| P04066 | FUCA1 | Tissue alpha-L-fucosidase (EC 3.2.1.51) (Alpha-L-fucosidase I) (Alpha-L-fucoside fucohydrolase 1) (Alpha-L-fucosidase 1) | -0.59 | 0.042709412 | 0.2 |
| Q53EL9 | SEZ6 | Seizure protein 6 homolog (SEZ-6) (hSEZ-6) | -1.08 | 0.042709412 | 0.2 |
| Q96FE5 | LINGO1 | Leucine-rich repeat and immunoglobulin-like domain-containing nogo receptor-interacting protein 1 (Leucine-rich repeat and immunoglobulin domain-containing protein 1) (Leucine-rich repeat neuronal protein 1) (Leucine-rich repeat neuronal protein 6A) | -3.39 | 0.043626452 | 0.2 |
| P07686 | HEXB | Beta-hexosaminidase subunit beta (EC 3.2.1.52) (Beta-N-acetylhexosaminidase subunit beta) (Hexosaminidase subunit B) (Cervical cancer proto-oncogene 7 protein) (HCC-7) (N-acetyl-beta-glucosaminidase subunit beta) [Cleaved into: Beta-hexosaminidase subunit beta chain B; Beta-hexosaminidase subunit beta chain A] | -1.14 | 0.044026323 | 0.2 |
| Q07954 | LRP1 | Prolow-density lipoprotein receptor-related protein 1 (LRP-1) (Alpha-2-macroglobulin receptor) (A2MR) (Apolipoprotein E receptor) (APOER) (CD antigen CD91) [Cleaved into: Low-density lipoprotein receptor-related protein 1 85 kDa subunit (LRP-85); Low-density lipoprotein receptor-related protein 1 515 kDa subunit (LRP-515); Low-density lipoprotein receptor-related protein 1 intracellular domain (LRPICD)] | -0.83 | 0.044917367 | 0.2 |
| P00738 | HP | Haptoglobin (Zonulin) [Cleaved into: Haptoglobin alpha chain; Haptoglobin beta chain] | 1.32 | 0.046451796 | 0.2 |
| Q9H2E6-2 | SEMA6A | Semaphorin-6A (Semaphorin VIA) (Sema VIA) (Semaphorin-6A-1) (SEMA6A-1) | -1.65 | 0.046883513 | 0.2 |
| P10909-2 | CLU | Clusterin (Aging-associated gene 4 protein) (Apolipoprotein J) (Apo-J) (Complement cytolysis inhibitor) (CLI) (Complement-associated protein SP-40,40) (Ku70-binding protein 1) (NA1/NA2) (Testosterone-repressed prostate message 2) (TRPM-2) [Cleaved into: Clusterin beta chain (ApoJalpha) (Complement cytolysis inhibitor a chain); Clusterin alpha chain (ApoJbeta) (Complement cytolysis inhibitor b chain)] | -0.10 | 0.048035717 | 0.2 |
| P07333 | CSF1R | Macrophage colony-stimulating factor 1 receptor (CSF-1 receptor) (CSF-1-R) (CSF-1R) (M-CSF-R) (EC 2.7.10.1) (Proto-oncogene c-Fms) (CD antigen CD115) | 0.35 | 0.04895802 | 0.2 |
| P22748 | CA4 | Carbonic anhydrase 4 (EC 4.2.1.1) (Carbonate dehydratase IV) (Carbonic anhydrase IV) (CA-IV) | -1.42 | 0.051175066 | 0.2 |
| Q9HAT2 | SIAE | Sialate O-acetylesterase (EC 3.1.1.53) (H-Lse) (Sialic acid-specific 9-O-acetylesterase) | -1.87 | 0.059681999 | 0.2 |
| Q9Y6R7 | FCGBP | IgGFc-binding protein (Fcgamma-binding protein antigen) (FcgammaBP) | 2.17 | 0.059877606 | 0.2 |
| Q9UBQ6 | EXTL2 | Exostosin-like 2 (EC 2.4.1.223) (Alpha-1,4-N-acetylhexosaminyltransferase EXTL2) (Alpha-GalNAcT EXTL2) (EXT-related protein 2) (Glucuronyl-galactosyl-proteoglycan 4-alpha-N-acetylglucosaminyltransferase) [Cleaved into: Processed exostosin-like 2] | -0.88 | 0.060748446 | 0.2 |
| Q9Y6N7-2 | ROBO1 | Roundabout homolog 1 (Deleted in U twenty twenty) (H-Robo-1) | -0.83 | 0.060748446 | 0.2 |
| P08123 | COL1A2 | Collagen alpha-2(I) chain (Alpha-2 type I collagen) | 0.51 | 0.063821144 | 0.2 |
| P51888 | PRELP | Prolargin (Proline-arginine-rich end leucine-rich repeat protein) | -0.21 | 0.066844961 | 0.2 |
| P61769 | B2M | Beta-2-microglobulin [Cleaved into: Beta-2-microglobulin form pI 5.3] | 0.80 | 0.06800041 | 0.2 |
| P23284 | PPIB | Peptidyl-prolyl cis-trans isomerase B (PPIase B) (EC 5.2.1.8) (CYP-S1) (Cyclophilin B) (Rotamase B) (S-cyclophilin) (SCYLP) | -1.50 | 0.068969509 | 0.2 |
| Q14019 | COTL1 | Coactosin-like protein | 0.70 | 0.069262677 | 0.2 |
| Q96NZ9-3 | PRAP1 | Proline-rich acidic protein 1 (Epididymis tissue protein Li 178) (Uterine-specific proline-rich acidic protein) | -0.89 | 0.074863694 | 0.2 |
| P07195 | LDHB | L-lactate dehydrogenase B chain (LDH-B) (EC 1.1.1.27) (LDH heart subunit) (LDH-H) (Renal carcinoma antigen NY-REN-46) | 0.06 | 0.078126963 | 0.2 |
| P35542 | SAA4 | Serum amyloid A-4 protein (Constitutively expressed serum amyloid A protein) (C-SAA) | 0.25 | 0.079674293 | 0.2 |
| P02649 | APOE | Apolipoprotein E (Apo-E) | 0.07 | 0.081961594 | 0.2 |
| P48058 | GRIA4 | Glutamate receptor 4 (GluR-4) (GluR4) (AMPA-selective glutamate receptor 4) (GluR-D) (Glutamate receptor ionotropic, AMPA 4) (GluA4) | 0.15 | 0.082460557 | 0.2 |
| Q969P0 | IGSF8 | Immunoglobulin superfamily member 8 (IgSF8) (CD81 partner 3) (Glu-Trp-Ile EWI motif-containing protein 2) (EWI-2) (Keratinocytes-associated transmembrane protein 4) (KCT-4) (LIR-D1) (Prostaglandin regulatory-like protein) (PGRL) (CD antigen CD316) | -0.52 | 0.085056259 | 0.1 |
| P14314 | PRKCSH | Glucosidase 2 subunit beta (80K-H protein) (Glucosidase II subunit beta) (Protein kinase C substrate 60.1 kDa protein heavy chain) (PKCSH) | -0.18 | 0.094430002 | 0.1 |
| Q6UX71 | PLXDC2 | Plexin domain-containing protein 2 (Tumor endothelial marker 7-related protein) | 0.15 | 0.094783605 | 0.1 |
| P04080 | CSTB | Cystatin-B (CPI-B) (Liver thiol proteinase inhibitor) (Stefin-B) | 1.11 | 0.095786559 | 0.1 |
| P18428 | LBP | Lipopolysaccharide-binding protein (LBP) | 1.58 | 0.103161565 | 0.1 |
| Q9C0A0 | CNTNAP4 | Contactin-associated protein-like 4 (Cell recognition molecule Caspr4) | -0.32 | 0.106587764 | 0.1 |
| Q92823-5 | NRCAM | Neuronal cell adhesion molecule (Nr-CAM) (Neuronal surface protein Bravo) (hBravo) (NgCAM-related cell adhesion molecule) (Ng-CAM-related) | -0.99 | 0.107911611 | 0.1 |
| O15335 | CHAD | Chondroadherin (Cartilage leucine-rich protein) | -2.00 | 0.115670858 | 0.1 |
| P04156 | PRNP | Major prion protein (PrP) (ASCR) (PrP27-30) (PrP33-35C) (CD antigen CD230) | -0.12 | 0.11918288 | 0.1 |
| P09486 | SPARC | SPARC (Basement-membrane protein 40) (BM-40) (Osteonectin) (ON) (Secreted protein acidic and rich in cysteine) | 0.10 | 0.120640337 | 0.1 |
| P04406 | GAPDH | Glyceraldehyde-3-phosphate dehydrogenase (GAPDH) (EC 1.2.1.12) (Peptidyl-cysteine S-nitrosylase GAPDH) (EC 2.6.99.-) | 0.46 | 0.120640337 | 0.1 |
| P07858 | CTSB | Cathepsin B (EC 3.4.22.1) (APP secretase) (APPS) (Cathepsin B1) [Cleaved into: Cathepsin B light chain; Cathepsin B heavy chain] | 0.82 | 0.123120934 | 0.1 |
| Q06828 | FMOD | Fibromodulin (FM) (Collagen-binding 59 kDa protein) (Keratan sulfate proteoglycan fibromodulin) (KSPG fibromodulin) | -0.05 | 0.127453112 | 0.1 |
| Q99674-5 | CGREF1 | Cell growth regulator with EF hand domain protein 1 (Cell growth regulatory gene 11 protein) (Hydrophobestin) | -1.71 | 0.130654203 | 0.1 |
| P49641 | MAN2A2 | Alpha-mannosidase 2x (EC 3.2.1.114) (Alpha-mannosidase IIx) (Man IIx) (Mannosidase alpha class 2A member 2) (Mannosyl-oligosaccharide 1,3-1,6-alpha-mannosidase) | -0.31 | 0.137176022 | 0.1 |
| P17677-2 | GAP43 | Neuromodulin (Axonal membrane protein GAP-43) (Growth-associated protein 43) (Neural phosphoprotein B-50) (pp46) | -0.08 | 0.137742808 | 0.1 |
| Q9UPU3 | SORCS3 | VPS10 domain-containing receptor SorCS3 | -1.35 | 0.138101831 | 0.1 |
| Q92954 | PRG4 | Proteoglycan 4 (Lubricin) (Megakaryocyte-stimulating factor) (Superficial zone proteoglycan) [Cleaved into: Proteoglycan 4 C-terminal part] | 0.56 | 0.138315157 | 0.1 |
| O00461 | GOLIM4 | Golgi integral membrane protein 4 (Golgi integral membrane protein, cis) (GIMPc) (Golgi phosphoprotein 4) (Golgi-localized phosphoprotein of 130 kDa) (Golgi phosphoprotein of 130 kDa) | -0.15 | 0.14068864 | 0.1 |
| O00468 | AGRN | Agrin [Cleaved into: Agrin N-terminal 110 kDa subunit; Agrin C-terminal 110 kDa subunit; Agrin C-terminal 90 kDa fragment (C90); Agrin C-terminal 22 kDa fragment (C22)] | -1.22 | 0.14068864 | 0.1 |
| Q9ULF5 | SLC39A10 | Zinc transporter ZIP10 (Solute carrier family 39 member 10) (Zrt- and Irt-like protein 10) (ZIP-10) | -1.40 | 0.140877977 | 0.1 |
| P14543 | NID1 | Nidogen-1 (NID-1) (Entactin) | 0.05 | 0.143875661 | 0.1 |
| O75787 | ATP6AP2 | Renin receptor (ATPase H(+)-transporting lysosomal accessory protein 2) (ATPase H(+)-transporting lysosomal-interacting protein 2) (ER-localized type I transmembrane adaptor) (Embryonic liver differentiation factor 10) (N14F) (Renin/prorenin receptor) (Vacuolar ATP synthase membrane sector-associated protein M8-9) (ATP6M8-9) (V-ATPase M8.9 subunit) | -1.34 | 0.145022154 | 0.1 |
| P10599 | TXN | Thioredoxin (Trx) (ATL-derived factor) (ADF) (Surface-associated sulphydryl protein) (SASP) | -0.15 | 0.149429328 | 0.1 |
| P09417 | QDPR | Dihydropteridine reductase (EC 1.5.1.34) (HDHPR) (Quinoid dihydropteridine reductase) (Short chain dehydrogenase/reductase family 33C member 1) | -1.90 | 0.149884036 | 0.1 |
| P0DJI8 | SAA1 | Serum amyloid A-1 protein (SAA) [Cleaved into: Amyloid protein A (Amyloid fibril protein AA); Serum amyloid protein A(2-104); Serum amyloid protein A(3-104); Serum amyloid protein A(2-103); Serum amyloid protein A(2-102); Serum amyloid protein A(4-101)] | 1.94 | 0.150179695 | 0.1 |
| Q13554 | CAMK2B | Calcium/calmodulin-dependent protein kinase type II subunit beta (CaM kinase II subunit beta) (CaMK-II subunit beta) (EC 2.7.11.17) | -1.09 | 0.150179695 | 0.1 |
| P01210 | PENK | Proenkephalin-A [Cleaved into: Synenkephalin; Met-enkephalin (Opioid growth factor) (OGF); PENK(114-133); PENK(143-183); Met-enkephalin-Arg-Gly-Leu; Leu-enkephalin; PENK(237-258); Met-enkephalin-Arg-Phe] | -0.90 | 0.15780729 | 0.1 |
| P16870 | CPE | Carboxypeptidase E (CPE) (EC 3.4.17.10) (Carboxypeptidase H) (CPH) (Enkephalin convertase) (Prohormone-processing carboxypeptidase) | -0.26 | 0.15780729 | 0.1 |
| P54289 | CACNA2D1 | Voltage-dependent calcium channel subunit alpha-2/delta-1 (Voltage-gated calcium channel subunit alpha-2/delta-1) [Cleaved into: Voltage-dependent calcium channel subunit alpha-2-1; Voltage-dependent calcium channel subunit delta-1] | -0.93 | 0.168798828 | 0.1 |
| Q86UN3 | RTN4RL2 | Reticulon-4 receptor-like 2 (Nogo receptor-like 3) (Nogo-66 receptor homolog 1) (Nogo-66 receptor-related protein 2) (NgR2) | -1.62 | 0.169258793 | 0.1 |
| P54764 | EPHA4 | Ephrin type-A receptor 4 (EC 2.7.10.1) (EPH-like kinase 8) (EK8) (hEK8) (Tyrosine-protein kinase TYRO1) (Tyrosine-protein kinase receptor SEK) | -1.21 | 0.174187225 | 0.1 |
| P39060 | COL18A1 | Collagen alpha-1(XVIII) chain [Cleaved into: Endostatin] | -0.18 | 0.174187225 | 0.1 |
| Q9NYQ8 | FAT2 | Protocadherin Fat 2 (hFat2) (Cadherin family member 8) (Multiple epidermal growth factor-like domains protein 1) (Multiple EGF-like domains protein 1) | -1.11 | 0.174187225 | 0.1 |
| Q15818 | NPTX1 | Neuronal pentraxin-1 (NP1) (Neuronal pentraxin I) (NP-I) | -0.95 | 0.18834457 | 0.1 |
| P01911 | HLA-DRB1 | HLA class II histocompatibility antigen, DRB1-15 beta chain (DW2.2/DR2.2) (MHC class II antigen DRB1*15) | 1.02 | 0.194310281 | 0.1 |
| P08670 | VIM | Vimentin | 0.88 | 0.203387959 | 0.1 |
| O43556-4 | SGCE | Epsilon-sarcoglycan (Epsilon-SG) | -0.93 | 0.208780554 | 0.1 |
| Q16610-4 | ECM1 | Extracellular matrix protein 1 (Secretory component p85) | -0.51 | 0.208780554 | 0.1 |
| Q02246 | CNTN2 | Contactin-2 (Axonal glycoprotein TAG-1) (Axonin-1) (Transient axonal glycoprotein 1) (TAX-1) | -0.57 | 0.219299345 | 0.1 |
| O00391 | QSOX1 | Sulfhydryl oxidase 1 (hQSOX) (EC 1.8.3.2) (Quiescin Q6) | -0.24 | 0.226454665 | 0.1 |
| Q92859 | NEO1 | Neogenin (Immunoglobulin superfamily DCC subclass member 2) | -1.07 | 0.226454665 | 0.1 |
| Q9UBP4 | DKK3 | Dickkopf-related protein 3 (Dickkopf-3) (Dkk-3) (hDkk-3) | -0.20 | 0.226953003 | 0.1 |
| Q16849 | PTPRN | Receptor-type tyrosine-protein phosphatase-like N (R-PTP-N) (Islet cell antigen 512) (ICA 512) (Islet cell autoantigen 3) (PTP IA-2) [Cleaved into: ICA512-N-terminal fragment (ICA512-NTF); ICA512-transmembrane fragment (ICA512-TMF); ICA512-cleaved cytosolic fragment (ICA512-CCF)] | -1.05 | 0.226953003 | 0.1 |
| P55083-2 | MFAP4 | Microfibril-associated glycoprotein 4 | -0.66 | 0.238158231 | 0.1 |
| O43505 | B4GAT1 | Beta-1,4-glucuronyltransferase 1 (EC 2.4.1.-) (I-beta-1,3-N-acetylglucosaminyltransferase) (iGnT) (N-acetyllactosaminide beta-1,3-N-acetylglucosaminyltransferase) (Poly-N-acetyllactosamine extension enzyme) (UDP-GlcNAc:betaGal beta-1,3-N-acetylglucosaminyltransferase 1) | -0.83 | 0.252457094 | 0.1 |
| Q7Z7M0 | MEGF8 | Multiple epidermal growth factor-like domains protein 8 (Multiple EGF-like domains protein 8) (Epidermal growth factor-like protein 4) (EGF-like protein 4) | -0.21 | 0.255955363 | 0.1 |
| Q6UX73 | C16orf89 | UPF0764 protein C16orf89 | -0.38 | 0.255955363 | 0.1 |
| Q8WVQ1 | CANT1 | Soluble calcium-activated nucleotidase 1 (SCAN-1) (EC 3.6.1.6) (Apyrase homolog) (Putative MAPK-activating protein PM09) (Putative NF-kappa-B-activating protein 107) | -0.65 | 0.26098961 | 0.1 |
| Q16769 | QPCT | Glutaminyl-peptide cyclotransferase (EC 2.3.2.5) (Glutaminyl cyclase) (QC) (sQC) (Glutaminyl-tRNA cyclotransferase) (Glutamyl cyclase) (EC) | -1.12 | 0.261888265 | 0.1 |
| P61916 | NPC2 | Epididymal secretory protein E1 (Human epididymis-specific protein 1) (He1) (Niemann-Pick disease type C2 protein) | -0.22 | 0.271253401 | 0.1 |
| P10451-5 | SPP1 | Osteopontin (Bone sialoprotein 1) (Nephropontin) (Secreted phosphoprotein 1) (SPP-1) (Urinary stone protein) (Uropontin) | 0.30 | 0.281295981 | 0.1 |
| Q96S96 | PEBP4 | Phosphatidylethanolamine-binding protein 4 (PEBP-4) (hPEBP4) (Protein cousin-of-RKIP 1) | 0.00 | 0.28345072 | 0.1 |
| O75144-2 | ICOSLG | ICOS ligand (B7 homolog 2) (B7-H2) (B7-like protein Gl50) (B7-related protein 1) (B7RP-1) (CD antigen CD275) | -0.49 | 0.29608132 | 0.1 |
| P40925-3 | MDH1 | Malate dehydrogenase, cytoplasmic (EC 1.1.1.37) (Cytosolic malate dehydrogenase) (Diiodophenylpyruvate reductase) (EC 1.1.1.96) | -1.80 | 0.29608132 | 0.1 |
| P10586 | PTPRF | Receptor-type tyrosine-protein phosphatase F (EC 3.1.3.48) (Leukocyte common antigen related) (LAR) | -2.95 | 0.296334869 | 0.1 |
| O95502 | NPTXR | Neuronal pentraxin receptor | -0.92 | 0.30439259 | 0.1 |
| P78324-2 | SIRPA | Tyrosine-protein phosphatase non-receptor type substrate 1 (SHP substrate 1) (SHPS-1) (Brain Ig-like molecule with tyrosine-based activation motifs) (Bit) (CD172 antigen-like family member A) (Inhibitory receptor SHPS-1) (Macrophage fusion receptor) (MyD-1 antigen) (Signal-regulatory protein alpha-1) (Sirp-alpha-1) (Signal-regulatory protein alpha-2) (Sirp-alpha-2) (Signal-regulatory protein alpha-3) (Sirp-alpha-3) (p84) (CD antigen CD172a) | -0.72 | 0.30439259 | 0.1 |
| Q14118 | DAG1 | Dystroglycan (Dystrophin-associated glycoprotein 1) [Cleaved into: Alpha-dystroglycan (Alpha-DG); Beta-dystroglycan (Beta-DG)] | -0.31 | 0.312148186 | 0.1 |
| P06733 | ENO1 | Alpha-enolase (EC 4.2.1.11) (2-phospho-D-glycerate hydro-lyase) (C-myc promoter-binding protein) (Enolase 1) (MBP-1) (MPB-1) (Non-neural enolase) (NNE) (Phosphopyruvate hydratase) (Plasminogen-binding protein) | -0.31 | 0.312148186 | 0.1 |
| P05060 | CHGB | Secretogranin-1 (Chromogranin-B) (CgB) (Secretogranin I) (SgI) [Cleaved into: PE-11; GAWK peptide; CCB peptide] | -0.34 | 0.316471885 | 0.1 |
| Q02818 | NUCB1 | Nucleobindin-1 (CALNUC) | -0.07 | 0.321167861 | 0.1 |
| Q96GW7 | BCAN | Brevican core protein (Brain-enriched hyaluronan-binding protein) (BEHAB) (Chondroitin sulfate proteoglycan 7) | -1.10 | 0.324156741 | 0.1 |
| Q9HAR2-4 | ADGRL3 | Adhesion G protein-coupled receptor L3 (Calcium-independent alpha-latrotoxin receptor 3) (CIRL-3) (Latrophilin-3) (Lectomedin-3) | -0.72 | 0.324156741 | 0.1 |
| Q5FWE3 | PRRT3 | Proline-rich transmembrane protein 3 | -0.94 | 0.33956297 | 0.1 |
| Q9NYX4 | CALY | Neuron-specific vesicular protein calcyon | -1.24 | 0.347813928 | 0.1 |
| Q9Y4C0-4 | NRXN3 | Neurexin-3 (Neurexin III-alpha) (Neurexin-3-alpha) | -0.91 | 0.364444692 | 0.1 |
| Q15782 | CHI3L2 | Chitinase-3-like protein 2 (Chondrocyte protein 39) (YKL-39) | 0.32 | 0.372406444 | 0.1 |
| Q8NBJ4 | GOLM1 | Golgi membrane protein 1 (Golgi membrane protein GP73) (Golgi phosphoprotein 2) | -0.08 | 0.381571594 | 0.0 |
| P00558 | PGK1 | Phosphoglycerate kinase 1 (EC 2.7.2.3) (Cell migration-inducing gene 10 protein) (Primer recognition protein 2) (PRP 2) | 0.40 | 0.381571594 | 0.0 |
| Q6ZMI3 | GLDN | Gliomedin [Cleaved into: Gliomedin shedded ectodomain] | -0.50 | 0.393820802 | 0.0 |
| Q15904 | ATP6AP1 | V-type proton ATPase subunit S1 (V-ATPase subunit S1) (Protein XAP-3) (V-ATPase Ac45 subunit) (V-ATPase S1 accessory protein) (Vacuolar proton pump subunit S1) | -0.67 | 0.397614639 | 0.0 |
| O00584 | RNASET2 | Ribonuclease T2 (EC 3.1.27.-) (Ribonuclease 6) | 0.12 | 0.403043509 | 0.0 |
| P22692 | IGFBP4 | Insulin-like growth factor-binding protein 4 (IBP-4) (IGF-binding protein 4) (IGFBP-4) | 0.06 | 0.405649232 | 0.0 |
| P23142-4 | FBLN1 | Fibulin-1 (FIBL-1) | 0.11 | 0.407836704 | 0.0 |
| P30479 | HLA-B | HLA class I histocompatibility antigen, B-41 alpha chain (Bw-41) (MHC class I antigen B*41) | 2.60 | 0.408702718 | 0.0 |
| O00533-2 | CHL1 | Neural cell adhesion molecule L1-like protein (Close homolog of L1) [Cleaved into: Processed neural cell adhesion molecule L1-like protein] | -0.30 | 0.408702718 | 0.0 |
| Q9NZK5 | CECR1 | Adenosine deaminase CECR1 (EC 3.5.4.4) (Cat eye syndrome critical region protein 1) | 2.01 | 0.408702718 | 0.0 |
| Q9Y4L1 | HYOU1 | Hypoxia up-regulated protein 1 (150 kDa oxygen-regulated protein) (ORP-150) (170 kDa glucose-regulated protein) (GRP-170) | -0.74 | 0.424624331 | 0.0 |
| Q13449 | LSAMP | Limbic system-associated membrane protein (LSAMP) (IgLON family member 3) | -0.43 | 0.434417488 | 0.0 |
| Q9HCB6 | SPON1 | Spondin-1 (F-spondin) (Vascular smooth muscle cell growth-promoting factor) | -1.10 | 0.447602139 | 0.0 |
| P04180 | LCAT | Phosphatidylcholine-sterol acyltransferase (EC 2.3.1.43) (Lecithin-cholesterol acyltransferase) (Phospholipid-cholesterol acyltransferase) | -0.76 | 0.447602139 | 0.0 |
| P09603 | CSF1 | Macrophage colony-stimulating factor 1 (CSF-1) (M-CSF) (MCSF) (Lanimostim) [Cleaved into: Processed macrophage colony-stimulating factor 1] | -0.14 | 0.448075754 | 0.0 |
| Q9NQ79 | CRTAC1 | Cartilage acidic protein 1 (68 kDa chondrocyte-expressed protein) (CEP-68) (ASPIC) | -0.52 | 0.448145429 | 0.0 |
| P07585 | DCN | Decorin (Bone proteoglycan II) (PG-S2) (PG40) | -0.83 | 0.448145429 | 0.0 |
| Q8N3J6-3 | CADM2 | Cell adhesion molecule 2 (Immunoglobulin superfamily member 4D) (IgSF4D) (Nectin-like protein 3) (NECL-3) (Synaptic cell adhesion molecule 2) (SynCAM 2) | -0.29 | 0.448742057 | 0.0 |
| P41222 | PTGDS | Prostaglandin-H2 D-isomerase (EC 5.3.99.2) (Beta-trace protein) (Cerebrin-28) (Glutathione-independent PGD synthase) (Lipocalin-type prostaglandin-D synthase) (Prostaglandin-D2 synthase) (PGD2 synthase) (PGDS) (PGDS2) | -0.25 | 0.451405707 | 0.0 |
| Q14393 | GAS6 | Growth arrest-specific protein 6 (GAS-6) (AXL receptor tyrosine kinase ligand) | -0.06 | 0.458468551 | 0.0 |
| P15151 | PVR | Poliovirus receptor (Nectin-like protein 5) (NECL-5) (CD antigen CD155) | -0.47 | 0.467103541 | 0.0 |
| Q9UJA9 | ENPP5 | Ectonucleotide pyrophosphatase/phosphodiesterase family member 5 (E-NPP 5) (NPP-5) (EC 3.1.-.-) | -0.70 | 0.475187754 | 0.0 |
| Q92743 | HTRA1 | Serine protease HTRA1 (EC 3.4.21.-) (High-temperature requirement A serine peptidase 1) (L56) (Serine protease 11) | -1.06 | 0.489055322 | 0.0 |
| P40189 | IL6ST | Interleukin-6 receptor subunit beta (IL-6 receptor subunit beta) (IL-6R subunit beta) (IL-6R-beta) (IL-6RB) (CDw130) (Interleukin-6 signal transducer) (Membrane glycoprotein 130) (gp130) (Oncostatin-M receptor subunit alpha) (CD antigen CD130) | -0.78 | 0.49358962 | 0.0 |
| Q8TAG5-2 | VSTM2A | V-set and transmembrane domain-containing protein 2A | -1.07 | 0.499648403 | 0.0 |
| Q8WWX9 | SELENOM | Selenoprotein M (SelM) | -1.66 | 0.499648403 | 0.0 |
| Q08629 | SPOCK1 | Testican-1 (Protein SPOCK) | -0.69 | 0.499648403 | 0.0 |
| Q9BY67-3 | CADM1 | Cell adhesion molecule 1 (Immunoglobulin superfamily member 4) (IgSF4) (Nectin-like protein 2) (NECL-2) (Spermatogenic immunoglobulin superfamily) (SgIgSF) (Synaptic cell adhesion molecule) (SynCAM) (Tumor suppressor in lung cancer 1) (TSLC-1) | -0.65 | 0.507072058 | 0.0 |
| P55285 | CDH6 | Cadherin-6 (Kidney cadherin) (K-cadherin) | -1.02 | 0.507404146 | 0.0 |
| P05452 | CLEC3B | Tetranectin (TN) (C-type lectin domain family 3 member B) (Plasminogen kringle 4-binding protein) | -0.41 | 0.519168901 | 0.0 |
| P05067 | APP | Amyloid beta A4 protein (ABPP) (APPI) (APP) (Alzheimer disease amyloid protein) (Amyloid precursor protein) (Beta-amyloid precursor protein) (Cerebral vascular amyloid peptide) (CVAP) (PreA4) (Protease nexin-II) (PN-II) [Cleaved into: N-APP; Soluble APP-alpha (S-APP-alpha); Soluble APP-beta (S-APP-beta); C99; Beta-amyloid protein 42 (Beta-APP42); Beta-amyloid protein 40 (Beta-APP40); C83; P3(42); P3(40); C80; Gamma-secretase C-terminal fragment 59 (Amyloid intracellular domain 59) (AICD-59) (AID(59)) (Gamma-CTF(59)); Gamma-secretase C-terminal fragment 57 (Amyloid intracellular domain 57) (AICD-57) (AID(57)) (Gamma-CTF(57)); Gamma-secretase C-terminal fragment 50 (Amyloid intracellular domain 50) (AICD-50) (AID(50)) (Gamma-CTF(50)); C31] | -0.81 | 0.524977722 | 0.0 |
| Q16270 | IGFBP7 | Insulin-like growth factor-binding protein 7 (IBP-7) (IGF-binding protein 7) (IGFBP-7) (IGFBP-rP1) (MAC25 protein) (PGI2-stimulating factor) (Prostacyclin-stimulating factor) (Tumor-derived adhesion factor) (TAF) | -0.31 | 0.54662553 | 0.0 |
| Q99435-3 | NELL2 | Protein kinase C-binding protein NELL2 (NEL-like protein 2) (Nel-related protein 2) | -0.61 | 0.54662553 | 0.0 |
| P09668 | CTSH | Pro-cathepsin H [Cleaved into: Cathepsin H mini chain; Cathepsin H (EC 3.4.22.16); Cathepsin H heavy chain; Cathepsin H light chain] | -0.07 | 0.554406105 | 0.0 |
| Q96PX8 | SLITRK1 | SLIT and NTRK-like protein 1 (Leucine-rich repeat-containing protein 12) | -0.92 | 0.560943588 | 0.0 |
| Q92932-3 | PTPRN2 | Receptor-type tyrosine-protein phosphatase N2 (R-PTP-N2) (EC 3.1.3.-) (EC 3.1.3.48) (Islet cell autoantigen-related protein) (IAR) (ICAAR) (Phogrin) [Cleaved into: IA-2beta60] | -0.67 | 0.562853769 | 0.0 |
| Q92820 | GGH | Gamma-glutamyl hydrolase (EC 3.4.19.9) (Conjugase) (GH) (Gamma-Glu-X carboxypeptidase) | -0.68 | 0.562853769 | 0.0 |
| Q92520 | FAM3C | Protein FAM3C (Interleukin-like EMT inducer) | -1.09 | 0.574067568 | 0.0 |
| P32119 | PRDX2 | Peroxiredoxin-2 (EC 1.11.1.15) (Natural killer cell-enhancing factor B) (NKEF-B) (PRP) (Thiol-specific antioxidant protein) (TSA) (Thioredoxin peroxidase 1) (Thioredoxin-dependent peroxide reductase 1) | 1.45 | 0.577269776 | 0.0 |
| Q9BRK5 | SDF4 | 45 kDa calcium-binding protein (Cab45) (Stromal cell-derived factor 4) (SDF-4) | -0.01 | 0.578458927 | 0.0 |
| P26038 | MSN | Moesin (Membrane-organizing extension spike protein) | 0.26 | 0.580473783 | 0.0 |
| P54756 | EPHA5 | Ephrin type-A receptor 5 (EC 2.7.10.1) (Brain-specific kinase) (EPH homology kinase 1) (EHK-1) (EPH-like kinase 7) (EK7) (hEK7) | -0.92 | 0.580842636 | 0.0 |
| Q7Z3B1 | NEGR1 | Neuronal growth regulator 1 (IgLON family member 4) | -0.70 | 0.581242359 | 0.0 |
| Q13508 | ART3 | Ecto-ADP-ribosyltransferase 3 (EC 2.4.2.31) (ADP-ribosyltransferase C2 and C3 toxin-like 3) (ARTC3) (Mono(ADP-ribosyl)transferase 3) (NAD(P)(+)--arginine ADP-ribosyltransferase 3) | -0.47 | 0.592856005 | 0.0 |
| Q13332 | PTPRS | Receptor-type tyrosine-protein phosphatase S (R-PTP-S) (EC 3.1.3.48) (Receptor-type tyrosine-protein phosphatase sigma) (R-PTP-sigma) | -0.37 | 0.595226752 | 0.0 |
| Q9NZC2 | TREM2 | Triggering receptor expressed on myeloid cells 2 (TREM-2) (Triggering receptor expressed on monocytes 2) | -0.02 | 0.596444113 | 0.0 |
| Q9HCU4 | CELSR2 | Cadherin EGF LAG seven-pass G-type receptor 2 (Cadherin family member 10) (Epidermal growth factor-like protein 2) (EGF-like protein 2) (Flamingo homolog 3) (Multiple epidermal growth factor-like domains protein 3) (Multiple EGF-like domains protein 3) | -0.72 | 0.597800493 | 0.0 |
| O15394 | NCAM2 | Neural cell adhesion molecule 2 (N-CAM-2) (NCAM-2) | -0.07 | 0.59809869 | 0.0 |
| Q13228-4 | SELENBP1 | Selenium-binding protein 1 (56 kDa selenium-binding protein) (SBP56) (SP56) | -0.28 | 0.613596988 | 0.0 |
| O60279 | SUSD5 | Sushi domain-containing protein 5 | -0.29 | 0.613596988 | 0.0 |
| Q8N126-2 | CADM3 | Cell adhesion molecule 3 (Brain immunoglobulin receptor) (Immunoglobulin superfamily member 4B) (IgSF4B) (Nectin-like protein 1) (NECL-1) (Synaptic cell adhesion molecule 3) (SynCAM3) (TSLC1-like protein 1) (TSLL1) | -0.45 | 0.613596988 | 0.0 |
| Q9UJJ9 | GNPTG | N-acetylglucosamine-1-phosphotransferase subunit gamma (GlcNAc-1-phosphotransferase subunit gamma) (UDP-N-acetylglucosamine-1-phosphotransferase subunit gamma) | 0.19 | 0.613596988 | 0.0 |
| P23471 | PTPRZ1 | Receptor-type tyrosine-protein phosphatase zeta (R-PTP-zeta) (EC 3.1.3.48) (Protein-tyrosine phosphatase receptor type Z polypeptide 1) (Protein-tyrosine phosphatase receptor type Z polypeptide 2) (R-PTP-zeta-2) | -0.23 | 0.614401866 | 0.0 |
| Q92876 | KLK6 | Kallikrein-6 (EC 3.4.21.-) (Neurosin) (Protease M) (SP59) (Serine protease 18) (Serine protease 9) (Zyme) | -0.91 | 0.620506115 | 0.0 |
| P07711 | CTSL | Cathepsin L1 (EC 3.4.22.15) (Cathepsin L) (Major excreted protein) (MEP) [Cleaved into: Cathepsin L1 heavy chain; Cathepsin L1 light chain] | 0.31 | 0.628829009 | 0.0 |
| P18065 | IGFBP2 | Insulin-like growth factor-binding protein 2 (IBP-2) (IGF-binding protein 2) (IGFBP-2) | -0.04 | 0.647313058 | 0.0 |
| P11279 | LAMP1 | Lysosome-associated membrane glycoprotein 1 (LAMP-1) (Lysosome-associated membrane protein 1) (CD107 antigen-like family member A) (CD antigen CD107a) | 0.34 | 0.653478489 | 0.0 |
| P55290-4 | CDH13 | Cadherin-13 (Heart cadherin) (H-cadherin) (P105) (Truncated cadherin) (T-cad) (T-cadherin) | -0.98 | 0.657265712 | 0.0 |
| O43493 | TGOLN2 | Trans-Golgi network integral membrane protein 2 (TGN38 homolog) (TGN46) (TGN48) (Trans-Golgi network protein TGN51) | -0.64 | 0.662226794 | 0.0 |
| Q14982-4 | OPCML | Opioid-binding protein/cell adhesion molecule (OBCAM) (OPCML) (Opioid-binding cell adhesion molecule) (IgLON family member 1) | -0.44 | 0.663906466 | 0.0 |
| Q6EMK4 | VASN | Vasorin (Protein slit-like 2) | -0.09 | 0.675510031 | 0.0 |
| P10645 | CHGA | Chromogranin-A (CgA) (Pituitary secretory protein I) (SP-I) [Cleaved into: Vasostatin-1 (Vasostatin I); Vasostatin-2 (Vasostatin II); EA-92; ES-43; Pancreastatin; SS-18; WA-8; WE-14; LF-19; Catestatin (SL21); AL-11; GV-19; GR-44; ER-37; GE-25; Serpinin-RRG; Serpinin; p-Glu serpinin precursor] | -0.41 | 0.692624168 | 0.0 |
| P51693-2 | APLP1 | Amyloid-like protein 1 (APLP) (APLP-1) [Cleaved into: C30] | -0.73 | 0.707832552 | 0.0 |
| P04216 | THY1 | Thy-1 membrane glycoprotein (CDw90) (Thy-1 antigen) (CD antigen CD90) | -0.63 | 0.708652634 | 0.0 |
| Q9Y2T3-3 | GDA | Guanine deaminase (Guanase) (Guanine aminase) (EC 3.5.4.3) (Guanine aminohydrolase) (GAH) (p51-nedasin) | -0.62 | 0.708652634 | 0.0 |
| Q8WXD2 | SCG3 | Secretogranin-3 (Secretogranin III) (SgIII) | -0.62 | 0.710326867 | 0.0 |
| P12259 | F5 | Coagulation factor V (Activated protein C cofactor) (Proaccelerin, labile factor) [Cleaved into: Coagulation factor V heavy chain; Coagulation factor V light chain] | -0.04 | 0.710613657 | 0.0 |
| P80723 | BASP1 | Brain acid soluble protein 1 (22 kDa neuronal tissue-enriched acidic protein) (Neuronal axonal membrane protein NAP-22) | -0.08 | 0.710613657 | 0.0 |
| P01034 | CST3 | Cystatin-C (Cystatin-3) (Gamma-trace) (Neuroendocrine basic polypeptide) (Post-gamma-globulin) | -0.52 | 0.712570606 | 0.0 |
| Q9NRN5 | OLFML3 | Olfactomedin-like protein 3 (HNOEL-iso) (hOLF44) | -0.42 | 0.712570606 | 0.0 |
| Q969H8 | MYDGF | Myeloid-derived growth factor (MYDGF) (Interleukin-25) (IL-25) (Stromal cell-derived growth factor SF20) | -0.39 | 0.712570606 | 0.0 |
| Q9NT99 | LRRC4B | Leucine-rich repeat-containing protein 4B (Netrin-G3 ligand) (NGL-3) | -0.09 | 0.713175796 | 0.0 |
| P17900 | GM2A | Ganglioside GM2 activator (Cerebroside sulfate activator protein) (GM2-AP) (Sphingolipid activator protein 3) (SAP-3) [Cleaved into: Ganglioside GM2 activator isoform short] | -0.73 | 0.717229511 | 0.0 |
| P02452 | COL1A1 | Collagen alpha-1(I) chain (Alpha-1 type I collagen) | -0.19 | 0.718467855 | 0.0 |
| Q01459 | CTBS | Di-N-acetylchitobiase (EC 3.2.1.-) | -0.34 | 0.721857535 | 0.0 |
| Q9NZ53 | PODXL2 | Podocalyxin-like protein 2 (Endoglycan) | -1.04 | 0.721857535 | 0.0 |
| P00338-3 | LDHA | L-lactate dehydrogenase A chain (LDH-A) (EC 1.1.1.27) (Cell proliferation-inducing gene 19 protein) (LDH muscle subunit) (LDH-M) (Renal carcinoma antigen NY-REN-59) | 0.09 | 0.732264332 | 0.0 |
| Q9Y2I2 | NTNG1 | Netrin-G1 (Laminet-1) | -1.36 | 0.732264332 | 0.0 |
| Q12860 | CNTN1 | Contactin-1 (Glycoprotein gp135) (Neural cell surface protein F3) | -0.35 | 0.738792671 | 0.0 |
| Q9P121-4 | NTM | Neurotrimin (hNT) (IgLON family member 2) | -0.43 | 0.746478987 | 0.0 |
| P19022 | CDH2 | Cadherin-2 (CDw325) (Neural cadherin) (N-cadherin) (CD antigen CD325) | -0.54 | 0.76090317 | 0.0 |
| Q99969 | RARRES2 | Retinoic acid receptor responder protein 2 (Chemerin) (RAR-responsive protein TIG2) (Tazarotene-induced gene 2 protein) | -0.77 | 0.76259533 | 0.0 |
| P05155-3 | SERPING1 | Plasma protease C1 inhibitor (C1 Inh) (C1Inh) (C1 esterase inhibitor) (C1-inhibiting factor) (Serpin G1) | -0.77 | 0.76259533 | 0.0 |
| P14618-2 | PKM | Pyruvate kinase PKM (EC 2.7.1.40) (Cytosolic thyroid hormone-binding protein) (CTHBP) (Opa-interacting protein 3) (OIP-3) (Pyruvate kinase 2/3) (Pyruvate kinase muscle isozyme) (Thyroid hormone-binding protein 1) (THBP1) (Tumor M2-PK) (p58) | -0.30 | 0.76259533 | 0.0 |
| O94985 | CLSTN1 | Calsyntenin-1 (Alcadein-alpha) (Alc-alpha) (Alzheimer-related cadherin-like protein) (Non-classical cadherin XB31alpha) [Cleaved into: Soluble Alc-alpha (SAlc-alpha); CTF1-alpha (C-terminal fragment 1-alpha)] | -0.45 | 0.763814819 | 0.0 |
| P43121 | MCAM | Cell surface glycoprotein MUC18 (Cell surface glycoprotein P1H12) (Melanoma cell adhesion molecule) (Melanoma-associated antigen A32) (Melanoma-associated antigen MUC18) (S-endo 1 endothelial-associated antigen) (CD antigen CD146) | -0.37 | 0.788345554 | 0.0 |
| Q8NFZ8 | CADM4 | Cell adhesion molecule 4 (Immunoglobulin superfamily member 4C) (IgSF4C) (Nectin-like protein 4) (NECL-4) (TSLC1-like protein 2) | -0.22 | 0.794006483 | 0.0 |
| Q8TCZ2 | CD99L2 | CD99 antigen-like protein 2 (MIC2-like protein 1) (CD antigen CD99) | -0.39 | 0.803781284 | 0.0 |
| P26992 | CNTFR | Ciliary neurotrophic factor receptor subunit alpha (CNTF receptor subunit alpha) (CNTFR-alpha) | -0.12 | 0.803781284 | 0.0 |
| P20933 | AGA | N(4)-(beta-N-acetylglucosaminyl)-L-asparaginase (EC 3.5.1.26) (Aspartylglucosaminidase) (Glycosylasparaginase) (N4-(N-acetyl-beta-glucosaminyl)-L-asparagine amidase) [Cleaved into: Glycosylasparaginase alpha chain; Glycosylasparaginase beta chain] | -0.84 | 0.81466725 | 0.0 |
| O15240 | VGF | Neurosecretory protein VGF [Cleaved into: Neuroendocrine regulatory peptide-1 (NERP-1); Neuroendocrine regulatory peptide-2 (NERP-2); Antimicrobial peptide VGF[554-577]] | -0.91 | 0.817971027 | 0.0 |
| O94919 | ENDOD1 | Endonuclease domain-containing 1 protein (EC 3.1.30.-) | -0.54 | 0.836095849 | 0.0 |
| Q9UMF0 | ICAM5 | Intercellular adhesion molecule 5 (ICAM-5) (Telencephalin) | -1.02 | 0.857145286 | 0.0 |
| P62979 | RPS27A | Ubiquitin-40S ribosomal protein S27a (Ubiquitin carboxyl extension protein 80) [Cleaved into: Ubiquitin; 40S ribosomal protein S27a (Small ribosomal subunit protein eS31)] | -0.35 | 0.857145286 | 0.0 |
| P22105-4 | TNXB | Tenascin-X (TN-X) (Hexabrachion-like protein) | -0.53 | 0.859356087 | 0.0 |
| P09211 | GSTP1 | Glutathione S-transferase P (EC 2.5.1.18) (GST class-pi) (GSTP1-1) | -0.18 | 0.863706752 | 0.0 |
| P11362-21 | FGFR1 | Fibroblast growth factor receptor 1 (FGFR-1) (EC 2.7.10.1) (Basic fibroblast growth factor receptor 1) (BFGFR) (bFGF-R-1) (Fms-like tyrosine kinase 2) (FLT-2) (N-sam) (Proto-oncogene c-Fgr) (CD antigen CD331) | -0.54 | 0.864663737 | 0.0 |
| Q12805 | EFEMP1 | EGF-containing fibulin-like extracellular matrix protein 1 (Extracellular protein S1-5) (Fibrillin-like protein) (Fibulin-3) (FIBL-3) | -0.18 | 0.864663737 | 0.0 |
| P07998 | RNASE1 | Ribonuclease pancreatic (EC 3.1.27.5) (HP-RNase) (RIB-1) (RNase UpI-1) (Ribonuclease 1) (RNase 1) (Ribonuclease A) (RNase A) | -0.70 | 0.865801368 | 0.0 |
| P0DJI9 | SAA2 | Serum amyloid A-2 protein (SAA2) | 3.19 | 0.865801368 | 0.0 |
| Q13740 | ALCAM | CD166 antigen (Activated leukocyte cell adhesion molecule) (CD antigen CD166) | -0.45 | 0.86794302 | 0.0 |
| P11021 | HSPA5 | 78 kDa glucose-regulated protein (GRP-78) (Endoplasmic reticulum lumenal Ca(2+)-binding protein grp78) (Heat shock 70 kDa protein 5) (Immunoglobulin heavy chain-binding protein) (BiP) | -0.52 | 0.874610359 | 0.0 |
| P20062 | TCN2 | Transcobalamin-2 (TC-2) (Transcobalamin II) (TC II) (TCII) | 0.23 | 0.87854759 | 0.0 |
| P33908 | MAN1A1 | Mannosyl-oligosaccharide 1,2-alpha-mannosidase IA (EC 3.2.1.113) (Man(9)-alpha-mannosidase) (Man9-mannosidase) (Mannosidase alpha class 1A member 1) (Processing alpha-1,2-mannosidase IA) (Alpha-1,2-mannosidase IA) | -0.50 | 0.888867274 | 0.0 |
| O75326 | SEMA7A | Semaphorin-7A (CDw108) (JMH blood group antigen) (John-Milton-Hargen human blood group Ag) (Semaphorin-K1) (Sema K1) (Semaphorin-L) (Sema L) (CD antigen CD108) | -0.62 | 0.905173237 | 0.0 |
| P07339 | CTSD | Cathepsin D (EC 3.4.23.5) [Cleaved into: Cathepsin D light chain; Cathepsin D heavy chain] | -0.25 | 0.914582854 | 0.0 |
| O94856 | NFASC | Neurofascin | -0.57 | 0.914582854 | 0.0 |
| P12109 | COL6A1 | Collagen alpha-1(VI) chain | -0.40 | 0.916891709 | 0.0 |
| Q8WZA1 | POMGNT1 | Protein O-linked-mannose beta-1,2-N-acetylglucosaminyltransferase 1 (POMGnT1) (EC 2.4.1.-) (UDP-GlcNAc:alpha-D-mannoside beta-1,2-N-acetylglucosaminyltransferase I.2) (GnT I.2) | -1.17 | 0.926171001 | 0.0 |
| Q9BYH1 | SEZ6L | Seizure 6-like protein | -0.46 | 0.926171001 | 0.0 |
| Q6UXD5 | SEZ6L2 | Seizure 6-like protein 2 | -1.01 | 0.929933147 | 0.0 |
| Q9NY97 | B3GNT2 | N-acetyllactosaminide beta-1,3-N-acetylglucosaminyltransferase 2 (EC 2.4.1.149) (Beta-1,3-N-acetylglucosaminyltransferase 1) (BGnT-1) (Beta-1,3-Gn-T1) (Beta3Gn-T1) (Beta-1,3-galactosyltransferase 7) (Beta-1,3-GalTase 7) (Beta3Gal-T7) (Beta3GalT7) (b3Gal-T7) (Beta-3-Gx-T7) (UDP-Gal:beta-GlcNAc beta-1,3-galactosyltransferase 7) (UDP-GlcNAc:betaGal beta-1,3-N-acetylglucosaminyltransferase 2) (BGnT-2) (Beta-1,3-Gn-T2) (Beta-1,3-N-acetylglucosaminyltransferase 2) (Beta3Gn-T2) (UDP-galactose:beta-N-acetylglucosamine beta-1,3-galactosyltransferase 7) | -0.89 | 0.929933147 | 0.0 |
| P43146 | DCC | Netrin receptor DCC (Colorectal cancer suppressor) (Immunoglobulin superfamily DCC subclass member 1) (Tumor suppressor protein DCC) | -0.94 | 0.930057191 | 0.0 |
| Q01995 | TAGLN | Transgelin (22 kDa actin-binding protein) (Protein WS3-10) (Smooth muscle protein 22-alpha) (SM22-alpha) | -0.04 | 0.930057191 | 0.0 |
| Q96FE7 | PIK3IP1 | Phosphoinositide-3-kinase-interacting protein 1 (Kringle domain-containing protein HGFL) | -0.26 | 0.930057191 | 0.0 |
| Q06418 | TYRO3 | Tyrosine-protein kinase receptor TYRO3 (EC 2.7.10.1) (Tyrosine-protein kinase BYK) (Tyrosine-protein kinase DTK) (Tyrosine-protein kinase RSE) (Tyrosine-protein kinase SKY) (Tyrosine-protein kinase TIF) | -0.49 | 0.942839636 | 0.0 |
| P17174 | GOT1 | Aspartate aminotransferase, cytoplasmic (cAspAT) (EC 2.6.1.1) (EC 2.6.1.3) (Cysteine aminotransferase, cytoplasmic) (Cysteine transaminase, cytoplasmic) (cCAT) (Glutamate oxaloacetate transaminase 1) (Transaminase A) | -0.15 | 0.942839636 | 0.0 |
| Q9P0K1 | ADAM22 | Disintegrin and metalloproteinase domain-containing protein 22 (ADAM 22) (Metalloproteinase-disintegrin ADAM22-3) (Metalloproteinase-like, disintegrin-like, and cysteine-rich protein 2) | -0.11 | 0.942839636 | 0.0 |
| P00441 | SOD1 | Superoxide dismutase [Cu-Zn] (EC 1.15.1.1) (Superoxide dismutase 1) (hSod1) | -0.53 | 0.944091576 | 0.0 |
| P99999 | CYCS | Cytochrome c | -0.69 | 0.954921451 | 0.0 |
| P23468 | PTPRD | Receptor-type tyrosine-protein phosphatase delta (Protein-tyrosine phosphatase delta) (R-PTP-delta) (EC 3.1.3.48) | -0.81 | 0.965204774 | 0.0 |
| Q12907 | LMAN2 | Vesicular integral-membrane protein VIP36 (Glycoprotein GP36b) (Lectin mannose-binding 2) (Vesicular integral-membrane protein 36) (VIP36) | -0.11 | 0.967082781 | 0.0 |
| O14594 | NCAN | Neurocan core protein (Chondroitin sulfate proteoglycan 3) | -0.21 | 0.977494474 | 0.0 |
| P04438 | IGHV2-70 | Immunoglobulin heavy variable 2-70 | 0.78 | 0.981589375 | 0.0 |
| P60174 | TPI1 | Triosephosphate isomerase (TIM) (EC 5.3.1.1) (Triose-phosphate isomerase) | -1.29 | 0.986961879 | 0.0 |
| Q9NX62 | IMPAD1 | Inositol monophosphatase 3 (IMP 3) (IMPase 3) (EC 3.1.3.25) (EC 3.1.3.7) (Golgi 3-prime phosphoadenosine 5-prime phosphate 3-prime phosphatase) (Golgi-resident PAP phosphatase) (gPAPP) (Inositol monophosphatase domain-containing protein 1) (Inositol-1(or 4)-monophosphatase 3) (Myo-inositol monophosphatase A3) | -1.07 | 0.988231512 | 0.0 |
| Q24JP5-2 | TMEM132A | Transmembrane protein 132A (HSPA5-binding protein 1) | -0.92 | 0.990068322 | 0.0 |
| P13521 | SCG2 | Secretogranin-2 (Chromogranin-C) (Secretogranin II) (SgII) [Cleaved into: Secretoneurin (SN); Manserin] | -0.84 | 0.992778931 | 0.0 |
| Q9UHG2 | PCSK1N | ProSAAS (Proprotein convertase subtilisin/kexin type 1 inhibitor) (Proprotein convertase 1 inhibitor) (pro-SAAS) [Cleaved into: KEP; Big SAAS (b-SAAS); Little SAAS (l-SAAS) (N-proSAAS); Big PEN-LEN (b-PEN-LEN) (SAAS CT(1-49)); PEN; Little LEN (l-LEN); Big LEN (b-LEN) (SAAS CT(25-40))] | -0.45 | 0.998197848 | 0.0 |
| P23515 | OMG | Oligodendrocyte-myelin glycoprotein | -0.57 | 0.998197848 | 0.0 |
| P05408 | SCG5 | Neuroendocrine protein 7B2 (Pituitary polypeptide) (Secretogranin V) (Secretogranin-5) (Secretory granule endocrine protein I) [Cleaved into: N-terminal peptide; C-terminal peptide] | -0.61 | 0.998197848 | 0.0 |
