# Supplementary material for: Proteomic changes in cerebrospinal fluid from primary central nervous system lymphoma patients are associated with protein ectodomain shedding
Source: Oncotarget. 2017 Nov 24;8(66):110118–32. doi: 10.18632/oncotarget.22654 (PMC5746369; doi:10.18632/oncotarget.22654)
Supplement: Supplementary file 3 [file oncotarget-08-110118-s003.docx]

**Supplementary Table 3: Classified PCNSL CSF proteome (306 proteins).**

| **UniProt Accession** | **Gene** | **Protein Name** |
| --- | --- | --- |
| A6NJ16 | IGHV4OR15-8 | Putative V-set and immunoglobulin domain-containing-like protein IGHV4OR15-8 |
| P62736 | ACTA2 | Actin, aortic smooth muscle (Alpha-actin-2) (Cell growth-inhibiting gene 46 protein) |
| P60709 | ACTB | Actin, cytoplasmic 1 (Beta-actin) [Cleaved into: Actin, cytoplasmic 1, N-terminally processed] |
| Q9P0K1 | ADAM22 | Disintegrin and metalloproteinase domain-containing protein 22 (ADAM 22) (Metalloproteinase-disintegrin ADAM22-3) (Metalloproteinase-like, disintegrin-like, and cysteine-rich protein 2) |
| O60241 | ADGRB2 | Adhesion G protein-coupled receptor B2 (Brain-specific angiogenesis inhibitor 2) |
| O94910 | ADGRL1 | Adhesion G protein-coupled receptor L1 (Calcium-independent alpha-latrotoxin receptor 1) (CIRL-1) (Latrophilin-1) (Lectomedin-2) |
| Q9HAR2-4 | ADGRL3 | Adhesion G protein-coupled receptor L3 (Calcium-independent alpha-latrotoxin receptor 3) (CIRL-3) (Latrophilin-3) (Lectomedin-3) |
| P20933 | AGA | N(4)-(beta-N-acetylglucosaminyl)-L-asparaginase (EC 3.5.1.26) (Aspartylglucosaminidase) (Glycosylasparaginase) (N4-(N-acetyl-beta-glucosaminyl)-L-asparagine amidase) [Cleaved into: Glycosylasparaginase alpha chain; Glycosylasparaginase beta chain] |
| O00468 | AGRN | Agrin [Cleaved into: Agrin N-terminal 110 kDa subunit; Agrin C-terminal 110 kDa subunit; Agrin C-terminal 90 kDa fragment (C90); Agrin C-terminal 22 kDa fragment (C22)] |
| P01019 | AGT | Angiotensinogen (Serpin A8) [Cleaved into: Angiotensin-1 (Angiotensin 1-10) (Angiotensin I) (Ang I); Angiotensin-2 (Angiotensin 1-8) (Angiotensin II) (Ang II); Angiotensin-3 (Angiotensin 2-8) (Angiotensin III) (Ang III) (Des-Asp[1]-angiotensin II); Angiotensin-4 (Angiotensin 3-8) (Angiotensin IV) (Ang IV); Angiotensin 1-9; Angiotensin 1-7; Angiotensin 1-5; Angiotensin 1-4] |
| Q13740 | ALCAM | CD166 antigen (Activated leukocyte cell adhesion molecule) (CD antigen CD166) |
| P09972 | ALDOC | Fructose-bisphosphate aldolase C (EC 4.1.2.13) (Brain-type aldolase) |
| P51693-2 | APLP1 | Amyloid-like protein 1 (APLP) (APLP-1) [Cleaved into: C30] |
| P06727 | APOA4 | Apolipoprotein A-IV (Apo-AIV) (ApoA-IV) (Apolipoprotein A4) |
| P02649 | APOE | Apolipoprotein E (Apo-E) |
| P05067 | APP | Amyloid beta A4 protein (ABPP) (APPI) (APP) (Alzheimer disease amyloid protein) (Amyloid precursor protein) (Beta-amyloid precursor protein) (Cerebral vascular amyloid peptide) (CVAP) (PreA4) (Protease nexin-II) (PN-II) [Cleaved into: N-APP; Soluble APP-alpha (S-APP-alpha); Soluble APP-beta (S-APP-beta); C99; Beta-amyloid protein 42 (Beta-APP42); Beta-amyloid protein 40 (Beta-APP40); C83; P3(42); P3(40); C80; Gamma-secretase C-terminal fragment 59 (Amyloid intracellular domain 59) (AICD-59) (AID(59)) (Gamma-CTF(59)); Gamma-secretase C-terminal fragment 57 (Amyloid intracellular domain 57) (AICD-57) (AID(57)) (Gamma-CTF(57)); Gamma-secretase C-terminal fragment 50 (Amyloid intracellular domain 50) (AICD-50) (AID(50)) (Gamma-CTF(50)); C31] |
| Q13508 | ART3 | Ecto-ADP-ribosyltransferase 3 (EC 2.4.2.31) (ADP-ribosyltransferase C2 and C3 toxin-like 3) (ARTC3) (Mono(ADP-ribosyl)transferase 3) (NAD(P)(+)--arginine ADP-ribosyltransferase 3) |
| Q15904 | ATP6AP1 | V-type proton ATPase subunit S1 (V-ATPase subunit S1) (Protein XAP-3) (V-ATPase Ac45 subunit) (V-ATPase S1 accessory protein) (Vacuolar proton pump subunit S1) |
| O75787 | ATP6AP2 | Renin receptor (ATPase H(+)-transporting lysosomal accessory protein 2) (ATPase H(+)-transporting lysosomal-interacting protein 2) (ER-localized type I transmembrane adaptor) (Embryonic liver differentiation factor 10) (N14F) (Renin/prorenin receptor) (Vacuolar ATP synthase membrane sector-associated protein M8-9) (ATP6M8-9) (V-ATPase M8.9 subunit) |
| P61769 | B2M | Beta-2-microglobulin [Cleaved into: Beta-2-microglobulin form pI 5.3] |
| Q9NY97 | B3GNT2 | N-acetyllactosaminide beta-1,3-N-acetylglucosaminyltransferase 2 (EC 2.4.1.149) (Beta-1,3-N-acetylglucosaminyltransferase 1) (BGnT-1) (Beta-1,3-Gn-T1) (Beta3Gn-T1) (Beta-1,3-galactosyltransferase 7) (Beta-1,3-GalTase 7) (Beta3Gal-T7) (Beta3GalT7) (b3Gal-T7) (Beta-3-Gx-T7) (UDP-Gal:beta-GlcNAc beta-1,3-galactosyltransferase 7) (UDP-GlcNAc:betaGal beta-1,3-N-acetylglucosaminyltransferase 2) (BGnT-2) (Beta-1,3-Gn-T2) (Beta-1,3-N-acetylglucosaminyltransferase 2) (Beta3Gn-T2) (UDP-galactose:beta-N-acetylglucosamine beta-1,3-galactosyltransferase 7) |
| O43505 | B4GAT1 | Beta-1,4-glucuronyltransferase 1 (EC 2.4.1.-) (I-beta-1,3-N-acetylglucosaminyltransferase) (iGnT) (N-acetyllactosaminide beta-1,3-N-acetylglucosaminyltransferase) (Poly-N-acetyllactosamine extension enzyme) (UDP-GlcNAc:betaGal beta-1,3-N-acetylglucosaminyltransferase 1) |
| P80723 | BASP1 | Brain acid soluble protein 1 (22 kDa neuronal tissue-enriched acidic protein) (Neuronal axonal membrane protein NAP-22) |
| Q96GW7 | BCAN | Brevican core protein (Brain-enriched hyaluronan-binding protein) (BEHAB) (Chondroitin sulfate proteoglycan 7) |
| P30043 | BLVRB | Flavin reductase (NADPH) (FR) (EC 1.5.1.30) (Biliverdin reductase B) (BVR-B) (EC 1.3.1.24) (Biliverdin-IX beta-reductase) (Green heme-binding protein) (GHBP) (NADPH-dependent diaphorase) (NADPH-flavin reductase) (FLR) |
| Q6UX73 | C16orf89 | UPF0764 protein C16orf89 |
| P02745 | C1QA | Complement C1q subcomponent subunit A |
| P02746 | C1QB | Complement C1q subcomponent subunit B |
| P02747 | C1QC | Complement C1q subcomponent subunit C |
| P00736 | C1R | Complement C1r subcomponent (EC 3.4.21.41) (Complement component 1 subcomponent r) [Cleaved into: Complement C1r subcomponent heavy chain; Complement C1r subcomponent light chain] |
| P0C0L4 | C4A | Complement C4-A (Acidic complement C4) (C3 and PZP-like alpha-2-macroglobulin domain-containing protein 2) [Cleaved into: Complement C4 beta chain; Complement C4-A alpha chain; C4a anaphylatoxin; C4b-A; C4d-A; Complement C4 gamma chain] |
| P04003 | C4BPA | C4b-binding protein alpha chain (C4bp) (Proline-rich protein) (PRP) |
| P00915 | CA1 | Carbonic anhydrase 1 (EC 4.2.1.1) (Carbonate dehydratase I) (Carbonic anhydrase B) (CAB) (Carbonic anhydrase I) (CA-I) |
| P22748 | CA4 | Carbonic anhydrase 4 (EC 4.2.1.1) (Carbonate dehydratase IV) (Carbonic anhydrase IV) (CA-IV) |
| P54289 | CACNA2D1 | Voltage-dependent calcium channel subunit alpha-2/delta-1 (Voltage-gated calcium channel subunit alpha-2/delta-1) [Cleaved into: Voltage-dependent calcium channel subunit alpha-2-1; Voltage-dependent calcium channel subunit delta-1] |
| Q9BY67-3 | CADM1 | Cell adhesion molecule 1 (Immunoglobulin superfamily member 4) (IgSF4) (Nectin-like protein 2) (NECL-2) (Spermatogenic immunoglobulin superfamily) (SgIgSF) (Synaptic cell adhesion molecule) (SynCAM) (Tumor suppressor in lung cancer 1) (TSLC-1) |
| Q8N3J6-3 | CADM2 | Cell adhesion molecule 2 (Immunoglobulin superfamily member 4D) (IgSF4D) (Nectin-like protein 3) (NECL-3) (Synaptic cell adhesion molecule 2) (SynCAM 2) |
| Q8N126-2 | CADM3 | Cell adhesion molecule 3 (Brain immunoglobulin receptor) (Immunoglobulin superfamily member 4B) (IgSF4B) (Nectin-like protein 1) (NECL-1) (Synaptic cell adhesion molecule 3) (SynCAM3) (TSLC1-like protein 1) (TSLL1) |
| Q8NFZ8 | CADM4 | Cell adhesion molecule 4 (Immunoglobulin superfamily member 4C) (IgSF4C) (Nectin-like protein 4) (NECL-4) (TSLC1-like protein 2) |
| P27797 | CALR | Calreticulin (CRP55) (Calregulin) (Endoplasmic reticulum resident protein 60) (ERp60) (HACBP) (grp60) |
| Q9NYX4 | CALY | Neuron-specific vesicular protein calcyon |
| Q13554 | CAMK2B | Calcium/calmodulin-dependent protein kinase type II subunit beta (CaM kinase II subunit beta) (CaMK-II subunit beta) (EC 2.7.11.17) |
| Q8WVQ1 | CANT1 | Soluble calcium-activated nucleotidase 1 (SCAN-1) (EC 3.6.1.6) (Apyrase homolog) (Putative MAPK-activating protein PM09) (Putative NF-kappa-B-activating protein 107) |
| P04040 | CAT | Catalase (EC 1.11.1.6) |
| Q86VB7-2 | CD163 | Scavenger receptor cysteine-rich type 1 protein M130 (Hemoglobin scavenger receptor) (CD antigen CD163) [Cleaved into: Soluble CD163 (sCD163)] |
| Q8TCZ2 | CD99L2 | CD99 antigen-like protein 2 (MIC2-like protein 1) (CD antigen CD99) |
| P55290-4 | CDH13 | Cadherin-13 (Heart cadherin) (H-cadherin) (P105) (Truncated cadherin) (T-cad) (T-cadherin) |
| P19022 | CDH2 | Cadherin-2 (CDw325) (Neural cadherin) (N-cadherin) (CD antigen CD325) |
| P55283 | CDH4 | Cadherin-4 (Retinal cadherin) (R-CAD) (R-cadherin) |
| P55285 | CDH6 | Cadherin-6 (Kidney cadherin) (K-cadherin) |
| Q9NZK5 | CECR1 | Adenosine deaminase CECR1 (EC 3.5.4.4) (Cat eye syndrome critical region protein 1) |
| Q9HCU4 | CELSR2 | Cadherin EGF LAG seven-pass G-type receptor 2 (Cadherin family member 10) (Epidermal growth factor-like protein 2) (EGF-like protein 2) (Flamingo homolog 3) (Multiple epidermal growth factor-like domains protein 3) (Multiple EGF-like domains protein 3) |
| Q99674-5 | CGREF1 | Cell growth regulator with EF hand domain protein 1 (Cell growth regulatory gene 11 protein) (Hydrophobestin) |
| O15335 | CHAD | Chondroadherin (Cartilage leucine-rich protein) |
| P10645 | CHGA | Chromogranin-A (CgA) (Pituitary secretory protein I) (SP-I) [Cleaved into: Vasostatin-1 (Vasostatin I); Vasostatin-2 (Vasostatin II); EA-92; ES-43; Pancreastatin; SS-18; WA-8; WE-14; LF-19; Catestatin (SL21); AL-11; GV-19; GR-44; ER-37; GE-25; Serpinin-RRG; Serpinin; p-Glu serpinin precursor] |
| P05060 | CHGB | Secretogranin-1 (Chromogranin-B) (CgB) (Secretogranin I) (SgI) [Cleaved into: PE-11; GAWK peptide; CCB peptide] |
| Q15782 | CHI3L2 | Chitinase-3-like protein 2 (Chondrocyte protein 39) (YKL-39) |
| O00533-2 | CHL1 | Neural cell adhesion molecule L1-like protein (Close homolog of L1) [Cleaved into: Processed neural cell adhesion molecule L1-like protein] |
| P05452 | CLEC3B | Tetranectin (TN) (C-type lectin domain family 3 member B) (Plasminogen kringle 4-binding protein) |
| O94985 | CLSTN1 | Calsyntenin-1 (Alcadein-alpha) (Alc-alpha) (Alzheimer-related cadherin-like protein) (Non-classical cadherin XB31alpha) [Cleaved into: Soluble Alc-alpha (SAlc-alpha); CTF1-alpha (C-terminal fragment 1-alpha)] |
| P10909-2 | CLU | Clusterin (Aging-associated gene 4 protein) (Apolipoprotein J) (Apo-J) (Complement cytolysis inhibitor) (CLI) (Complement-associated protein SP-40,40) (Ku70-binding protein 1) (NA1/NA2) (Testosterone-repressed prostate message 2) (TRPM-2) [Cleaved into: Clusterin beta chain (ApoJalpha) (Complement cytolysis inhibitor a chain); Clusterin alpha chain (ApoJbeta) (Complement cytolysis inhibitor b chain)] |
| Q96KN2 | CNDP1 | Beta-Ala-His dipeptidase (EC 3.4.13.20) (CNDP dipeptidase 1) (Carnosine dipeptidase 1) (Glutamate carboxypeptidase-like protein 2) (Serum carnosinase) |
| P26992 | CNTFR | Ciliary neurotrophic factor receptor subunit alpha (CNTF receptor subunit alpha) (CNTFR-alpha) |
| Q12860 | CNTN1 | Contactin-1 (Glycoprotein gp135) (Neural cell surface protein F3) |
| Q02246 | CNTN2 | Contactin-2 (Axonal glycoprotein TAG-1) (Axonin-1) (Transient axonal glycoprotein 1) (TAX-1) |
| Q9C0A0 | CNTNAP4 | Contactin-associated protein-like 4 (Cell recognition molecule Caspr4) |
| P39060 | COL18A1 | Collagen alpha-1(XVIII) chain [Cleaved into: Endostatin] |
| P02452 | COL1A1 | Collagen alpha-1(I) chain (Alpha-1 type I collagen) |
| P08123 | COL1A2 | Collagen alpha-2(I) chain (Alpha-2 type I collagen) |
| P02461 | COL3A1 | Collagen alpha-1(III) chain |
| P12109 | COL6A1 | Collagen alpha-1(VI) chain |
| P12111 | COL6A3 | Collagen alpha-3(VI) chain |
| Q14019 | COTL1 | Coactosin-like protein |
| P16870 | CPE | Carboxypeptidase E (CPE) (EC 3.4.17.10) (Carboxypeptidase H) (CPH) (Enkephalin convertase) (Prohormone-processing carboxypeptidase) |
| Q9Y646 | CPQ | Carboxypeptidase Q (EC 3.4.17.-) (Lysosomal dipeptidase) (Plasma glutamate carboxypeptidase) |
| Q9H3G5 | CPVL | Probable serine carboxypeptidase CPVL (EC 3.4.16.-) (Carboxypeptidase, vitellogenic-like) (Vitellogenic carboxypeptidase-like protein) (VCP-like protein) (hVLP) |
| Q9NQ79 | CRTAC1 | Cartilage acidic protein 1 (68 kDa chondrocyte-expressed protein) (CEP-68) (ASPIC) |
| P09603 | CSF1 | Macrophage colony-stimulating factor 1 (CSF-1) (M-CSF) (MCSF) (Lanimostim) [Cleaved into: Processed macrophage colony-stimulating factor 1] |
| P07333 | CSF1R | Macrophage colony-stimulating factor 1 receptor (CSF-1 receptor) (CSF-1-R) (CSF-1R) (M-CSF-R) (EC 2.7.10.1) (Proto-oncogene c-Fms) (CD antigen CD115) |
| P01034 | CST3 | Cystatin-C (Cystatin-3) (Gamma-trace) (Neuroendocrine basic polypeptide) (Post-gamma-globulin) |
| P04080 | CSTB | Cystatin-B (CPI-B) (Liver thiol proteinase inhibitor) (Stefin-B) |
| Q01459 | CTBS | Di-N-acetylchitobiase (EC 3.2.1.-) |
| P07858 | CTSB | Cathepsin B (EC 3.4.22.1) (APP secretase) (APPS) (Cathepsin B1) [Cleaved into: Cathepsin B light chain; Cathepsin B heavy chain] |
| P07339 | CTSD | Cathepsin D (EC 3.4.23.5) [Cleaved into: Cathepsin D light chain; Cathepsin D heavy chain] |
| P09668 | CTSH | Pro-cathepsin H [Cleaved into: Cathepsin H mini chain; Cathepsin H (EC 3.4.22.16); Cathepsin H heavy chain; Cathepsin H light chain] |
| P07711 | CTSL | Cathepsin L1 (EC 3.4.22.15) (Cathepsin L) (Major excreted protein) (MEP) [Cleaved into: Cathepsin L1 heavy chain; Cathepsin L1 light chain] |
| P99999 | CYCS | Cytochrome c |
| Q14118 | DAG1 | Dystroglycan (Dystrophin-associated glycoprotein 1) [Cleaved into: Alpha-dystroglycan (Alpha-DG); Beta-dystroglycan (Beta-DG)] |
| P43146 | DCC | Netrin receptor DCC (Colorectal cancer suppressor) (Immunoglobulin superfamily DCC subclass member 1) (Tumor suppressor protein DCC) |
| P07585 | DCN | Decorin (Bone proteoglycan II) (PG-S2) (PG40) |
| Q9UBP4 | DKK3 | Dickkopf-related protein 3 (Dickkopf-3) (Dkk-3) (hDkk-3) |
| Q9UHL4 | DPP7 | Dipeptidyl peptidase 2 (EC 3.4.14.2) (Dipeptidyl aminopeptidase II) (Dipeptidyl peptidase 7) (Dipeptidyl peptidase II) (DPP II) (Quiescent cell proline dipeptidase) |
| Q16610-4 | ECM1 | Extracellular matrix protein 1 (Secretory component p85) |
| O94769 | ECM2 | Extracellular matrix protein 2 (Matrix glycoprotein SC1/ECM2) |
| Q12805 | EFEMP1 | EGF-containing fibulin-like extracellular matrix protein 1 (Extracellular protein S1-5) (Fibrillin-like protein) (Fibulin-3) (FIBL-3) |
| P52799 | EFNB2 | Ephrin-B2 (EPH-related receptor tyrosine kinase ligand 5) (LERK-5) (HTK ligand) (HTK-L) |
| O94919 | ENDOD1 | Endonuclease domain-containing 1 protein (EC 3.1.30.-) |
| P06733 | ENO1 | Alpha-enolase (EC 4.2.1.11) (2-phospho-D-glycerate hydro-lyase) (C-myc promoter-binding protein) (Enolase 1) (MBP-1) (MPB-1) (Non-neural enolase) (NNE) (Phosphopyruvate hydratase) (Plasminogen-binding protein) |
| Q9UJA9 | ENPP5 | Ectonucleotide pyrophosphatase/phosphodiesterase family member 5 (E-NPP 5) (NPP-5) (EC 3.1.-.-) |
| P54764 | EPHA4 | Ephrin type-A receptor 4 (EC 2.7.10.1) (EPH-like kinase 8) (EK8) (hEK8) (Tyrosine-protein kinase TYRO1) (Tyrosine-protein kinase receptor SEK) |
| P54756 | EPHA5 | Ephrin type-A receptor 5 (EC 2.7.10.1) (Brain-specific kinase) (EPH homology kinase 1) (EHK-1) (EPH-like kinase 7) (EK7) (hEK7) |
| Q9UBQ6 | EXTL2 | Exostosin-like 2 (EC 2.4.1.223) (Alpha-1,4-N-acetylhexosaminyltransferase EXTL2) (Alpha-GalNAcT EXTL2) (EXT-related protein 2) (Glucuronyl-galactosyl-proteoglycan 4-alpha-N-acetylglucosaminyltransferase) [Cleaved into: Processed exostosin-like 2] |
| P12259 | F5 | Coagulation factor V (Activated protein C cofactor) (Proaccelerin, labile factor) [Cleaved into: Coagulation factor V heavy chain; Coagulation factor V light chain] |
| Q92520 | FAM3C | Protein FAM3C (Interleukin-like EMT inducer) |
| Q9NYQ8 | FAT2 | Protocadherin Fat 2 (hFat2) (Cadherin family member 8) (Multiple epidermal growth factor-like domains protein 1) (Multiple EGF-like domains protein 1) |
| P23142 | FBLN1 | Fibulin-1 (FIBL-1) |
| P23142-4 | FBLN1 | Fibulin-1 (FIBL-1) |
| Q9Y6R7 | FCGBP | IgGFc-binding protein (Fcgamma-binding protein antigen) (FcgammaBP) |
| P11362-21 | FGFR1 | Fibroblast growth factor receptor 1 (FGFR-1) (EC 2.7.10.1) (Basic fibroblast growth factor receptor 1) (BFGFR) (bFGF-R-1) (Fms-like tyrosine kinase 2) (FLT-2) (N-sam) (Proto-oncogene c-Fgr) (CD antigen CD331) |
| Q06828 | FMOD | Fibromodulin (FM) (Collagen-binding 59 kDa protein) (Keratan sulfate proteoglycan fibromodulin) (KSPG fibromodulin) |
| Q12841 | FSTL1 | Follistatin-related protein 1 (Follistatin-like protein 1) |
| Q6MZW2 | FSTL4 | Follistatin-related protein 4 (Follistatin-like protein 4) |
| P04066 | FUCA1 | Tissue alpha-L-fucosidase (EC 3.2.1.51) (Alpha-L-fucosidase I) (Alpha-L-fucoside fucohydrolase 1) (Alpha-L-fucosidase 1) |
| Q9BTY2 | FUCA2 | Plasma alpha-L-fucosidase (EC 3.2.1.51) (Alpha-L-fucoside fucohydrolase 2) (Alpha-L-fucosidase 2) |
| P17677-2 | GAP43 | Neuromodulin (Axonal membrane protein GAP-43) (Growth-associated protein 43) (Neural phosphoprotein B-50) (pp46) |
| P04406 | GAPDH | Glyceraldehyde-3-phosphate dehydrogenase (GAPDH) (EC 1.2.1.12) (Peptidyl-cysteine S-nitrosylase GAPDH) (EC 2.6.99.-) |
| Q14393 | GAS6 | Growth arrest-specific protein 6 (GAS-6) (AXL receptor tyrosine kinase ligand) |
| Q9Y2T3-3 | GDA | Guanine deaminase (Guanase) (Guanine aminase) (EC 3.5.4.3) (Guanine aminohydrolase) (GAH) (p51-nedasin) |
| Q92820 | GGH | Gamma-glutamyl hydrolase (EC 3.4.19.9) (Conjugase) (GH) (Gamma-Glu-X carboxypeptidase) |
| Q6ZMI3 | GLDN | Gliomedin [Cleaved into: Gliomedin shedded ectodomain] |
| P17900 | GM2A | Ganglioside GM2 activator (Cerebroside sulfate activator protein) (GM2-AP) (Sphingolipid activator protein 3) (SAP-3) [Cleaved into: Ganglioside GM2 activator isoform short] |
| Q9UJJ9 | GNPTG | N-acetylglucosamine-1-phosphotransferase subunit gamma (GlcNAc-1-phosphotransferase subunit gamma) (UDP-N-acetylglucosamine-1-phosphotransferase subunit gamma) |
| O00461 | GOLIM4 | Golgi integral membrane protein 4 (Golgi integral membrane protein, cis) (GIMPc) (Golgi phosphoprotein 4) (Golgi-localized phosphoprotein of 130 kDa) (Golgi phosphoprotein of 130 kDa) |
| Q8NBJ4 | GOLM1 | Golgi membrane protein 1 (Golgi membrane protein GP73) (Golgi phosphoprotein 2) |
| P17174 | GOT1 | Aspartate aminotransferase, cytoplasmic (cAspAT) (EC 2.6.1.1) (EC 2.6.1.3) (Cysteine aminotransferase, cytoplasmic) (Cysteine transaminase, cytoplasmic) (cCAT) (Glutamate oxaloacetate transaminase 1) (Transaminase A) |
| Q14956 | GPNMB | Transmembrane glycoprotein NMB (Transmembrane glycoprotein HGFIN) |
| P22352 | GPX3 | Glutathione peroxidase 3 (GPx-3) (GSHPx-3) (EC 1.11.1.9) (Extracellular glutathione peroxidase) (Plasma glutathione peroxidase) (GPx-P) (GSHPx-P) |
| P48058 | GRIA4 | Glutamate receptor 4 (GluR-4) (GluR4) (AMPA-selective glutamate receptor 4) (GluR-D) (Glutamate receptor ionotropic, AMPA 4) (GluA4) |
| P09211 | GSTP1 | Glutathione S-transferase P (EC 2.5.1.18) (GST class-pi) (GSTP1-1) |
| P69905 | HBA1; HBA2 | Hemoglobin subunit alpha (Alpha-globin) (Hemoglobin alpha chain) |
| P68871 | HBB | Hemoglobin subunit beta (Beta-globin) (Hemoglobin beta chain) [Cleaved into: LVV-hemorphin-7; Spinorphin] |
| P07686 | HEXB | Beta-hexosaminidase subunit beta (EC 3.2.1.52) (Beta-N-acetylhexosaminidase subunit beta) (Hexosaminidase subunit B) (Cervical cancer proto-oncogene 7 protein) (HCC-7) (N-acetyl-beta-glucosaminidase subunit beta) [Cleaved into: Beta-hexosaminidase subunit beta chain B; Beta-hexosaminidase subunit beta chain A] |
| P30479 | HLA-B | HLA class I histocompatibility antigen, B-41 alpha chain (Bw-41) (MHC class I antigen B*41) |
| P01911 | HLA-DRB1 | HLA class II histocompatibility antigen, DRB1-15 beta chain (DW2.2/DR2.2) (MHC class II antigen DRB1*15) |
| P00738 | HP | Haptoglobin (Zonulin) [Cleaved into: Haptoglobin alpha chain; Haptoglobin beta chain] |
| P00739-2 | HPR | Haptoglobin-related protein |
| P11021 | HSPA5 | 78 kDa glucose-regulated protein (GRP-78) (Endoplasmic reticulum lumenal Ca(2+)-binding protein grp78) (Heat shock 70 kDa protein 5) (Immunoglobulin heavy chain-binding protein) (BiP) |
| P98160 | HSPG2 | Basement membrane-specific heparan sulfate proteoglycan core protein (HSPG) (Perlecan) (PLC) [Cleaved into: Endorepellin; LG3 peptide] |
| Q92743 | HTRA1 | Serine protease HTRA1 (EC 3.4.21.-) (High-temperature requirement A serine peptidase 1) (L56) (Serine protease 11) |
| Q9Y4L1 | HYOU1 | Hypoxia up-regulated protein 1 (150 kDa oxygen-regulated protein) (ORP-150) (170 kDa glucose-regulated protein) (GRP-170) |
| Q9UMF0 | ICAM5 | Intercellular adhesion molecule 5 (ICAM-5) (Telencephalin) |
| O75144-2 | ICOSLG | ICOS ligand (B7 homolog 2) (B7-H2) (B7-like protein Gl50) (B7-related protein 1) (B7RP-1) (CD antigen CD275) |
| P22304 | IDS | Iduronate 2-sulfatase (EC 3.1.6.13) (Alpha-L-iduronate sulfate sulfatase) (Idursulfase) [Cleaved into: Iduronate 2-sulfatase 42 kDa chain; Iduronate 2-sulfatase 14 kDa chain] |
| P35858-2 | IGFALS | Insulin-like growth factor-binding protein complex acid labile subunit (ALS) |
| P18065 | IGFBP2 | Insulin-like growth factor-binding protein 2 (IBP-2) (IGF-binding protein 2) (IGFBP-2) |
| P17936-2 | IGFBP3 | Insulin-like growth factor-binding protein 3 (IBP-3) (IGF-binding protein 3) (IGFBP-3) |
| P22692 | IGFBP4 | Insulin-like growth factor-binding protein 4 (IBP-4) (IGF-binding protein 4) (IGFBP-4) |
| Q16270 | IGFBP7 | Insulin-like growth factor-binding protein 7 (IBP-7) (IGF-binding protein 7) (IGFBP-7) (IGFBP-rP1) (MAC25 protein) (PGI2-stimulating factor) (Prostacyclin-stimulating factor) (Tumor-derived adhesion factor) (TAF) |
| P01861 | IGHG4 | Immunoglobulin heavy constant gamma 4 (Ig gamma-4 chain C region) |
| Q969P0 | IGSF8 | Immunoglobulin superfamily member 8 (IgSF8) (CD81 partner 3) (Glu-Trp-Ile EWI motif-containing protein 2) (EWI-2) (Keratinocytes-associated transmembrane protein 4) (KCT-4) (LIR-D1) (Prostaglandin regulatory-like protein) (PGRL) (CD antigen CD316) |
| P40189 | IL6ST | Interleukin-6 receptor subunit beta (IL-6 receptor subunit beta) (IL-6R subunit beta) (IL-6R-beta) (IL-6RB) (CDw130) (Interleukin-6 signal transducer) (Membrane glycoprotein 130) (gp130) (Oncostatin-M receptor subunit alpha) (CD antigen CD130) |
| Q9NX62 | IMPAD1 | Inositol monophosphatase 3 (IMP 3) (IMPase 3) (EC 3.1.3.25) (EC 3.1.3.7) (Golgi 3-prime phosphoadenosine 5-prime phosphate 3-prime phosphatase) (Golgi-resident PAP phosphatase) (gPAPP) (Inositol monophosphatase domain-containing protein 1) (Inositol-1(or 4)-monophosphatase 3) (Myo-inositol monophosphatase A3) |
| O14498 | ISLR | Immunoglobulin superfamily containing leucine-rich repeat protein |
| Q86UX2 | ITIH5 | Inter-alpha-trypsin inhibitor heavy chain H5 (ITI heavy chain H5) (ITI-HC5) (Inter-alpha-inhibitor heavy chain 5) |
| Q9Y287 | ITM2B | Integral membrane protein 2B (Immature BRI2) (imBRI2) (Protein E25B) (Transmembrane protein BRI) (Bri) [Cleaved into: BRI2, membrane form (Mature BRI2) (mBRI2); BRI2 intracellular domain (BRI2 ICD); BRI2C, soluble form; Bri23 peptide (Bri2-23) (ABri23) (C-terminal peptide) (P23 peptide)] |
| Q92876 | KLK6 | Kallikrein-6 (EC 3.4.21.-) (Neurosin) (Protease M) (SP59) (Serine protease 18) (Serine protease 9) (Zyme) |
| P11279 | LAMP1 | Lysosome-associated membrane glycoprotein 1 (LAMP-1) (Lysosome-associated membrane protein 1) (CD107 antigen-like family member A) (CD antigen CD107a) |
| P13473-3 | LAMP2 | Lysosome-associated membrane glycoprotein 2 (LAMP-2) (Lysosome-associated membrane protein 2) (CD107 antigen-like family member B) (LGP-96) (CD antigen CD107b) |
| P18428 | LBP | Lipopolysaccharide-binding protein (LBP) |
| P04180 | LCAT | Phosphatidylcholine-sterol acyltransferase (EC 2.3.1.43) (Lecithin-cholesterol acyltransferase) (Phospholipid-cholesterol acyltransferase) |
| P00338-3 | LDHA | L-lactate dehydrogenase A chain (LDH-A) (EC 1.1.1.27) (Cell proliferation-inducing gene 19 protein) (LDH muscle subunit) (LDH-M) (Renal carcinoma antigen NY-REN-59) |
| P07195 | LDHB | L-lactate dehydrogenase B chain (LDH-B) (EC 1.1.1.27) (LDH heart subunit) (LDH-H) (Renal carcinoma antigen NY-REN-46) |
| Q08380 | LGALS3BP | Galectin-3-binding protein (Basement membrane autoantigen p105) (Lectin galactoside-binding soluble 3-binding protein) (Mac-2-binding protein) (MAC2BP) (Mac-2 BP) (Tumor-associated antigen 90K) |
| Q96FE5 | LINGO1 | Leucine-rich repeat and immunoglobulin-like domain-containing nogo receptor-interacting protein 1 (Leucine-rich repeat and immunoglobulin domain-containing protein 1) (Leucine-rich repeat neuronal protein 1) (Leucine-rich repeat neuronal protein 6A) |
| Q12907 | LMAN2 | Vesicular integral-membrane protein VIP36 (Glycoprotein GP36b) (Lectin mannose-binding 2) (Vesicular integral-membrane protein 36) (VIP36) |
| Q07954 | LRP1 | Prolow-density lipoprotein receptor-related protein 1 (LRP-1) (Alpha-2-macroglobulin receptor) (A2MR) (Apolipoprotein E receptor) (APOER) (CD antigen CD91) [Cleaved into: Low-density lipoprotein receptor-related protein 1 85 kDa subunit (LRP-85); Low-density lipoprotein receptor-related protein 1 515 kDa subunit (LRP-515); Low-density lipoprotein receptor-related protein 1 intracellular domain (LRPICD)] |
| Q9NT99 | LRRC4B | Leucine-rich repeat-containing protein 4B (Netrin-G3 ligand) (NGL-3) |
| Q13449 | LSAMP | Limbic system-associated membrane protein (LSAMP) (IgLON family member 3) |
| P33908 | MAN1A1 | Mannosyl-oligosaccharide 1,2-alpha-mannosidase IA (EC 3.2.1.113) (Man(9)-alpha-mannosidase) (Man9-mannosidase) (Mannosidase alpha class 1A member 1) (Processing alpha-1,2-mannosidase IA) (Alpha-1,2-mannosidase IA) |
| P49641 | MAN2A2 | Alpha-mannosidase 2x (EC 3.2.1.114) (Alpha-mannosidase IIx) (Man IIx) (Mannosidase alpha class 2A member 2) (Mannosyl-oligosaccharide 1,3-1,6-alpha-mannosidase) |
| P48740-2 | MASP1 | Mannan-binding lectin serine protease 1 (EC 3.4.21.-) (Complement factor MASP-3) (Complement-activating component of Ra-reactive factor) (Mannose-binding lectin-associated serine protease 1) (MASP-1) (Mannose-binding protein-associated serine protease) (Ra-reactive factor serine protease p100) (RaRF) (Serine protease 5) [Cleaved into: Mannan-binding lectin serine protease 1 heavy chain; Mannan-binding lectin serine protease 1 light chain] |
| P02144 | MB | Myoglobin |
| P43121 | MCAM | Cell surface glycoprotein MUC18 (Cell surface glycoprotein P1H12) (Melanoma cell adhesion molecule) (Melanoma-associated antigen A32) (Melanoma-associated antigen MUC18) (S-endo 1 endothelial-associated antigen) (CD antigen CD146) |
| P40925-3 | MDH1 | Malate dehydrogenase, cytoplasmic (EC 1.1.1.37) (Cytosolic malate dehydrogenase) (Diiodophenylpyruvate reductase) (EC 1.1.1.96) |
| Q7Z7M0 | MEGF8 | Multiple epidermal growth factor-like domains protein 8 (Multiple EGF-like domains protein 8) (Epidermal growth factor-like protein 4) (EGF-like protein 4) |
| P55083-2 | MFAP4 | Microfibril-associated glycoprotein 4 |
| P08253 | MMP2 | 72 kDa type IV collagenase (EC 3.4.24.24) (72 kDa gelatinase) (Gelatinase A) (Matrix metalloproteinase-2) (MMP-2) (TBE-1) [Cleaved into: PEX] |
| P22897 | MRC1 | Macrophage mannose receptor 1 (MMR) (C-type lectin domain family 13 member D) (C-type lectin domain family 13 member D-like) (Human mannose receptor) (hMR) (Macrophage mannose receptor 1-like protein 1) (CD antigen CD206) |
| Q13421-2 | MSLN | Mesothelin (CAK1 antigen) (Pre-pro-megakaryocyte-potentiating factor) [Cleaved into: Megakaryocyte-potentiating factor (MPF); Mesothelin, cleaved form] |
| P26038 | MSN | Moesin (Membrane-organizing extension spike protein) |
| Q969H8 | MYDGF | Myeloid-derived growth factor (MYDGF) (Interleukin-25) (IL-25) (Stromal cell-derived growth factor SF20) |
| O15394 | NCAM2 | Neural cell adhesion molecule 2 (N-CAM-2) (NCAM-2) |
| O14594 | NCAN | Neurocan core protein (Chondroitin sulfate proteoglycan 3) |
| Q7Z3B1 | NEGR1 | Neuronal growth regulator 1 (IgLON family member 4) |
| Q99435-3 | NELL2 | Protein kinase C-binding protein NELL2 (NEL-like protein 2) (Nel-related protein 2) |
| Q92859 | NEO1 | Neogenin (Immunoglobulin superfamily DCC subclass member 2) |
| O94856 | NFASC | Neurofascin |
| P14543 | NID1 | Nidogen-1 (NID-1) (Entactin) |
| Q14112 | NID2 | Nidogen-2 (NID-2) (Osteonidogen) |
| P61916 | NPC2 | Epididymal secretory protein E1 (Human epididymis-specific protein 1) (He1) (Niemann-Pick disease type C2 protein) |
| Q15818 | NPTX1 | Neuronal pentraxin-1 (NP1) (Neuronal pentraxin I) (NP-I) |
| P47972 | NPTX2 | Neuronal pentraxin-2 (NP2) (Neuronal pentraxin II) (NP-II) |
| O95502 | NPTXR | Neuronal pentraxin receptor |
| Q92823-5 | NRCAM | Neuronal cell adhesion molecule (Nr-CAM) (Neuronal surface protein Bravo) (hBravo) (NgCAM-related cell adhesion molecule) (Ng-CAM-related) |
| O14786 | NRP1 | Neuropilin-1 (Vascular endothelial cell growth factor 165 receptor) (CD antigen CD304) |
| O60462 | NRP2 | Neuropilin-2 (Vascular endothelial cell growth factor 165 receptor 2) |
| Q9ULB1-3 | NRXN1 | Neurexin-1 (Neurexin I-alpha) (Neurexin-1-alpha) |
| Q9P2S2 | NRXN2 | Neurexin-2 (Neurexin II-alpha) (Neurexin-2-alpha) |
| Q9Y4C0-4 | NRXN3 | Neurexin-3 (Neurexin III-alpha) (Neurexin-3-alpha) |
| Q9P121-4 | NTM | Neurotrimin (hNT) (IgLON family member 2) |
| Q9Y2I2 | NTNG1 | Netrin-G1 (Laminet-1) |
| Q02818 | NUCB1 | Nucleobindin-1 (CALNUC) |
| P20774 | OGN | Mimecan (Osteoglycin) (Osteoinductive factor) (OIF) |
| Q9NRN5 | OLFML3 | Olfactomedin-like protein 3 (HNOEL-iso) (hOLF44) |
| Q99983 | OMD | Osteomodulin (Keratan sulfate proteoglycan osteomodulin) (KSPG osteomodulin) (Osteoadherin) (OSAD) |
| P23515 | OMG | Oligodendrocyte-myelin glycoprotein |
| Q14982-4 | OPCML | Opioid-binding protein/cell adhesion molecule (OBCAM) (OPCML) (Opioid-binding cell adhesion molecule) (IgLON family member 1) |
| O14917 | PCDH17 | Protocadherin-17 (Protocadherin-68) |
| Q9Y5I4 | PCDHAC2 | Protocadherin alpha-C2 (PCDH-alpha-C2) |
| Q15113 | PCOLCE | Procollagen C-endopeptidase enhancer 1 (Procollagen COOH-terminal proteinase enhancer 1) (PCPE-1) (Procollagen C-proteinase enhancer 1) (Type 1 procollagen C-proteinase enhancer protein) (Type I procollagen COOH-terminal proteinase enhancer) |
| Q9UHG2 | PCSK1N | ProSAAS (Proprotein convertase subtilisin/kexin type 1 inhibitor) (Proprotein convertase 1 inhibitor) (pro-SAAS) [Cleaved into: KEP; Big SAAS (b-SAAS); Little SAAS (l-SAAS) (N-proSAAS); Big PEN-LEN (b-PEN-LEN) (SAAS CT(1-49)); PEN; Little LEN (l-LEN); Big LEN (b-LEN) (SAAS CT(25-40))] |
| Q96S96 | PEBP4 | Phosphatidylethanolamine-binding protein 4 (PEBP-4) (hPEBP4) (Protein cousin-of-RKIP 1) |
| P01210 | PENK | Proenkephalin-A [Cleaved into: Synenkephalin; Met-enkephalin (Opioid growth factor) (OGF); PENK(114-133); PENK(143-183); Met-enkephalin-Arg-Gly-Leu; Leu-enkephalin; PENK(237-258); Met-enkephalin-Arg-Phe] |
| P00558 | PGK1 | Phosphoglycerate kinase 1 (EC 2.7.2.3) (Cell migration-inducing gene 10 protein) (Primer recognition protein 2) (PRP 2) |
| Q96FE7 | PIK3IP1 | Phosphoinositide-3-kinase-interacting protein 1 (Kringle domain-containing protein HGFL) |
| P14618-2 | PKM | Pyruvate kinase PKM (EC 2.7.1.40) (Cytosolic thyroid hormone-binding protein) (CTHBP) (Opa-interacting protein 3) (OIP-3) (Pyruvate kinase 2/3) (Pyruvate kinase muscle isozyme) (Thyroid hormone-binding protein 1) (THBP1) (Tumor M2-PK) (p58) |
| Q6UX71 | PLXDC2 | Plexin domain-containing protein 2 (Tumor endothelial marker 7-related protein) |
| Q9NZ53 | PODXL2 | Podocalyxin-like protein 2 (Endoglycan) |
| Q8WZA1 | POMGNT1 | Protein O-linked-mannose beta-1,2-N-acetylglucosaminyltransferase 1 (POMGnT1) (EC 2.4.1.-) (UDP-GlcNAc:alpha-D-mannoside beta-1,2-N-acetylglucosaminyltransferase I.2) (GnT I.2) |
| P23284 | PPIB | Peptidyl-prolyl cis-trans isomerase B (PPIase B) (EC 5.2.1.8) (CYP-S1) (Cyclophilin B) (Rotamase B) (S-cyclophilin) (SCYLP) |
| Q96NZ9-3 | PRAP1 | Proline-rich acidic protein 1 (Epididymis tissue protein Li 178) (Uterine-specific proline-rich acidic protein) |
| P32119 | PRDX2 | Peroxiredoxin-2 (EC 1.11.1.15) (Natural killer cell-enhancing factor B) (NKEF-B) (PRP) (Thiol-specific antioxidant protein) (TSA) (Thioredoxin peroxidase 1) (Thioredoxin-dependent peroxide reductase 1) |
| P51888 | PRELP | Prolargin (Proline-arginine-rich end leucine-rich repeat protein) |
| Q92954 | PRG4 | Proteoglycan 4 (Lubricin) (Megakaryocyte-stimulating factor) (Superficial zone proteoglycan) [Cleaved into: Proteoglycan 4 C-terminal part] |
| P14314 | PRKCSH | Glucosidase 2 subunit beta (80K-H protein) (Glucosidase II subunit beta) (Protein kinase C substrate 60.1 kDa protein heavy chain) (PKCSH) |
| P04156 | PRNP | Major prion protein (PrP) (ASCR) (PrP27-30) (PrP33-35C) (CD antigen CD230) |
| Q5FWE3 | PRRT3 | Proline-rich transmembrane protein 3 |
| P41222 | PTGDS | Prostaglandin-H2 D-isomerase (EC 5.3.99.2) (Beta-trace protein) (Cerebrin-28) (Glutathione-independent PGD synthase) (Lipocalin-type prostaglandin-D synthase) (Prostaglandin-D2 synthase) (PGD2 synthase) (PGDS) (PGDS2) |
| P23468 | PTPRD | Receptor-type tyrosine-protein phosphatase delta (Protein-tyrosine phosphatase delta) (R-PTP-delta) (EC 3.1.3.48) |
| P10586 | PTPRF | Receptor-type tyrosine-protein phosphatase F (EC 3.1.3.48) (Leukocyte common antigen related) (LAR) |
| P23470 | PTPRG | Receptor-type tyrosine-protein phosphatase gamma (Protein-tyrosine phosphatase gamma) (R-PTP-gamma) (EC 3.1.3.48) |
| Q16849 | PTPRN | Receptor-type tyrosine-protein phosphatase-like N (R-PTP-N) (Islet cell antigen 512) (ICA 512) (Islet cell autoantigen 3) (PTP IA-2) [Cleaved into: ICA512-N-terminal fragment (ICA512-NTF); ICA512-transmembrane fragment (ICA512-TMF); ICA512-cleaved cytosolic fragment (ICA512-CCF)] |
| Q92932-3 | PTPRN2 | Receptor-type tyrosine-protein phosphatase N2 (R-PTP-N2) (EC 3.1.3.-) (EC 3.1.3.48) (Islet cell autoantigen-related protein) (IAR) (ICAAR) (Phogrin) [Cleaved into: IA-2beta60] |
| Q13332 | PTPRS | Receptor-type tyrosine-protein phosphatase S (R-PTP-S) (EC 3.1.3.48) (Receptor-type tyrosine-protein phosphatase sigma) (R-PTP-sigma) |
| P23471 | PTPRZ1 | Receptor-type tyrosine-protein phosphatase zeta (R-PTP-zeta) (EC 3.1.3.48) (Protein-tyrosine phosphatase receptor type Z polypeptide 1) (Protein-tyrosine phosphatase receptor type Z polypeptide 2) (R-PTP-zeta-2) |
| P15151 | PVR | Poliovirus receptor (Nectin-like protein 5) (NECL-5) (CD antigen CD155) |
| P09417 | QDPR | Dihydropteridine reductase (EC 1.5.1.34) (HDHPR) (Quinoid dihydropteridine reductase) (Short chain dehydrogenase/reductase family 33C member 1) |
| Q16769 | QPCT | Glutaminyl-peptide cyclotransferase (EC 2.3.2.5) (Glutaminyl cyclase) (QC) (sQC) (Glutaminyl-tRNA cyclotransferase) (Glutamyl cyclase) (EC) |
| O00391 | QSOX1 | Sulfhydryl oxidase 1 (hQSOX) (EC 1.8.3.2) (Quiescin Q6) |
| Q99969 | RARRES2 | Retinoic acid receptor responder protein 2 (Chemerin) (RAR-responsive protein TIG2) (Tazarotene-induced gene 2 protein) |
| P78509 | RELN | Reelin (EC 3.4.21.-) |
| Q96B86-4 | RGMA | Repulsive guidance molecule A (RGM domain family member A) |
| Q6NW40 | RGMB | RGM domain family member B (DRG11-responsive axonal guidance and outgrowth of neurite) (DRAGON) |
| P07998 | RNASE1 | Ribonuclease pancreatic (EC 3.1.27.5) (HP-RNase) (RIB-1) (RNase UpI-1) (Ribonuclease 1) (RNase 1) (Ribonuclease A) (RNase A) |
| O00584 | RNASET2 | Ribonuclease T2 (EC 3.1.27.-) (Ribonuclease 6) |
| Q9Y6N7-2 | ROBO1 | Roundabout homolog 1 (Deleted in U twenty twenty) (H-Robo-1) |
| P62979 | RPS27A | Ubiquitin-40S ribosomal protein S27a (Ubiquitin carboxyl extension protein 80) [Cleaved into: Ubiquitin; 40S ribosomal protein S27a (Small ribosomal subunit protein eS31)] |
| Q9BZR6 | RTN4R | Reticulon-4 receptor (Nogo receptor) (NgR) (Nogo-66 receptor) |
| Q86UN3 | RTN4RL2 | Reticulon-4 receptor-like 2 (Nogo receptor-like 3) (Nogo-66 receptor homolog 1) (Nogo-66 receptor-related protein 2) (NgR2) |
| P0DJI8 | SAA1 | Serum amyloid A-1 protein (SAA) [Cleaved into: Amyloid protein A (Amyloid fibril protein AA); Serum amyloid protein A(2-104); Serum amyloid protein A(3-104); Serum amyloid protein A(2-103); Serum amyloid protein A(2-102); Serum amyloid protein A(4-101)] |
| P0DJI9 | SAA2 | Serum amyloid A-2 protein (SAA2) |
| P35542 | SAA4 | Serum amyloid A-4 protein (Constitutively expressed serum amyloid A protein) (C-SAA) |
| P13521 | SCG2 | Secretogranin-2 (Chromogranin-C) (Secretogranin II) (SgII) [Cleaved into: Secretoneurin (SN); Manserin] |
| Q8WXD2 | SCG3 | Secretogranin-3 (Secretogranin III) (SgIII) |
| P05408 | SCG5 | Neuroendocrine protein 7B2 (Pituitary polypeptide) (Secretogranin V) (Secretogranin-5) (Secretory granule endocrine protein I) [Cleaved into: N-terminal peptide; C-terminal peptide] |
| Q9BRK5 | SDF4 | 45 kDa calcium-binding protein (Cab45) (Stromal cell-derived factor 4) (SDF-4) |
| Q13228-4 | SELENBP1 | Selenium-binding protein 1 (56 kDa selenium-binding protein) (SBP56) (SP56) |
| Q8WWX9 | SELENOM | Selenoprotein M (SelM) |
| Q9H2E6-2 | SEMA6A | Semaphorin-6A (Semaphorin VIA) (Sema VIA) (Semaphorin-6A-1) (SEMA6A-1) |
| O75326 | SEMA7A | Semaphorin-7A (CDw108) (JMH blood group antigen) (John-Milton-Hargen human blood group Ag) (Semaphorin-K1) (Sema K1) (Semaphorin-L) (Sema L) (CD antigen CD108) |
| P36955 | SERPINF1 | Pigment epithelium-derived factor (PEDF) (Cell proliferation-inducing gene 35 protein) (EPC-1) (Serpin F1) |
| P05155-3 | SERPING1 | Plasma protease C1 inhibitor (C1 Inh) (C1Inh) (C1 esterase inhibitor) (C1-inhibiting factor) (Serpin G1) |
| Q53EL9 | SEZ6 | Seizure protein 6 homolog (SEZ-6) (hSEZ-6) |
| Q9BYH1 | SEZ6L | Seizure 6-like protein |
| Q6UXD5 | SEZ6L2 | Seizure 6-like protein 2 |
| O43556-4 | SGCE | Epsilon-sarcoglycan (Epsilon-SG) |
| P04278 | SHBG | Sex hormone-binding globulin (SHBG) (Sex steroid-binding protein) (SBP) (Testis-specific androgen-binding protein) (ABP) (Testosterone-estradiol-binding globulin) (TeBG) (Testosterone-estrogen-binding globulin) |
| Q9HAT2 | SIAE | Sialate O-acetylesterase (EC 3.1.1.53) (H-Lse) (Sialic acid-specific 9-O-acetylesterase) |
| P78324-2 | SIRPA | Tyrosine-protein phosphatase non-receptor type substrate 1 (SHP substrate 1) (SHPS-1) (Brain Ig-like molecule with tyrosine-based activation motifs) (Bit) (CD172 antigen-like family member A) (Inhibitory receptor SHPS-1) (Macrophage fusion receptor) (MyD-1 antigen) (Signal-regulatory protein alpha-1) (Sirp-alpha-1) (Signal-regulatory protein alpha-2) (Sirp-alpha-2) (Signal-regulatory protein alpha-3) (Sirp-alpha-3) (p84) (CD antigen CD172a) |
| Q9ULF5 | SLC39A10 | Zinc transporter ZIP10 (Solute carrier family 39 member 10) (Zrt- and Irt-like protein 10) (ZIP-10) |
| Q96PX8 | SLITRK1 | SLIT and NTRK-like protein 1 (Leucine-rich repeat-containing protein 12) |
| P00441 | SOD1 | Superoxide dismutase [Cu-Zn] (EC 1.15.1.1) (Superoxide dismutase 1) (hSod1) |
| Q9UPU3 | SORCS3 | VPS10 domain-containing receptor SorCS3 |
| P09486 | SPARC | SPARC (Basement-membrane protein 40) (BM-40) (Osteonectin) (ON) (Secreted protein acidic and rich in cysteine) |
| Q14515 | SPARCL1 | SPARC-like protein 1 (High endothelial venule protein) (Hevin) (MAST 9) |
| Q08629 | SPOCK1 | Testican-1 (Protein SPOCK) |
| Q9HCB6 | SPON1 | Spondin-1 (F-spondin) (Vascular smooth muscle cell growth-promoting factor) |
| P10451-5 | SPP1 | Osteopontin (Bone sialoprotein 1) (Nephropontin) (Secreted phosphoprotein 1) (SPP-1) (Urinary stone protein) (Uropontin) |
| O60279 | SUSD5 | Sushi domain-containing protein 5 |
| Q01995 | TAGLN | Transgelin (22 kDa actin-binding protein) (Protein WS3-10) (Smooth muscle protein 22-alpha) (SM22-alpha) |
| P20062 | TCN2 | Transcobalamin-2 (TC-2) (Transcobalamin II) (TC II) (TCII) |
| Q15582 | TGFBI | Transforming growth factor-beta-induced protein ig-h3 (Beta ig-h3) (Kerato-epithelin) (RGD-containing collagen-associated protein) (RGD-CAP) |
| O43493 | TGOLN2 | Trans-Golgi network integral membrane protein 2 (TGN38 homolog) (TGN46) (TGN48) (Trans-Golgi network protein TGN51) |
| P04216 | THY1 | Thy-1 membrane glycoprotein (CDw90) (Thy-1 antigen) (CD antigen CD90) |
| P01033 | TIMP1 | Metalloproteinase inhibitor 1 (Erythroid-potentiating activity) (EPA) (Fibroblast collagenase inhibitor) (Collagenase inhibitor) (Tissue inhibitor of metalloproteinases 1) (TIMP-1) |
| P16035 | TIMP2 | Metalloproteinase inhibitor 2 (CSC-21K) (Tissue inhibitor of metalloproteinases 2) (TIMP-2) |
| Q24JP5-2 | TMEM132A | Transmembrane protein 132A (HSPA5-binding protein 1) |
| Q92752 | TNR | Tenascin-R (TN-R) (Janusin) (Restrictin) |
| P22105-4 | TNXB | Tenascin-X (TN-X) (Hexabrachion-like protein) |
| P60174 | TPI1 | Triosephosphate isomerase (TIM) (EC 5.3.1.1) (Triose-phosphate isomerase) |
| O14773 | TPP1 | Tripeptidyl-peptidase 1 (TPP-1) (EC 3.4.14.9) (Cell growth-inhibiting gene 1 protein) (Lysosomal pepstatin-insensitive protease) (LPIC) (Tripeptidyl aminopeptidase) (Tripeptidyl-peptidase I) (TPP-I) |
| Q9NZC2 | TREM2 | Triggering receptor expressed on myeloid cells 2 (TREM-2) (Triggering receptor expressed on monocytes 2) |
| P10599 | TXN | Thioredoxin (Trx) (ATL-derived factor) (ADF) (Surface-associated sulphydryl protein) (SASP) |
| Q06418 | TYRO3 | Tyrosine-protein kinase receptor TYRO3 (EC 2.7.10.1) (Tyrosine-protein kinase BYK) (Tyrosine-protein kinase DTK) (Tyrosine-protein kinase RSE) (Tyrosine-protein kinase SKY) (Tyrosine-protein kinase TIF) |
| Q6EMK4 | VASN | Vasorin (Protein slit-like 2) |
| P19320 | VCAM1 | Vascular cell adhesion protein 1 (V-CAM 1) (VCAM-1) (INCAM-100) (CD antigen CD106) |
| P13611 | VCAN | Versican core protein (Chondroitin sulfate proteoglycan core protein 2) (Chondroitin sulfate proteoglycan 2) (Glial hyaluronate-binding protein) (GHAP) (Large fibroblast proteoglycan) (PG-M) |
| O15240 | VGF | Neurosecretory protein VGF [Cleaved into: Neuroendocrine regulatory peptide-1 (NERP-1); Neuroendocrine regulatory peptide-2 (NERP-2); Antimicrobial peptide VGF[554-577]] |
| P08670 | VIM | Vimentin |
| Q9Y279 | VSIG4 | V-set and immunoglobulin domain-containing protein 4 (Protein Z39Ig) |
| Q8TAG5-2 | VSTM2A | V-set and transmembrane domain-containing protein 2A |
| Q8TEU8 | WFIKKN2 | WAP, Kazal, immunoglobulin, Kunitz and NTR domain-containing protein 2 (Growth and differentiation factor-associated serum protein 1) (GASP-1) (hGASP-1) (WAP, follistatin, immunoglobulin, Kunitz and NTR domain-containing-related protein) (WFIKKN-related protein) |
| P63104 | YWHAZ | 14-3-3 protein zeta/delta (Protein kinase C inhibitor protein 1) (KCIP-1) |
| P01708 | IGLV2-11 | Immunoglobulin lambda variable 2-11 |
| P83593 | IGKV4-1 | Immunoglobulin kappa variable 4-1 |
| P04438 | IGHV2-70 | Immunoglobulin heavy variable 2-70 |
