# Supplementary material for: Proteomic changes in cerebrospinal fluid from primary central nervous system lymphoma patients are associated with protein ectodomain shedding
Source: Oncotarget. 2017 Nov 24;8(66):110118–32. doi: 10.18632/oncotarget.22654 (PMC5746369; doi:10.18632/oncotarget.22654)
Supplement: Supplementary file 6 [file oncotarget-08-110118-s006.docx]

**Supplementary Table 6: Network enrichment analysis of 66 significantly altered proteins.**

| **GOID** | **GOTerm** | **Term PValue** | **Term PValue Corrected with Benjamini-Hochberg** | **Group PValue** | **Group PValue Corrected with Benjamini-Hochberg** | **GOGroups** | **% Associated Genes** | **Nr. Genes** | **Associated Genes Found** |
| --- | --- | --- | --- | --- | --- | --- | --- | --- | --- |
| GO:0021700 | developmental maturation | 16.0E-3 | 41.0E-3 | 18.0E-3 | 22.0E-3 | Group0 | 6.41 | 5.00 | [ADGRL1, AGRN, RELN, SEZ6, SEZ6L2] |
| GO:0030534 | adult behavior | 2.5E-3 | 23.0E-3 | 18.0E-3 | 22.0E-3 | Group0 | 10.00 | 5.00 | [EPHA4, NRXN2, PCDH17, SEZ6, SEZ6L2] |
| GO:0050803 | regulation of synapse structure or activity | 5.0E-3 | 32.0E-3 | 18.0E-3 | 22.0E-3 | Group0 | 6.31 | 7.00 | [ADGRB2, ADGRL1, AGRN, BCAN, PCDH17, RELN, SORCS3] |
| GO:0050808 | synapse organization | 1.9E-3 | 36.0E-3 | 18.0E-3 | 22.0E-3 | Group0 | 6.67 | 8.00 | [ADGRB2, ADGRL1, AGRN, NRXN2, PCDH17, RELN, SEZ6, SEZ6L2] |
| GO:0008344 | adult locomotory behavior | 23.0E-3 | 48.0E-3 | 18.0E-3 | 22.0E-3 | Group0 | 9.38 | 3.00 | [EPHA4, SEZ6, SEZ6L2] |
| GO:0050807 | regulation of synapse organization | 7.8E-3 | 33.0E-3 | 18.0E-3 | 22.0E-3 | Group0 | 7.69 | 5.00 | [ADGRB2, ADGRL1, AGRN, PCDH17, RELN] |
| GO:0007612 | learning | 9.6E-3 | 30.0E-3 | 18.0E-3 | 22.0E-3 | Group0 | 9.09 | 4.00 | [NPTX2, NRXN2, RELN, SORCS3] |
| GO:0051962 | positive regulation of nervous system development | 15.0E-3 | 42.0E-3 | 18.0E-3 | 22.0E-3 | Group0 | 4.71 | 8.00 | [ADGRB2, ADGRL1, AGRN, EPHA4, RELN, RGMA, SEMA6A, SEZ6] |
| GO:0007268 | synaptic transmission | 17.0E-3 | 40.0E-3 | 18.0E-3 | 22.0E-3 | Group0 | 4.33 | 9.00 | [ADGRL1, AGRN, BCAN, NPTX2, NRXN2, PCDH17, RELN, SEZ6, SORCS3] |
| GO:0010977 | negative regulation of neuron projection development | 2.2E-3 | 28.0E-3 | 18.0E-3 | 22.0E-3 | Group0 | 8.45 | 6.00 | [EPHA4, LINGO1, RGMA, RTN4R, SEMA6A, SEZ6] |
| GO:1901136 | carbohydrate derivative catabolic process | 22.0E-3 | 49.0E-3 | 6.4E-3 | 16.0E-3 | Group1 | 5.95 | 5.00 | [AGRN, BCAN, CECR1, HEXB, IDS] |
| GO:1901565 | organonitrogen compound catabolic process | 7.8E-3 | 29.0E-3 | 6.4E-3 | 16.0E-3 | Group1 | 5.30 | 8.00 | [AGRN, BCAN, CAT, CECR1, CPQ, HEXB, IDS, QDPR] |
| GO:0006027 | glycosaminoglycan catabolic process | 8.9E-3 | 30.0E-3 | 6.4E-3 | 16.0E-3 | Group1 | 9.30 | 4.00 | [AGRN, BCAN, HEXB, IDS] |
| GO:1903510 | mucopolysaccharide metabolic process | 6.1E-3 | 29.0E-3 | 6.4E-3 | 16.0E-3 | Group1 | 6.90 | 6.00 | [AGRN, BCAN, HEXB, IDS, IMPAD1, ITIH5] |
| GO:0046426 | negative regulation of JAK-STAT cascade | 4.5E-3 | 34.0E-3 | 16.0E-3 | 26.0E-3 | Group2 | 16.67 | 3.00 | [CHAD, RTN4R, RTN4RL2] |
| GO:1904030 | negative regulation of cyclin-dependent protein kinase activity | 14.0E-3 | 42.0E-3 | 16.0E-3 | 26.0E-3 | Group2 | 11.11 | 3.00 | [CHAD, RTN4R, RTN4RL2] |
| GO:0051241 | negative regulation of multicellular organismal process | 5.6E-3 | 30.0E-3 | 5.6E-3 | 28.0E-3 | Group3 | 4.08 | 14.00 | [ADGRB2, C1QC, CHAD, EPHA4, GPNMB, HLA-DRB1, LBP, LINGO1, PTPRG, RGMA, RTN4R, SEMA6A, SEZ6, VSIG4] |
| GO:0051962 | positive regulation of nervous system development | 15.0E-3 | 42.0E-3 | 5.6E-3 | 28.0E-3 | Group3 | 4.71 | 8.00 | [ADGRB2, ADGRL1, AGRN, EPHA4, RELN, RGMA, SEMA6A, SEZ6] |
| GO:0010977 | negative regulation of neuron projection development | 2.2E-3 | 28.0E-3 | 5.6E-3 | 28.0E-3 | Group3 | 8.45 | 6.00 | [EPHA4, LINGO1, RGMA, RTN4R, SEMA6A, SEZ6] |
| GO:0031099 | regeneration | 22.0E-3 | 49.0E-3 | 22.0E-3 | 22.0E-3 | Group4 | 5.95 | 5.00 | [CPQ, EPHA4, PTPRF, RGMA, RTN4RL2] |
| GO:0031103 | axon regeneration | 1.1E-3 | 45.0E-3 | 22.0E-3 | 22.0E-3 | Group4 | 16.00 | 4.00 | [EPHA4, PTPRF, RGMA, RTN4RL2] |
